# Supplementary material for: Prediction models for prostate cancer to be used in the primary care setting: a systematic review
Source: BMJ Open. 2020 Jul 19;10(7):e034661. doi: 10.1136/bmjopen-2019-034661 (PMC7371149; doi:10.1136/bmjopen-2019-034661)
Supplement: Supplementary data [file bmjopen-2019-034661supp001.pdf]

Search Strategy

1) * Prostatic Neoplasms/
2) Initial biopsy.mp
3) First biopsy.mp
4) * “Early Detection of Cancer”/mt [Methods]
5) 2 or 3 or 4
6) 1 and 5
7) Nomograms/
8) “Neural Networks (Computer)”/
9) Risk Assessment/
10) Models, Statistical/
11) 7 or 8 or 9 or 10
12) 6 and 11
13) Limit 12 to English language
14) Limit 13 to abstracts
