# Supplementary material for: Prediction models for prostate cancer to be used in the primary care setting: a systematic review
Source: BMJ Open. 2020 Jul 19;10(7):e034661. doi: 10.1136/bmjopen-2019-034661 (PMC7371149; doi:10.1136/bmjopen-2019-034661)
Supplement: Supplementary data [file bmjopen-2019-034661supp002.pdf]

***Supplementary File 2: Search results for independent studies that did external validation for included models.***

| Study | No. of citations in Google Scholar | No. of citations in Medline | No. of external validation found in Google Scholar | No. of external validation found in Medline |
|---|---|---|---|---|
| Carlson, 1998 | 117 | 20 | Null | Null |
| Babaian, 2000 | 128 | 13 | Null | Null |
| Jansen, 2010 | 231 | 44 | Null | Null |
| Hill, 2010 | 5 | 0 | Null | Null |
| Lazzeri, 2013 | 195 | 0 | Null | Null |
