# Supplementary material for: Prediction models for prostate cancer to be used in the primary care setting: a systematic review
Source: BMJ Open. 2020 Jul 19;10(7):e034661. doi: 10.1136/bmjopen-2019-034661 (PMC7371149; doi:10.1136/bmjopen-2019-034661)
Supplement: Supplementary data [file bmjopen-2019-034661supp003.pdf]

Supplementary File 3: Type of assays used to measure PSA and free PSA.

Study	PSA Assay	Free PSA Assay
Carlson, 1998 [1]	PA immunoassay (Tosoh)	Investigational doubleantibody radioimmunometric assay (PSA II, DIANON Systems)
Babaian, 2000 [2]	PSA immunometric assay (Tosoh)	Tandem R assay (Beckman Coulter, San Diego, Calif)
Jansen, 2010 [3]	Access 2 Immunoassay System (Beckman Coulter, Brea, CA, USA)	Access 2 Immunoassay System (Beckman Coulter, Brea, CA, USA)
Hill, 2013 [4]	N/A	N/A
Lazzeri, 2013 [5]	Access 2 Immunoassay System (Beckman Coulter, Brea, CA, USA)	Access 2 Immunoassay System (Beckman Coulter, Brea, CA, USA)

References:

1. Carlson GD, Calvanese CB, Partin AW. An algorithm combining age, total prostate-specific antigen (PSA), and percent free PSA to predict prostate cancer: Results on 4298 cases. *Urology* 1998;52:455-61. doi: 10.1016/S0090-4295(98)00205-2
2. Babaian RJ, Fritsche H, Ayala A, et al. Performance of a neural network in detecting prostate cancer in the prostate-specific antigen reflex range of 2.5 to 4.0 ng/mL. *Urology* 2000;56:1000-6.
3. Jansen FH, van Schaik RHN, Kurstjens J, et al. Prostate-Specific Antigen (PSA) Isoform p2PSA in Combination with Total PSA and Free PSA Improves Diagnostic Accuracy in Prostate Cancer Detection. *European Urology* 2010;57:921-27. doi: 10.1016/j.eururo.2010.02.003
4. Hill OT, Mason TJ, Schwartz SW, et al. Improving prostate cancer detection in veterans through the development of a clinical decision rule for prostate biopsy. *BMC urology* 2013;13 doi: 10.1186/1471-2490-13-6
5. Lazzeri M, Haese A, de la Taille A, et al. Serum Isoform [−2]proPSA Derivatives Significantly Improve Prediction of Prostate Cancer at Initial Biopsy in a Total PSA Range of 2–10 ng/ml: A Multicentric European Study. *European Urology* 2013;63:986-94. doi: 10.1016/j.eururo.2013.01.011
