# Supplementary material for: Prediction models for prostate cancer to be used in the primary care setting: a systematic review
Source: BMJ Open. 2020 Jul 19;10(7):e034661. doi: 10.1136/bmjopen-2019-034661 (PMC7371149; doi:10.1136/bmjopen-2019-034661)
Supplement: Author's manuscript [file bmjopen-2019-034661.draft_revisions.pdf]

BMJ Open

BMJ Open is committed to open peer review. As part of this commitment we make the peer review history of every article we publish publicly available.

When an article is published we post the peer reviewers' comments and the authors' responses online. We also post the versions of the paper that were used during peer review. These are the versions that the peer review comments apply to.

The versions of the paper that follow are the versions that were submitted during the peer review process. They are not the versions of record or the final published versions. They should not be cited or distributed as the published version of this manuscript.

BMJ Open is an open access journal and the full, final, typeset and author-corrected version of record of the manuscript is available on our site with no access controls, subscription charges or pay-per-view fees (http://bmjopen.bmj.com).

If you have any questions on BMJ Open's open peer review process please email info.bmjopen@bmj.com

BMJ Open

Non-clinical and non-genetic risk prediction models for prostate cancer: a systematic review

Journal:	*BMJ Open*
Manuscript ID	bmjopen-2019-034661
Article Type:	Original research
Date Submitted by the Author:	02-Oct-2019

Complete List of Authors:	Aladwani , Mohammad; The University of Manchester, Division of Population Health, Health Services Research and Primary Care School of Health Sciences Faculty of Biology, Medicine and Health<br>Lophatananon, Artitaya ; The University of Manchester, Division of Population Health, Health Services Research and Primary Care School of Health Sciences Faculty of Biology, Medicine and Health<br>Ollier, William; The University of Manchester, Division of Population Health, Health Services Research and Primary Care School of Health Sciences Faculty of Biology, Medicine and Health; Manchester Metropolitan University Faculty of Science and Engineering, School of Healthcare Science<br>Muir, Kenneth; The University of Manchester, Division of Population Health, Health Services Research and Primary Care School of Health Sciences Faculty of Biology, Medicine and Health

Keywords:	EPIDEMIOLOGY, UROLOGY, Prostate disease < UROLOGY

SCHOLARONE™
Manuscripts

I, the Submitting Author has the right to grant and does grant on behalf of all authors of the Work (as defined in the below author licence), an exclusive licence and/or a non-exclusive licence for contributions from authors who are: i) UK Crown employees; ii) where BMJ has agreed a CC-BY licence shall apply, and/or iii) in accordance with the terms applicable for US Federal Government officers or employees acting as part of their official duties; on a worldwide, perpetual, irrevocable, royalty-free basis to BMJ Publishing Group Ltd ("BMJ") its licensees and where the relevant Journal is co-owned by BMJ to the co-owners of the Journal, to publish the Work in this journal and any other BMJ products and to exploit all rights, as set out in our licence.

The Submitting Author accepts and understands that any supply made under these terms is made by BMJ to the Submitting Author unless you are acting as an employee on behalf of your employer or a postgraduate student of an affiliated institution which is paying any applicable article publishing charge ("APC") for Open Access articles. Where the Submitting Author wishes to make the Work available on an Open Access basis (and intends to pay the relevant APC), the terms of reuse of such Open Access shall be governed by a Creative Commons licence – details of these licences and which Creative Commons licence will apply to this Work are set out in our licence referred to above.

Other than as permitted in any relevant BMJ Author's Self Archiving Policies, I confirm this Work has not been accepted for publication elsewhere, is not being considered for publication elsewhere and does not duplicate material already published. I confirm all authors consent to publication of this Work and authorise the granting of this licence.

For peer review only

Full title: Non-clinical and non-genetic risk prediction models for prostate cancer: a systematic review

Institutional addresses and e-mail addresses of the contributing authors:

Dr Mohammad Aladwani

Division of Population Health, Health Services Research and Primary Care, School of Health Sciences Faculty of Biology, Medicine and Health, The University of Manchester, Manchester, UK, M13 9PT

E-mail: mohammad.al-adwani@postgrad.manchester.ac.uk

Dr Artitaya Lophatananon

Division of Population Health, Health Services Research and Primary Care, School of Health Sciences Faculty of Biology, Medicine and Health, The University of Manchester, Manchester, UK, M13 9PT

E-mail: Artitaya.lophatananon@.manchester.ac.uk

Professor William Ollier,

Division of Population Health, Health Services Research and Primary Care, School of Health Sciences Faculty of Biology, Medicine and Health, The University of Manchester, Manchester, UK, M13 9PT;

School of Healthcare Science, Faculty of Science and Engineering, Manchester Metropolitan University, John Dalton Building Manchester M1 5GD UK

E-mail: Bill.Oliier@manchester.ac.uk

Address and contact details of the corresponding author:

Professor Kenneth Muir

Division of Population Health, Health Services Research and Primary Care,

School of Health Sciences Faculty of Biology, Medicine and Health,

The University of Manchester, Manchester,

UK, M13 9PT

For peer review only

E-mail: Kenneth.muir@manchester.ac.uk

Telephone: +44-161-2785677

Key words: Prostate cancer, Prostate specific antigen, PSA, Prostate cancer screening, Risk Prediction, Risk tools, Prediction models

For peer review only

Abstract

Objective

To identify and compare existing risk prediction models for prostate cancer (PCa) that have a potential use in a primary care and community health hub settings.

Design

Systematic review.

Data sources

MEDLINE and EMBASE databases combined up to, and including, January 2019.

Eligibility

Studies were considered relevant based on the following criteria; (i) presenting an evaluation of prospective risk of PCa at initial biopsy, (ii) patients having no history of PCa, (iii) studies not incorporating an invasive clinical assessment or a predictive genetic test, (iv) inclusion of at least two variables with PSA being one of them, and (v) studies reporting a measure of predictive performance.

Results

An initial search yielded 109 studies, of which five met the set criteria. Four studies were cohort-based and one was a case-control study. One study used an artificial neural network (ANN) to build the prediction model, and the remaining four studies applied logistic regression methodology. The sample sizes analysed within these studies ranged from 151 to 3,773 subjects. After prostate specific antigen (PSA), the most common model inputs were free PSA (fPSA), free-to-total PSA ratio (%fPSA) and age. PCa detection rate was between 20.6% and 55.8%. AUC was reported in four studies and ranged from 0.65 to 0.75. All models showed significant improvement in predicting PCa compared with being just based on PSA alone. The difference in AUC between extended models and PSA alone was between 0.06 and 0.21.

Conclusion

Only a few PCa risk prediction models have the potential to be used in the primary health care / community health centres. The key elements for any model considered to

For peer review only

be of use at a community level are cost, feasibility and its performance for predicting PCa. Of all the models we investigated, issues around model development were identified. Despite increased sensitivity and specificity being achieved using models that incorporate other variables than just PSA alone, model calibration was poorly reported and external validation was carried out in only one study. Further studies are needed to investigate other potential variables that could be integrated into models to improve their clinical utility for PCa screening in a community-based setting.

For peer review only

INTRODUCTION

Prostate cancer (PCa) is the second most common cancer and the fifth leading cause of cancer-attributed death in men worldwide with an estimated incidence of 1,276,106 and 358,989 deaths in 2018 [1]. In the UK, around 47,200 new cases of PCa were reported in 2015, accounting for 26% of all new cancer cases in males. PCa had a mortality within the UK of around 11,600 in 2016 [2]. The global projections of PCa incidence and mortality for 2030 are 1.7 and 0.5 million, respectively [3]. The highest incidence of PCa is seen in western societies [4]. The significant increase of PCa incidence and diagnosis over the last three decades can be attributed mainly to the widespread implementation of the PSA serum test after it had been introduced in the late 1980s [5 6].

The strong association of PSA with PCa [7 8], along with it being a relatively inexpensive test [9], made PSA a key biomarker in the diagnostic processs of PCa and the recommendation of a confirmatory prostate biopsy [7 9]. PSA is, however, not a cancer-specific marker [5 10]. Conditions such as benign prostate hypertrophy (BPH), prostatitis, and other non-malignant prostatic conditions can also elevate PSA level, thus introducing uncertainty to the application of the test [11-14]. This highlights some limitations of the PSA test regarding its specificity and sensitivity and it being largely dependent on setting a 'diagnostic' cut-off point, which often leads to an unacceptable number of false-positive and false-negative results [5 10 15 16]. Such issues are likely to be the explanation for the significant number of unnecessary biopsies currently performed each year. Such procedures are associated with adverse side effects for patients and also increases health care costs [17 18].

To address such PSA test limitations, researchers have incorporated other measurable factors into approaches for the early detection of PCa; these "risk assessment tools" are based on statistical models designed to improve the accuracy and performance of the PSA test [19-22]. Logistic regression and artificial neural network (ANNs) models are now considered to be the most common and effective statistical techniques in aiding confident early PCa diagnosis [23]. An important advantage of these models is their ability to tailor accurate estimation of PCa risk individually. This may ultimately lead to their use in patient counselling and decision-making [24-27]. Such models have already

For peer review only

achieved better results in predicting probabilities of outcome than clinical judgment [28 29]. Furthermore, it has been reported that using such predictive models may minimize the rate of unnecessary biopsies [30]. Recently there has been a substantial increase in the development of predictive models to help clinicians assess risk and make a diagnosis of PCa [22 26 30-35]. The majority of these models are designed for use in clinical settings and most include the need for a clinical examination. Some of these procedures, such as digital rectal examination (DRE) or trans-rectal ultrasonography (TRUS), are considered invasive and to potentially cause anxiety [36]. As a result, men may feel reluctant to have such tests performed.

This systematic review of the literature was undertaken to identify risk prediction models that do not incorporate invasive clinical procedures or genetic variables but have potential application for use in primary care and community settings. The performance of the models reviewed for detecting PCa at initial biopsy have been compared using 'reported area under the curve' (AUC) and/or sensitivity - specificity testing.

METHODS

The approach used to identify and select relevant articles was based on the application of the '*Preferred Reporting Items for Systematic Reviews and Meta-Analysis'* (PRISMA) [37].

Data sources and search strategy

A literature search was performed using Medline and Embase databases. The 'medical subject heading' (MeSH) terms, combined with Boolean logic operators 'AND' and 'OR', were applied to retrieve relevant articles. The terms used for the search were "Prostatic Neoplasms" AND ("Initial biopsy" OR "First biopsy" OR "Early detection of cancer") AND ("Nomograms" OR "Artificial neural networks" OR "Risk assessment" OR "Statistical model"). All articles defined (published up to the end of January 2019) were subsequently further filtered as being those only published in English language and with an abstract. Further to using the above search databases, appropriate research articles were selected manually from the reference lists of any relevant review articles.

For peer review only - http://bmjopen.bmj.com/site/about/guidelines.xhtml

Eligibility criteria

As this review focuses on PCa risk prediction based in community healthcare settings, all studies were selected on the following inclusion criteria (i) evaluating the prospective risk for PCa at initial biopsy (ii) study subjects used in the development of risk prediction models had no prior history of PCa (iii) studies that reported risk assessment tools not using genetic or “invasive” clinical tests/examinations such as DRE or TRUS (iv) studies that included a minimum of two variables of which PSA had to be one of them [an elevated PSA test in UK primary care is usually the first sign and rationale for suggesting a diagnosis of PCa within NICE guidelines] and (v) studies that reported AUC and/or sensitivity and specificity of the diagnostic/predictive tool. The exclusion criteria used were (i) articles with models that were built and based on repeat or mixed biopsies (ii) studies that only validate an existing model (iii) articles that were not published in English. There were no time boundaries regarding the publication year.

Screening titles, abstract, and full-texts was carried out by one reviewer (MA). Any concerns about the eligibility of a study were resolved by discussion with the second reviewer (AL).

Data extraction

A data extraction form was developed to collect all relevant information. For each study used in this review, the items extracted included; year of publication, source of data, type of model, number of subjects, country where it was performed, age, PSA range, mean/median PSA, number of biopsy cores, variables included in the model, study endpoint(s), cancer detection, model validation, and model performance.

Evaluating the performance of the risk models

Prediction models can be evaluated against various criteria. The most critical measurements of model performance are discrimination and calibration [27]. Discrimination refers to how well the prediction model can differentiate subjects in different outcome classes according to their predicted risks. It is often assessed by measuring the area under the receiver operating characteristic (ROC) curve [38]. It also requires setting a series of thresholds to separate low and high ranges of predicted outcomes. A value of 0.5 indicates no discrimination, while a value of 1 indicates perfect

For peer review only

discrimination. However, even with perfect discrimination, observed risk can differ from expected risk. Therefore, calibration has an important role in model evaluation [39]. Calibration represents the agreement between expected and observed outcomes [40]. A well-calibrated model is achieved when the calibration slope is close to 1. When the calibration slope is less than 1 it indicates that the model under-estimates low risks and over-estimate high risks [41].

Due to the heterogeneity of the studies included, conducting a meta-analysis was not applicable.

Study quality assessment

The quality of the studies included in this review was assessed using the PROBAST tool [42]. This tool has been developed specifically to assess the risk of bias and applicability for prediction model studies. The tool consists of four domains and has 20 signalling questions that facilitate to reach overall judgment of risk of bias, as well as issues relating to applicability.

Patient and Public involvement

No patients were involved in setting the research question or the outcome measures, nor were they involved in the design and implementation of the study. There are no plans to involve patients in dissemination.

RESULTS

A total of 102 publications were identified using the search strategy as shown in Fig.1. An additional nine articles were identified through manual searches from a bibliography of reviewed articles. At the first filter step, a total of 109 titles and abstracts were screened for eligibility after removing two duplicates. In the second filter step, 60 papers failed to meet the inclusion criteria and were excluded, resulting in 49 articles. The final step of filtering yielded only five studies that were considered to be eligible (i.e. passed all set criteria) and were thus included in this systematic review.

Study characteristics

Four of the five studies included were cohort studies and one was a case-control study. The characteristics of each of these studies and populations are summarised in Table 1.

For peer review only

Table 1: Characteristics of the included studies

Author & year	Type of model	Type of study	Sample No.	Location	Population type	Age	PSA range	Median PSA	No. of biopsy cores	Cancer detection
Carlson, 1998 [43]	Logistic regression	Cohort	Model dev = 3773<br>Validation = 525	Baltimore, USA	Referral	≥ 45	4-20 ng/ml	—	Sextant biopsy	32%
Babaian, 2000 [44]	Neural network = 3 ANNs	Cohort	151	Texas, USA	Screening program	40-75	2.5-4 ng/ml	—	11 cores	24.5%
Jansen, 2010 [45]	Logistic regression	Cohort	Site 1 = 405<br>Site 2 = 351	Site 1 from the Rotterdam arm of the European Study of screening for Prostate cancer<br>Site 2 Innsbruck, Austria	Screening program	≥ 50	2-10 ng/ml	~ 4.4	≥6 cores	Site 1 = 55.8%<br>Site 2 = 49.6%
Hill, 2013 [46]	Logistic regression	Case-control	1378	Florida, USA	Hospital referral	40-90	≥ 4 ng/ml	—	N/A	20.6%
Lazzeri, 2013 [47]	Logistic regression	Cohort	646	European multi-centre; Italy, Germany, France, Spain, and the UK	Referral	>45	2-10 ng/ml	~ 5.8	≥ 12 cores	40.1%

Subjects used to build the risk models varied across these studies. Of the five studies, three studies included men from referral populations [43 46 47] and two studies from screening programs [44 45]. The sample sizes ranged from 151 to 3,773 with three studies derived from US cohorts [43 44 46] and two from Europe [45 47]. Four studies used logistic regression methodology to build their model, whereas one study used an artificial neural network-based approach (ANN) [44]. The minimum age of participants was 40 years old [44 46] and the minimum PSA level was 2 ng/ml [45 47].

Variables in the model

Table 2 presents details of the variables used in each model. PSA level was used in all models, followed by free PSA (fPSA), age, and free-to-total PSA ratio (%fPSA). Other variables also reported in the models included; precursor of prostate-specific antigen (p2PSA), percentage of p2PSA to free PSA (%p2PSA), prostate health index (PHI), levels of haemoglobin, albumin, creatinine and red blood cell count (RBC), haematuria, mean corpuscular volume (MCV), and prostatic acid phosphatase.

For peer review only

Table 2: Variables used in the prostate cancer risk prediction models*

Author & year	Variables used in the model				
	**Total PSA**	**Free PSA**	**Percent free PSA**	**Age**	**Other variables**
Carlson, 1998 [43]	✓		✓	✓	
Babaian, 2000 [44]	✓	✓		✓	Creatinine Kinase, Prostatic acid phosphatase
Jansen, 2010 [45] *(Site 1)*	✓	✓			p2PSA
Jansen, 2010 [45] *(Site 2)*	✓	✓			p2PSA
Hill, 2013 [46] *(Method 1)*	✓			✓	HGB, RBC, Haematuria, Creatinine, MCV, and ethnicity “Black”
Hill, 2013 [46] *(Method 2)*	✓			✓	HGB, RBC, Creatinine, and MCV
Lazzeri, 2013 [47] *(Model 1)*	✓	✓	✓		
Lazzeri, 2013 [47] *(Model 2)*	✓	✓	✓		p2PSA
Lazzeri, 2013 [47] *(Model 3)*	✓	✓	✓		%p2PSA
Lazzeri, 2013 [47] *(Model 4)*	✓	✓	✓		PHI

*HGB= Haemoglobin. RBC= Red blood cells. MCV= Mean corpuscular volume. PHI= Prostate Health Index. p2PSA= Precursor of prostate specific antigen, %p2PSA= p2PSA/fPSA

For peer review only - http://bmjopen.bmj.com/site/about/guidelines.xhtml

Outcome

Study endpoint also varied among the studies selected. Two studies evaluated the accuracy of detecting any PCa [43 44] and three studies examined the pathologic Gleason score [45-47]. Although Jansen et al did not build a model to predict the aggressiveness of prostate cancer, they assessed the relationship of each variable individually with a Gleason score ≥7. PCa was determined by taking a needle biopsy. All subjects in the five studies underwent prostate biopsy. The least number of biopsy cores used were 6 [43 45] and the highest were ≥ 12 [47]. One study did not report the number of biopsy cores taken [46]. PCa rates ranged from 20.6% to 55.8%.

Evaluating the performance of the risk models

For predicting any PCa, the Jansen et al model used data from the Rotterdam arm of the European Study of Screening for PCa (ESPRC). Their model achieved the highest discrimination value when compared to PSA alone (AUC of 0.755 vs. 0.585, respectively) [45]. The AUC values in other studies ranged from 0.648 to 0.74. Model performance is presented in Fig 2.

One study did not provide the AUC but instead reported an increase of 11% in specificity over percent free PSA alone with 95% sensitivity [43]. Lazzeri et al, [47] presented results from four separated models discriminating PCa with a Gleason score of ≥7. Both model 2 (which includes the base model tPSA, fPSA and %fPSA in addition to p2PSA) and model 3 (which includes base model plus PHI) show the highest levels of discrimination with an AUC of 0.67. In the study of Hill [46] the authors classified PCa stages differently and built their two models accordingly. In their first model, the difference in the discrimination was analysed and based on all PCa versus non-cancerous prostate conditions where the AUC for this model was 0.68 compared to 0.59 for PSA alone. In the second model, the discrimination analysis was based on PCa stages II, III, IV versus PCa stage I, prostatic interstitial neoplasm (PIN), BPH and prostatitis where stages I, II, III and IV are parallel to T1, T2, T3/T4, and metastatic PCa respectively. The AUC for the second model was 0.72 compared to 0.63 for PSA alone. In general, four studies examined the AUC with PSA alone and all reported a benefit

For peer review only

from the use of logistic regression or the trained ANN. The differences of AUC for PSA alone and the extended model are presented in Table 3.

Table 3: The difference of area under the curve (AUC) for PSA alone and extended model

Study	AUC for PSA	AUC for model	ΔAUC (Model – PSA)
Carlson [43]	NA	NA	NA
Babaian [44]	0.64 %fPSA	0.74	0.1
Jansen [45] (*site 1)*	0.58	0.75	0.17
Jansen [45] *(site 2)*	0.53	0.7	0.16
Hill [46] *(method 1)*	0.59	0.68	0.09
Hill [46] *(method 2)*	0.63	0.72	0.09
Lazzeri [47]	0.50 for any PC<br>0.54 for Gleason score ≥7	Model 1 = 0.65<br>Model 1 (Gleason score≥7) = 0.60	0.15<br>0.06
		Model 2= 0.71<br>Model 2 (Gleason score≥7) = 0.67	0.21<br>0.13
		Model 3= 0.704<br>Model 3 (Gleason score≥7) = 0.67	0.2<br>0.13
		Model 4= 0.71<br>Model 4 (Gleason score≥7) = 0.672	0.21<br>0.13

Sensitivity and specificity data are presented in Table 4. At 95% sensitivity, the Babaian et al model shows the highest specificity (51%) whereas the Jansen model for both sites had the lowest specificity (~23.5%). In the Hill study, with a sensitivity of ~90%, the specificity was lower than in other studies (~18% and 28%) for method 1 and 2 respectively. In the study reported by Lazzeri, the sensitivity and specificity were not reported for the overall model; instead their study reports sensitivity and specificity for predictive variables individually. The highest sensitivity (90.5%) of %p2PSA and %fPSA achieved the highest specificity in predicting PCa at 21.5% and 22.8% respectively. Percentage p2PSA and PHI were more associated with Gleason scores.

For peer review only

Table 4: Sensitivity and specificity profile at different levels for each model

Study	Sensitivity	Specificity	Probability cut-off	Positive predictive value	Negative predictive value
Carlson [43]	99	18	>15	≤47	N/A
	95	34	18	51	NA
	89	43	20	42	NA
Babaian [44]	95	51	NA	39	97
	92	62	NA	44	96
	89	62	NA	43	95
Jansen [45] *(Site 1)*	95	23.9	NA	NA	NA
Jansen [45] *(Site 1)*	90	30.1	NA	NA	NA
Jansen [45] *(Site 2)*	95	23.2	NA	NA	NA
Jansen [45] *(Site 2)*	90	36.2	NA	NA	NA
Hill [46] *(Method 1)*	90.9	17.6	33	47.1	70.5
Hill [46] *(Method 2)*	89.8	28	13	20.6	91.3
Hill [46] *(Method 1)*	80.5	37.1	37	50.9	70.2
Hill [46] *(Method 2)*	78.2	45	15	28.7	88.8
Hill [46] *(Method 1)*	39.9	81.4	48	63.4	62.6
Hill [46] *(Method 2)*	45.8	79.5	23	36.7	85

* Lazzeri [47] model reported only sensitivity and specificity for predictive variables individually and at sensitivity of 90, %p2PSA and %fPSA achieved the highest specificity

For peer review only

Table 5 summarises the validation and calibration results for the studies analysed. Model calibration was reported in two studies [43 47]. Carlson plotted the observed and expected risks using calibration plots, whereas Lazzeri used the Hosmer-Lemeshow goodness-of-fit test. In terms of validation, two studies did not report model validation [45 46]. Only one study reported an external validation using an additional data set consisting of 525 patients [43]. Cross-validation using multiple re-sampling schemes was used in the Babaian Study, however, they did not report the number of time this was performed [44]. Lazzeri used 200 bootstrap re-samples to minimise overfitting bias [47].

Table 5: Validation and calibration for included models

Author & Year	Validation	Calibration
Carlson,1998 [43]	External validation on additional data set consisting of 525 patients	Calibration plot
Babaian,2000 [44]	Cross-validation and separate data set of 151	N/A
Jansen, 2010 [45]	N/A	N/A
Hill, 2013 [46]	N/A	N/A
Lazzeri, 2013 [47]	Internal validation using 200 bootstrap resamples	Internal calibration using the Hosmer-Lemeshow goodness-of-fit test

Study quality assessment

Quality assessment was carried out by two reviewers (MA and AL) with any discordance resolved by a third reviewer (KM). The assessment of results suggested some issue of study quality according to the criteria as set in the PROBAST tool, particularly in the analysis domain. For instance, one study applied univariable analysis to select predictors [43]. Three studies did not measure calibration [44-46]. Furthermore, two studies did not count for optimism and overfitting by using internal validation methods [45]

For peer review only

[46]. Whereas one study did not use appropriate measures for model performance i.e. AUC, this study reported the calibration [43].

The event per variable (EPV) was lower than recommended (< 10) in the Babaian [44] study indicating inadequate power. Four studies did not report missing data or how they handled it [43-45] [47]. The remaining study used complete-case analysis and excluded subjects with missing data on laboratory biomarkers (n=75) [46]. The PROBAST guidelines state that in a prediction model study where any risk of bias and applicability is low in all four domains, a regrading to high risk of bias should be considered when the study did not validate the model externally [42]. Thus, although the quality assessment for the Lazzeri study [47] was graded low risk in all the four domains, since the study did not report any external validation of the model, the assessment of the study has been regraded to high risk of bias. A full quality assessment for all studies is presented in Table 6.

For peer review only

Table 6: Quality assessment for risk of bias and applicability concern for included studies

Study	ROB				Applicability			Overall	
	Participants	Predictors	Outcome	Analysis	Participants	Predictors	Outcome	ROB	Applicability
Carlson [43]	+	+	-	-	+	+	-	-	-
Babaian [44]	+	+	+	-	-	+	+	-	-
Jansen [45]	-	-	-	-	+	-	-	-	-
Hill [46]	-	+	+	-	-	+	+	-	-
Lazzeri [47]	+	+	+	+	+	+	+	-	+

+ indicates a low risk of bias and applicability; - indicates a high risk of bias and applicability; ? indicates unclear bias and concern regarding applicability.

For peer review only

DISCUSSION

Despite the large number of PCa risk prediction models, the majority still include clinical inputs and/or genetic variants; few models exist that do not include specialist clinical input or genetic testing. To our knowledge, this is the first study to examine risk prediction models for PCa that do not include clinical and genetic variables.

Our study identified five unique models that met the set criteria. The Carlson model [43] has the largest population (3,773 subjects) when compared to the other four studies. Although they reported an 11% increase in specificity, they did not report AUC predictive estimates. It has been acknowledged that sensitivity and specificity results are dependent on the prevalence of the disease. Hence the comparison between populations where the PCa prevalence may vary (especially in early detection) will be difficult [33]. More importantly, by not reporting the AUC estimate, the model raises some doubts regarding the reliability of the model and its implementation [33]. It will also make comparison to other models not applicable [48].

Babaian [44] developed an algorithm and compared the performance of the ANN to PSA density (total PSA divided by prostate volume) (PSAD), %fPSA, and transition zone density (PSAD-TZ). Their ANN demonstrated a significant increase of model specificity that reached 51% when sensitivity was held at 95%. This was better than the specificity value of each individual variable such as %fPSA (10%), PSAD (39%), and PSAD-TZ (22%). In terms of AUC, the ANN achieved a moderate accuracy (0.74) being the second highest among all studies included. However, the ANN model did not show significant improvement when compared with a model fitted with only individual variables (AUC for %fPSA= 0.64, PSAD= 0.74, PSAD-TZ= 0.75). They included a number of uncommon pre-biopsy inputs into their algorithm such as prostatic acid phosphatase and creatinine kinase [49]. Furthermore, they used a tight PSA range (2.5-4.0ng/ml) which meant that their model may be less suitable for patients with PSA level below or above that range, thus limiting its generalisability.

The study by Jansen and colleagues [45] demonstrated that adding p2PSA to the base model of PSA and fPSA significantly enhanced the PCa predictive value and specificity. The association and added value of p2PSA in the prediction and detection of PCa have

been reported by several other studies [16 50-53]. Jansen [45] showed that p2PSA has no clear association with aggressive PCa. However, the base model that includes p2PSA had the highest clinical significance in correlation to pathologic Gleason score with a p-value of 0.008 compared to %fPSA and PHI (p-value 0.01 and 0.02) respectively. Although they used archived blood samples and retrospective analysis, the results were similar to a prospective study of 268 patients [16].

Hill [46] used a case-control study to evaluate several laboratory biomarkers. They found HGB, RBC, haematuria, creatinine, PSA, Age, MCV, and ethnicity ("being black") were statistically significantly associated in the first method ($p < 0.05$). In the second method, HGB, RBC, creatinine, PSA, age, and MCV were found to be statistically significantly correlated ($p <0.001$) with PCa. However, since this study was designed as a case-control study, it would have been prone to uncontrolled confounding and selection bias. Moreover, the type of screening protocols used in Veterans' Administrations may vary to those conducted in other healthcare systems; therefore, other populations may not have been represented. Furthermore, subjects with a PSA level < 4.0 ng/ml have not been investigated, and thus, the performance of the models are unknown for individuals in this group.

Lazzeri [47] in a European multi-centre study have evaluated similar biomarkers as in Jansen study with the same PSA range 2-10 ng/ml prospectively. They found no difference in both %p2PSA and PHI as individual PCa predictors with AUC of 0.67 (95% CI 0.64-0.71). However, the base model (consisting of PSA, fPSA, and %fPSA) that also included either p2PSA or PHI out-performed the base model alone and the base model including %p2PSA. In the analysis, the additive value of both p2PSA and PHI is 0.064 and 0.056 for %p2PSA for predicting the risk of PCa. These additive values increased to 0.076 for both p2PSA and PHI and 0.073 for %p2PSA in predicting Gleason scores ≥7 for the disease. The usefulness of PHI in improving the predictive accuracy of PCa over total and free PSA has been confirmed and reported by several studies [16 53-56].

In general, only one study has validated their model externally [43], whereas the remaining studies were either validated internally [44 47] or not reported any validation

For peer review only

methods [45 46]. Prediction models may not be equally applicable to all data sets as patients' characteristics may vary [20 57]. As a result, the generalisability of a model might be poor when it used in populations other than that used in building the model. Therefore, external validation should be conducted before applying any new model into general practice [58 59].

Another key performance measure of any model that needs careful evaluation is calibration. A calibration plot with a calibration slope is more preferable than the Hosmer-Lemeshow test; it has been acknowledged that evaluating a good and well calibrated model based on a large dataset can still fail the Hosmer-Lemeshow test. In contrast, when evaluating a poorly calibrated model with a small dataset it can still pass the Hosmer-Lemeshow test [60]. In our analysis, three studies fail to report the calibration of the model [44-46] whilst the Carlson study [43] used a calibration plot and Lazzeri [47] used the Hosmer-Lemeshow test. Excluding calibration from the majority of models may explain why some models are not currently used in practice [60].

With regard to biopsy cores, only two studies used extended biopsy cores. Babaian [44] used an 11-core multi-site biopsy, whereas Lazzeri [47] used at least 12 biopsy cores. Moreover, two studies used six cores biopsy in their model [43 45]. The use of six-core biopsy has been criticized as not being adequate in detecting PCa [61] and that models developed using sextant biopsy are less accurate than when a 10-core biopsy is used [62]. As a result, the European guideline for clinical PCa recommended an extended biopsy as standard practice for PCa detection [63].

It is worth noting that all five reviewed models performed better than just PSA alone. However, none of them has both high specificity and sensitivity. The level of sensitivity has been increased, and despite enhancement in the specificity, it is still considered low. Specificity is crucial when it comes to being used in a population setting as men without PCa should be ruled out as much as possible from further invasive engagement with the health system.

Our review suggests that none of the reviewed models provided an ideal performance in predicting PCa with high sensitivity and high specificity. It is particularly important when considering the application of PCa risk prediction at the population level that the tool

For peer review only

used should be able to both detect the outcome and filter out people with no disease. As there is robust evidence suggesting that the clinical relevance of PSA range to the detection of PCa differs across age groups [64-66], any future model should consider PSA threshold in relation to a specific age range. Risk prediction models for PCa should therefore take account of age.

Comparison with other studies

To our knowledge, three systematic reviews of PCa prediction tools have been published [20 26 27]. In the Louie et al. review, risk models were included that were externally validated in at least five study populations for the purpose of meta-analysis and only six studies were included in their analysis. Furthermore, all the studies included incorporated clinical tests such as DRE and/or TRUS-PV [26]. Schroder and Kattan [20] reviewed models that were built to predict the likelihood of having a positive prostate biopsy for cancer. However, it appears that they also included models where subjects had a previous negative biopsy. As such, some of the models included variables related to biopsy results and cores. The review by Shariat and colleagues examined different types of predictive tools [27]. They explored tools that predict PCa on initial and repeat biopsy, pathologic stages, biochemical recurrence after radical proctectomy, metastasis, survival, and life expectancy. Similarly, virtually all of the prediction tools that were based on initial biopsy included variables based on invasive procedures.

Strengths and limitations of this study

This report is the first to review risk prediction tools for PCa that can be used in primary care and community settings. Any prediction model should therefore be simple to use, based on non-invasive tests, be feasible at a population level and at low cost. We carried out an extensive data extraction relating to important features and characteristics for each study included, such as modelling method, source of data, sample size, variables, discrimination, validation, and cancer detection rate. We have also followed PRISMA guidelines for identifying eligible articles as well as for reporting this study. In addition, the PROBAST tool was adopted to assess the quality and risk of bias for each prediction model.

For peer review only

Our study has some limitations. Our aim was to identify prediction models that have the potential to be implemented in non-clinical settings, and consequently our search strategy was just focus to retrieve relevant studies for this dedicated purpose. Furthermore, we excluded articles that were not published in English or did not have an abstract. Moreover, studies that were only validating other existing models have not been included in this analyis. As a result of our strict inclusion criteria, our review includes a limited number of eligible studies.

A previous systematic review suggested that the majority of relevant studies could be identified through a manual search of articles reference lists instead of a database alone [20]. We identified four eligible studies using this approach. Given the small number of models identified by the approach we followed, that can be applied in primary care settings compared to the large number relating to wider existing models, it is unlikely that we have not included any study that would affect the results of our review.

Implications and future research

It is now accepted that the PSA test and its derivatives have some limitations for detecting PCa as defined by biopsy [67]. As a consequence, a considerable number of PCa prediction models have been built to improve prediction accuracy. This has resulted in a plethora of PCa risk prediction tools, with to date more than 100 models described [68 69]. There is evidence that some of these models show benefit and have better performance over just PSA measurement alone [20]. It also has been demonstrated that some of these models out-performed clinical experts in predicting PCa [28 29]. Although such models are not designed to replace specialist clinical judgment or patient preferences [57 67 70], they can help in patient counselling and aid clinicians to decide whether a prostate biopsy should be taken or not [58 70 71].

Given the small number of risk prediction models for PCa that do not incorporate clinical or genetic tests, the discrimination of these reviewed models ranged between poor to moderate (AUC range ~0.65 to ~0.75); in addition some issues relating to their study design and analysis raises the risk of bias. Consequently none of these models could be currently recommended for use in a primary care and community health care setting. Several guidelines are against using PSA test based screening for PCa; the US

For peer review only

Preventive Services task force, the Canadian task force on preventive health and the American College of Preventive Medicine do not currently recommend PSA-based testing due to insufficient evidence [72-74]. This has made it harder to convince policymakers to adopt PCa screening programme.

The first guideline of PROSTATE CANCER UK states *“In the future, health professionals should look at a man’s PSA level alongside other known risk factors as part of a risk assessment tool, when one becomes available.”* [75] However, the vast majority of the PCa risk prediction models are not suitable for routine use as they include clinical and genetic tests and are not validated externally in other cohorts. Therefore, the main challenge in the UK, is to develop a risk prediction tool that is reliable, cheap, is applicable for as wide an ethnicity as possible, and most importantly is easy to use and can be implemented at a primary care level [76].

The value of such risk tools is that they will help to stratify men at high risk of developing PCa earlier so that they have appropriate management and/or surveillance programme as early as possible. Such tools should help physicians have a better understanding of the risk for this disease and simplify the procedures and discussions with patients when recommending further specialist-led investigations. Furthermore, using the appropriate risk prediction tool will avoid men from undergoing inappropriate further and frequent testing [76]. This will reduce any associated costs of inappropriate tests and decrease the burden on health care delivery systems.

It is crucial to address these issues by identify all possible risk factors for PCa that are non-clinical, non-genetic, and easy to use and interpret. There is a pressing need to develop a risk prediction tool using all appropriate factors into a robust multivariable analysis and validate the model externally to eliminate applicability and generalisability concerns. Only when this is achieved will it be possible to introduce a prostate cancer screening programme fit for purpose.

CONCLUSION

There is a paucity of suitable models that incorporate non-clinical, non-genetic inputs and which can be used at a primary care level and in other community health services. Existing models have several limitations in both study design and reporting performance

For peer review only

measures. Future research should take into account these key issues and explore other risk factors.

ARTICLE SUMMARY

- There is abundance of risk models for prostate cancer that aimed to improve the performance of PSA test.
- The majority of these models are designed for use in clinical settings and some have issue with reliability and/or model validation.
- This is the first systematic review that focused on prostate cancer risk prediction that has the potential to be used in primary care and any other community health hubs.
- Few risk models that do not incorporate clinical and/or genetic tests and have potential to be applied in primary care and community settings.
- Future research must address all issues related to risk prediction model development and its potential application.

ACKNOWLEDGEMENT

MA is supported by Kuwait's Ministry of Health. KM and AL are supported by the NIHR Manchester Biomedical Research Centre and by the ICEP ("This work was also supported by CRUK [grant number C18281/A19169]).

AUTHOR CONTRIBUTIONS

MA, AL and KM were involved in study conception, idea and design. Data acquisition and extraction was obtained by MA. Data synthesis and interpretation was carried out by MA, AL and WO. MA and AL drafted the manuscript. All authors approved the final version of manuscript. KM is the study guarantor.

FUNDING

No funding is required for conducting this study.

COMPETING INTERESTS

All authors have completed the ICMJE uniform disclosure form at www.icmje.org/coi_disclosure.pdf and declare: no support from any organisation for the submitted work; no financial relationships with any organisations that might have an interest in the submitted work in the previous three years; no other relationships or activities that could appear to have influenced the submitted work.

ETHICAL APPROVAL

For peer review only

Not applicable as this study did not involve any personal data.

DATA SHARING

Data extraction sheet and the PROBAST assessment form for risk of bias is available upon request.

Figure legend

Figure 1: Flow diagram of studies included using PRISMA method

Figure 2: Model performance for each study using area under the curve (AUC)

For peer review only

REFERENCES

1. Bray F, Ferlay J, Soerjomataram I, et al. Global cancer statistics 2018: GLOBOCAN estimates of incidence and mortality worldwide for 36 cancers in 185 countries. *CA Cancer J Clin* 2018;68(6):394-424.

2. Cancer Research UK. Prostate cancer statistics 2017 [Available from: https://www.cancerresearchuk.org/health-professional/cancer-statistics/statistics-by-cancer-type/prostate-cancer#heading-One.

3. Center MM, Jemal A, Lortet-Tieulent J, et al. International variation in prostate cancer incidence and mortality rates. *Eur Urol* 2012;61(6):1079-92.

4. Zhou CK, Check DP, Lortet-Tieulent J, et al. Prostate cancer incidence in 43 populations worldwide: An analysis of time trends overall and by age group. *Int J Cancer* 2016;138(6):1388-400.

5. Dijkstra S, Mulders P, Schalken J. Clinical use of novel urine and blood based prostate cancer biomarkers: a review. *Clin Biochem* 2014;47(10-11):889-96.

6. Fontanella P, Benecchi L, Grasso A, et al. Decision-making tools in prostate cancer: from risk grouping to nomograms. *Minerva Urol Nefrol* 2017;69(6):556-66.

7. Stephan C, Ralla B, Jung K. Prostate-specific antigen and other serum and urine markers in prostate cancer. *Biochim Biophys Acta* 2014;1846(1):99-112.

8. Thompson IM, Pauler DK, Goodman PJ, et al. Prevalence of prostate cancer among men with a prostate-specific antigen level≤ 4.0 ng per milliliter. *N Engl J Med* 2004;350(22):2239-46.

9. Pereira-Azevedo N, Verbeek JFM, Nieboer D, et al. Head-to-head comparison of prostate cancer risk calculators predicting biopsy outcome. *Transl Androl Urol* 2018;7(1):18-26.

10. Stephan C, Cammann H, Meyer H-A, et al. PSA and new biomarkers within multivariate models to improve early detection of prostate cancer. *Cancer Lett* 2007;249(1):18-29.

11. Polascik TJ, Oesterling JE, Partin AW. Prostate specific antigen: a decade of discovery-what we have learned and where we are going. *J Urol* 1999;162(2):293-306.

12. Stamey TA, Caldwell M, McNEAL JE, et al. The prostate specific antigen era in the United States is over for prostate cancer: what happened in the last 20 years? *J Urol* 2004;172(4 Pt 1):1297-301.

13. Bunting PS. A guide to the interpretation of serum prostate specific antigen levels. *Clin Biochem* 1995;28(3):221-41.

14. Catalona WJ, Partin AW, Sanda MG, et al. A multi-center study of [− 2] pro-prostate-specific antigen (PSA) in combination with PSA and free PSA for prostate cancer detection in the 2.0 to 10.0 ng/mL PSA range. *J Urol* 2011;185(5):1650-55.

15. Mistry K, Cable G. Meta-analysis of prostate-specific antigen and digital rectal examination as screening tests for prostate carcinoma. *J AM Board Fam Pract* 2003;16(2):95-101.

For peer review only

16. Guazzoni G, Nava L, Lazzeri M, et al. Prostate-specific antigen (PSA) isoform p2PSA significantly improves the prediction of prostate cancer at initial extended prostate biopsies in patients with total PSA between 2.0 and 10 ng/ml: results of a prospective study in a clinical setting. *Eur Urol* 2011;60(2):214-22.

17. Heijnsdijk EA, der Kinderen A, Wever EM, et al. Overdetection, overtreatment and costs in prostate-specific antigen screening for prostate cancer. *Br J Cancer* 2009;101(11):1833-8.

18. Roobol MJ. Is prostate cancer screening bad or good? Summary of a debate at the innovation in urology meeting, September 17-19, 2010, Milan, Italy. *Eur Urol* 2011;59(3):359-62.

19. Mottet N, Bellmunt J, Bolla M, et al. EAU-ESTRO-SIOG Guidelines on Prostate Cancer. Part 1: Screening, Diagnosis, and Local Treatment with Curative Intent. *Eur Urol* 2017;71(4):618-29.

20. Schroder F, Kattan MW. The comparability of models for predicting the risk of a positive prostate biopsy with prostate-specific antigen alone: a systematic review. *Eur Urol* 2008;54(2):274-90.

21. Ross PL, Scardino PT, Kattan MW. A catalog of prostate cancer nomograms. *J Urol* 2001;165(5):1562-8.

22. Shariat SF, Karakiewicz PI, Suardi N, et al. Comparison of nomograms with other methods for predicting outcomes in prostate cancer: a critical analysis of the literature. *Clin Cancer Res* 2008;14(14):4400-7.

23. Hu X, Cammann H, Meyer HA, et al. Artificial neural networks and prostate cancer--tools for diagnosis and management. *Nat Rev Urol* 2013;10(3):174-82.

24. Caras RJ, Sterbis JR. Prostate cancer nomograms: a review of their use in cancer detection and treatment. *Curr Urol Rep* 2014;15(3):391.

25. Iremashvili V, Soloway MS, Pelaez L, et al. Comparative validation of nomograms predicting clinically insignificant prostate cancer. *Urology* 2013;81(6):1202-8.

26. Louie KS, Seigneurin A, Cathcart P, et al. Do prostate cancer risk models improve the predictive accuracy of PSA screening? A meta-analysis. *Ann Oncol* 2015;26(5):848-64.

27. Shariat SF, Kattan MW, Vickers AJ, et al. Critical review of prostate cancer predictive tools. *Future Oncol* 2009;5(10):1555-84.

28. Ross PL, Gerigk C, Gonen M, et al. Comparisons of nomograms and urologists' predictions in prostate cancer. *Semin Urol Oncol* 2002;20(2):82-8.

29. Walz J, Gallina A, Perrotte P, et al. Clinicians are poor raters of life-expectancy before radical prostatectomy or definitive radiotherapy for localized prostate cancer. *BJU Int* 2007;100(6):1254-8.

30. Ouzaid I, Yates DR, Hupertan V, et al. A direct comparison of the diagnostic accuracy of three prostate cancer nomograms designed to predict the likelihood of a positive initial transrectal biopsy. *Prostate* 2012;72(11):1200-6.

31. Eastham JA, May R, Robertson JL, et al. Development of a nomogram that predicts the probability of a positive prostate biopsy in men with an abnormal digital rectal

For peer review only

examination and a prostate-specific antigen between 0 and 4 ng/mL. *Urology* 1999;54(4):709-13.

32. Garzotto M, Hudson RG, Peters L, et al. Predictive modeling for the presence of prostate carcinoma using clinical, laboratory, and ultrasound parameters in patients with prostate specific antigen levels < or = 10 ng/mL. *Cancer* 2003;98(7):1417-22.

33. Karakiewicz PI, Benayoun S, Kattan MW, et al. Development and validation of a nomogram predicting the outcome of prostate biopsy based on patient age, digital rectal examination and serum prostate specific antigen. *J Urol* 2005;173(6):1930-4.

34. Kranse R, Roobol M, Schroder FH. A graphical device to represent the outcomes of a logistic regression analysis. *Prostate* 2008;68(15):1674-80.

35. Thompson IM, Ankerst DP, Chi C, et al. Assessing prostate cancer risk: results from the Prostate Cancer Prevention Trial. *J Natl Cancer Inst* 2006;98(8):529-34.

36. Brindle LA, Oliver SE, Dedman D, et al. Measuring the psychosocial impact of population-based prostate-specific antigen testing for prostate cancer in the UK. *BJU Int* 2006;98(4):777-82.

37. Moher D, Liberati A, Tetzlaff J, et al. Preferred reporting items for systematic reviews and meta-analyses: the PRISMA statement. *PLoS Med* 2009;6(7):e1000097.

38. Park SH, Goo JM, Jo CH. Receiver operating characteristic (ROC) curve: practical review for radiologists. *Korean J Radiol* 2004;5(1):11-8.

39. Han K, Song K, Choi BW. How to Develop, Validate, and Compare Clinical Prediction Models Involving Radiological Parameters: Study Design and Statistical Methods. *Korean J Radiol* 2016;17(3):339-50.

40. Steyerberg EW. Clinical prediction models: a practical approach to development, validation, and updating: Springer Science & Business Media 2008.

41. Pavlou M, Ambler G, Seaman SR, et al. How to develop a more accurate risk prediction model when there are few events. *BMJ* 2015;351:h3868.

42. Wolff RF, Moons KGM, Riley RD, et al. PROBAST: A Tool to Assess the Risk of Bias and Applicability of Prediction Model Studies. *Ann Intern Med* 2019;170(1):51-58.

43. Carlson GD, Calvanese CB, Partin AW. An algorithm combining age, total prostate-specific antigen (PSA), and percent free PSA to predict prostate cancer: results on 4298 cases. *Urology* 1998;52(3):455-61.

44. Babaian RJ, Fritsche H, Ayala A, et al. Performance of a neural network in detecting prostate cancer in the prostate-specific antigen reflex range of 2.5 to 4.0 ng/mL. *Urology* 2000;56(6):1000-6.

45. Jansen FH, van Schaik RH, Kurstjens J, et al. Prostate-specific antigen (PSA) isoform p2PSA in combination with total PSA and free PSA improves diagnostic accuracy in prostate cancer detection. *Eur Urol* 2010;57(6):921-7.

46. Hill OT, Mason TJ, Schwartz SW, et al. Improving prostate cancer detection in veterans through the development of a clinical decision rule for prostate biopsy. *BMC Urol* 2013;13:6.

For peer review only

47. Lazzeri M, Haese A, de la Taille A, et al. Serum isoform [-2]proPSA derivatives significantly improve prediction of prostate cancer at initial biopsy in a total PSA range of 2-10 ng/ml: a multicentric European study. *Eur Urol* 2013;63(6):986-94.

48. Porter CR, O'Donnell C, Crawford ED, et al. Predicting the outcome of prostate biopsy in a racially diverse population: a prospective study. *Urology* 2002;60(5):831-35.

49. Porter CR, Gamito EJ, Crawford ED, et al. Model to predict prostate biopsy outcome in large screening population with independent validation in referral setting. *Urology* 2005;65(5):937-41.

50. Catalona WJ, Bartsch G, Rittenhouse HG, et al. Serum pro-prostate specific antigen preferentially detects aggressive prostate cancers in men with 2 to 4 ng/ml prostate specific antigen. *J Urol* 2004;171(6 Pt 1):2239-44.

51. Sokoll LJ, Sanda MG, Feng Z, et al. A prospective, multicenter, National Cancer Institute Early Detection Research Network study of [-2]proPSA: improving prostate cancer detection and correlating with cancer aggressiveness. *Cancer Epidemiol Biomarkers Prev* 2010;19(5):1193-200.

52. Sokoll LJ, Wang Y, Feng Z, et al. [-2]proenzyme prostate specific antigen for prostate cancer detection: a national cancer institute early detection research network validation study. *J Urol* 2008;180(2):539-43; discussion 43.

53. Stephan C, Vincendeau S, Houlgatte A, et al. Multicenter evaluation of [-2]proprostate-specific antigen and the prostate health index for detecting prostate cancer. *Clin Chem* 2013;59(1):306-14.

54. Le BV, Griffin CR, Loeb S, et al. [-2]Proenzyme prostate specific antigen is more accurate than total and free prostate specific antigen in differentiating prostate cancer from benign disease in a prospective prostate cancer screening study. *J Urol* 2010;183(4):1355-9.

55. Loeb S, Sokoll LJ, Broyles DL, et al. Prospective multicenter evaluation of the Beckman Coulter Prostate Health Index using WHO calibration. *J Urol* 2013;189(5):1702-06.

56. Lughezzani G, Lazzeri M, Haese A, et al. Multicenter European external validation of a Prostate Health Index–based nomogram for predicting prostate cancer at extended biopsy. *Eur Urol* 2014;66(5):906-12.

57. Chun FK, Karakiewicz PI, Briganti A, et al. A critical appraisal of logistic regression-based nomograms, artificial neural networks, classification and regression-tree models, look-up tables and risk-group stratification models for prostate cancer. *BJU Int* 2007;99(4):794-800.

58. Herman MP, Dorsey P, John M, et al. Techniques and predictive models to improve prostate cancer detection. *Cancer* 2009;115(S13):3085-99.

59. Trottier G, Roobol MJ, Lawrentschuk N, et al. Comparison of risk calculators from the Prostate Cancer Prevention Trial and the European Randomized Study of Screening for Prostate Cancer in a contemporary Canadian cohort. *BJU Int* 2011;108(8 Pt 2):E237-44.

60. Collins GS, de Groot JA, Dutton S, et al. External validation of multivariable prediction models: a systematic review of methodological conduct and reporting. *BMC Med Res Methodol* 2014;14(1):40.

For peer review only

61. Miyake H, Kurahashi T, Takenaka A, et al. Improved accuracy for predicting the Gleason score of prostate cancer by increasing the number of transrectal biopsy cores. *Urol Int* 2007;79(4):302-6.

62. Chun FK, Briganti A, Graefen M, et al. Development and external validation of an extended 10-core biopsy nomogram. *Eur Urol* 2007;52(2):436-44.

63. Aus G, Abbou CC, Bolla M, et al. EAU guidelines on prostate cancer. *Eur Urol* 2005;48(4):546-51.

64. Vickers AJ, Ulmert D, Serio AM, et al. The predictive value of prostate cancer biomarkers depends on age and time to diagnosis: towards a biologically-based screening strategy. *Int J Cancer* 2007;121(10):2212-7.

65. Steuber T, O'Brien MF, Lilja H. Serum markers for prostate cancer: a rational approach to the literature. *Eur Urol* 2008;54(1):31-40.

66. Carlsson S, Assel M, Sjoberg D, et al. Influence of blood prostate specific antigen levels at age 60 on benefits and harms of prostate cancer screening: population based cohort study. *BMJ* 2014;348:g2296.

67. Cavadas V, Osorio L, Sabell F, et al. Prostate cancer prevention trial and European randomized study of screening for prostate cancer risk calculators: a performance comparison in a contemporary screened cohort. *Eur Urol* 2010;58(4):551-8.

68. Vickers AJ. Prediction models: revolutionary in principle, but do they do more good than harm. *J Clin Oncol* 2011;29(22):2951-52.

69. Shariat SF, Karakiewicz PI, Margulis V, et al. Inventory of prostate cancer predictive tools. *Curr Opin Urol* 2008;18(3):279-96.

70. Kawakami S, Numao N, Okubo Y, et al. Development, validation, and head-to-head comparison of logistic regression-based nomograms and artificial neural network models predicting prostate cancer on initial extended biopsy. *Eur Urol* 2008;54(3):601-11.

71. Hernandez DJ, Han M, Humphreys EB, et al. Predicting the outcome of prostate biopsy: comparison of a novel logistic regression-based model, the prostate cancer risk calculator, and prostate-specific antigen level alone. *BJU Int* 2009;103(5):609-14.

72. Moyer VA. Screening for prostate cancer: US Preventive Services Task Force recommendation statement. *Ann Intern Med* 2012;157(2):120-34.

73. Bell N, Connor Gorber S, Shane A, et al. Recommendations on screening for prostate cancer with the prostate-specific antigen test. *CMAJ* 2014;186(16):1225-34.

74. Lim LS, Sherin K, Committee APP. Screening for prostate cancer in US men: ACPM position statement on preventive practice. *Am J Prev Med* 2008;34(2):164-70.

75. Prostate Cancer UK. Consensus statements on PSA testing in asymptomatic men in the UK - information for health professionals 2016 [Available from: https://prostatecanceruk.org/about-us/projects-and-policies/consensus-on-psa-testing accessed 20-8 2019.

76. Frame I, Cant S. Current challenges in prostate cancer: an interview with Prostate Cancer UK. *BMC Med* 2015;13(1):166.

For peer review only

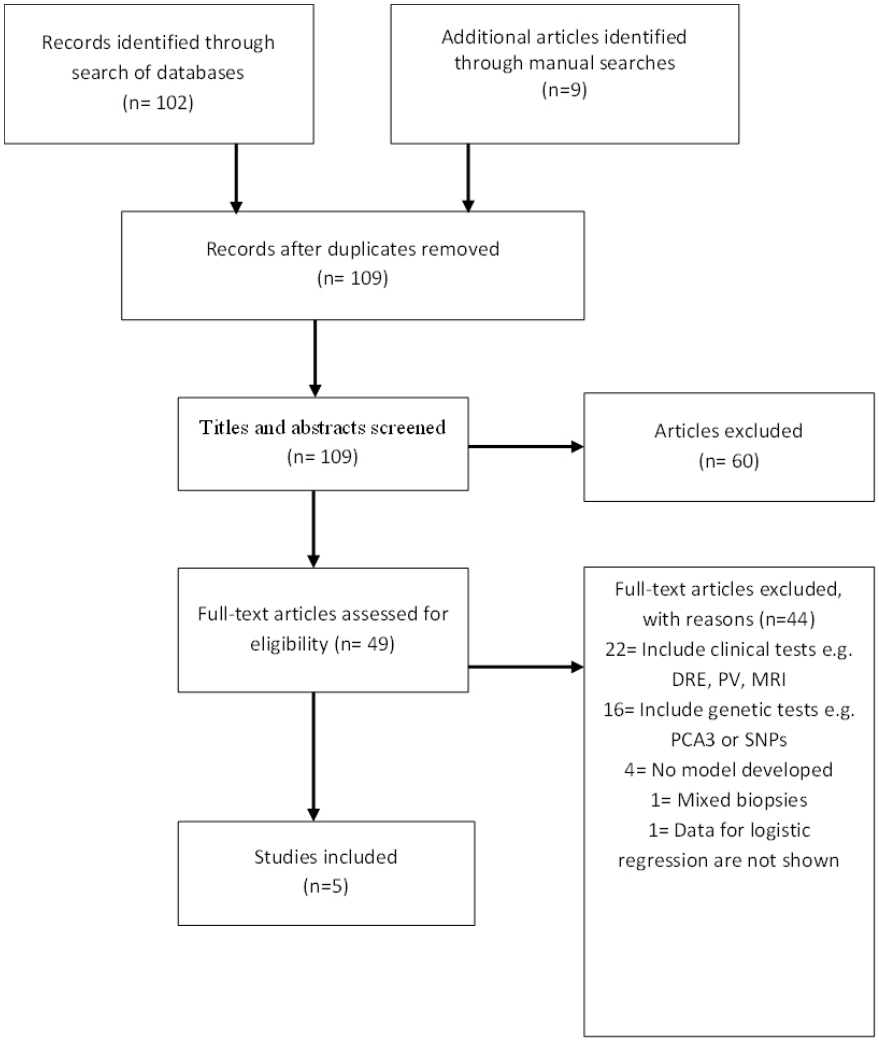

Flow diagram of studies included using PRISMA method

78x91mm (300 x 300 DPI)

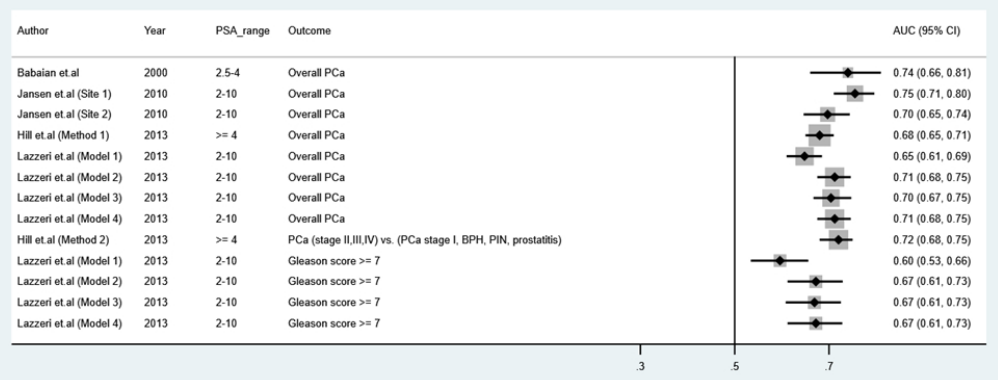

Model performance for each study using area under the curve (AUC)

81x33mm (300 x 300 DPI)

Reporting checklist for systematic review and meta-analysis.

Based on the PRISMA guidelines.

Instructions to authors

Complete this checklist by entering the page numbers from your manuscript where readers will find each of the items listed below.

Your article may not currently address all the items on the checklist. Please modify your text to include the missing information. If you are certain that an item does not apply, please write "n/a" and provide a short explanation.

Upload your completed checklist as an extra file when you submit to a journal.

In your methods section, say that you used the PRISMAreporting guidelines, and cite them as:

Moher D, Liberati A, Tetzlaff J, Altman DG, The PRISMA Group. Preferred Reporting Items for Systematic Reviews and Meta-Analyses: The PRISMA Statement

For peer review only

		Reporting Item	Page Number
**Title**			
	#1	Identify the report as a systematic review, meta-analysis, or both.	3
**Abstract**			
Structured summary	#2	Provide a structured summary including, as applicable: background; objectives; data sources; study eligibility criteria, participants, and interventions; study appraisal and synthesis methods; results; limitations; conclusions and implications of key findings; systematic review registration number	3-4
**Introduction**			
Rationale	#3	Describe the rationale for the review in the context of what is already known.	5-6
Objectives	#4	Provide an explicit statement of questions being addressed with	6

		reference to participants, interventions, comparisons, outcomes, and study design (PICOS).	
**Methods**			
Protocol and registration	#5	Indicate if a review protocol exists, if and where it can be accessed (e.g., Web address) and, if available, provide registration information including the registration number.	n/a
Eligibility criteria	#6	Specify study characteristics (e.g., PICOS, length of follow-up) and report characteristics (e.g., years considered, language, publication status) used as criteria for eligibility, giving rational	6
Information sources	#7	Describe all information sources in the search (e.g., databases with dates of coverage, contact with study authors to identify additional studies) and date last searched.	6
Search	#8	Present full electronic search strategy for at least one database, including any limits used, such that it could be repeated.	6
Study selection	#9	State the process for selecting studies (i.e., for screening, for determining eligibility, for inclusion in the systematic review, and, if applicable, for inclusion in the meta-analysis).	7
Data collection process	#10	Describe the method of data extraction from reports (e.g., piloted forms, independently by two reviewers) and any processes for obtaining and confirming data from investigators.	7
Data items	#11	List and define all variables for which data were sought (e.g., PICOS, funding sources), and any assumptions and simplifications made.	8
Risk of bias in individual studies	#12	Describe methods used for assessing risk of bias in individual studies (including specification of whether this was done at the study or outcome level, or both), and how this information is to be used in any data synthesis.	8
Summary measures	#13	State the principal summary measures (e.g., risk ratio, difference in means).	7
Planned methods of analyis	#14	Describe the methods of handling data and combining results of studies, if done, including measures of consistency (e.g., I2) for each meta-analysis.	6-8
Risk of bias	#15	Specify any assessment of risk of bias that may affect the	15-16

For peer review only

across studies		cumulative evidence (e.g., publication bias, selective reporting within studies).	
Additional analyses	#16	Describe methods of additional analyses (e.g., sensitivity or subgroup analyses, meta-regression), if done, indicating which were pre-specified.	n/a
**Results**			
Study selection	#17	Give numbers of studies screened, assessed for eligibility, and included in the review, with reasons for exclusions at each stage, ideally with a flow diagram.	8-9
Study characteristics	#18	For each study, present characteristics for which data were extracted (e.g., study size, PICOS, follow-up period) and provide the citation.	8-9
Risk of bias within studies	#19	Present data on risk of bias of each study and, if available, any outcome-level assessment (see Item 12).	15-17
Results of individual studies	#20	For all outcomes considered (benefits and harms), present, for each study: (a) simple summary data for each intervention group and (b) effect estimates and confidence intervals, ideally with a forest plot.	13-15
Synthesis of results	#21	Present the main results of the review. If meta-analyses are done, include for each, confidence intervals and measures of consistency.	12-15
Risk of bias across studies	#22	Present results of any assessment of risk of bias across studies (see Item 15).	15-17
Additional analysis	#23	Give results of additional analyses, if done (e.g., sensitivity or subgroup analyses, meta-regression [see Item 16]).	n/a
**Discussion**			
Summary of Evidence	#24	Summarize the main findings, including the strength of evidence for each main outcome; consider their relevance to key groups (e.g., health care providers, users, and policy makers	18-21
Limitations	#25	Discuss limitations at study and outcome level (e.g., risk of bias), and at review level (e.g., incomplete retrieval of identified research, reporting bias).	21-22

For peer review only

Conclusions	#26	Provide a general interpretation of the results in the context of other evidence, and implications for future research.	23

Funding

Funding	#27	Describe sources of funding or other support (e.g., supply of data) for the systematic review; role of funders for the systematic review.	24

The PRISMA checklist is distributed under the terms of the Creative Commons Attribution License CC-BY. This checklist was completed on 30. September 2019 using https://www.goodreports.org/, a tool made by the EQUATOR Network in collaboration with Penelope.ai

For peer review only

BMJ Open

Prediction models for prostate cancer to be used in the primary care setting: a systematic review.

Journal:	*BMJ Open*
Manuscript ID	bmjopen-2019-034661.R1
Article Type:	Original research
Date Submitted by the Author:	03-Mar-2020
Complete List of Authors:	Aladwani , Mohammad; The University of Manchester, Division of Population Health, Health Services Research and Primary Care School of Health Sciences Faculty of Biology, Medicine and Health<br>Lophatananon, Artitaya ; The University of Manchester, Division of Population Health, Health Services Research and Primary Care School of Health Sciences Faculty of Biology, Medicine and Health<br>Ollier, William; The University of Manchester, Division of Population Health, Health Services Research and Primary Care School of Health Sciences Faculty of Biology, Medicine and Health; Manchester Metropolitan University Faculty of Science and Engineering, School of Healthcare Science<br>Muir, Kenneth; The University of Manchester, Division of Population Health, Health Services Research and Primary Care School of Health Sciences Faculty of Biology, Medicine and Health
**Primary Subject Heading**:	Public health
Secondary Subject Heading:	Urology, Epidemiology
Keywords:	EPIDEMIOLOGY, UROLOGY, Prostate disease < UROLOGY

SCHOLARONE™
Manuscripts

I, the Submitting Author has the right to grant and does grant on behalf of all authors of the Work (as defined in the below author licence), an exclusive licence and/or a non-exclusive licence for contributions from authors who are: i) UK Crown employees; ii) where BMJ has agreed a CC-BY licence shall apply, and/or iii) in accordance with the terms applicable for US Federal Government officers or employees acting as part of their official duties; on a worldwide, perpetual, irrevocable, royalty-free basis to BMJ Publishing Group Ltd ("BMJ") its licensees and where the relevant Journal is co-owned by BMJ to the co-owners of the Journal, to publish the Work in this journal and any other BMJ products and to exploit all rights, as set out in our licence.

The Submitting Author accepts and understands that any supply made under these terms is made by BMJ to the Submitting Author unless you are acting as an employee on behalf of your employer or a postgraduate student of an affiliated institution which is paying any applicable article publishing charge ("APC") for Open Access articles. Where the Submitting Author wishes to make the Work available on an Open Access basis (and intends to pay the relevant APC), the terms of reuse of such Open Access shall be governed by a Creative Commons licence – details of these licences and which Creative Commons licence will apply to this Work are set out in our licence referred to above.

Other than as permitted in any relevant BMJ Author's Self Archiving Policies, I confirm this Work has not been accepted for publication elsewhere, is not being considered for publication elsewhere and does not duplicate material already published. I confirm all authors consent to publication of this Work and authorise the granting of this licence.

For peer review only

Full title: Prediction models for prostate cancer to be used in the primary care setting: a systematic review.

Institutional addresses and e-mail addresses of the contributing authors:

Dr Mohammad Aladwani

Division of Population Health, Health Services Research and Primary Care, School of Health Sciences Faculty of Biology, Medicine and Health, The University of Manchester, Manchester, M13 9PT, UK

E-mail: mohammad.al-adwani@postgrad.manchester.ac.uk

Dr Artitaya Lophatananon

Division of Population Health, Health Services Research and Primary Care, School of Health Sciences Faculty of Biology, Medicine and Health, The University of Manchester, Manchester, M13 9PT,UK

E-mail: artitaya.lophatananon@.manchester.ac.uk

Professor William Ollier,

Division of Population Health, Health Services Research and Primary Care, School of Health Sciences Faculty of Biology, Medicine and Health, The University of Manchester, Manchester, M13 9PT, UK

School of Healthcare Science, Faculty of Science and Engineering, Manchester Metropolitan University, John Dalton Building, Manchester, M1 5GD, UK

E-mail: Bill.Oliier@manchester.ac.uk

Address and contact details of the corresponding author:

Professor Kenneth R. Muir

Division of Population Health, Health Services Research and Primary Care,

School of Health Sciences, Faculty of Biology, Medicine and Health,

The University of Manchester, Manchester, M13 9PT, UK

E-mail: kenneth.muir@manchester.ac.uk

For peer review only

Telephone: +44-161-275-5518

Key words: Prostate cancer, prostate specific antigen, PSA, prostate cancer screening, risk Prediction, risk tools, prediction models, primary care, community.

For peer review only

Abstract

Objective

To identify risk prediction models for prostate cancer (PCa) that can be used in the primary care and community health settings.

Design

Systematic review.

Data sources

MEDLINE and EMBASE databases combined from inception and up to the end of January 2019.

Eligibility

Studies were included based on satisfying all the following criteria; (i) presenting an evaluation of PCa risk at initial biopsy in patients with no history of PCa, (ii) studies not incorporating an invasive clinical assessment or expensive biomarker/ genetic tests, (iii) inclusion of at least two variables with PSA being one of them, and (iv) studies reporting a measure of predictive performance. The quality of the studies and risk of bias was assessed by using the PROBAST tool.

Data extraction and synthesis

Relevant information extracted for each model included; the year of publication, source of data, type of model, number of subjects, country, age, PSA range, mean/median PSA, other variables included in the model, number of biopsy cores to assess outcomes, study endpoint (s), cancer detection, model validation, and model performance.

Results

An initial search yielded 109 potential studies, of which five met the set criteria. Four studies were cohort-based and one was a case-control study. PCa detection rate was between 20.6% and 55.8%. AUC was reported in four studies and ranged from 0.65 to 0.75. All models showed significant improvement in predicting PCa compared with

For peer review only

being based on PSA alone. The difference in AUC between extended models and PSA alone was between 0.06 and 0.21.

Conclusions

Only a few PCa risk prediction models have the potential to be readily used in the primary health care or community health setting. Further studies are needed to investigate other potential variables that could be integrated into models to improve their clinical utility for PCa testing in a community setting.

For peer review only

Strengths and limitations

- The review focussed on risk prediction models for PCa for use in primary care.
- The PRISMA approach was followed in identifying relevant articles and reporting this study.
- We used the PROBAST tool to assess the quality and risk of bias in the included models.
- The search strategy was restricted to two databases and a manual search, to retrieve original studies.

INTRODUCTION

Prostate cancer (PCa) is the second most common cancer and the fifth leading cause of cancer-attributed death in men worldwide with an estimated incidence of 1,276,106 and 358,989 deaths in 2018 [1]. In the UK, around 47,200 new cases of PCa were reported in 2015, accounting for 26% of all new cancer cases in males. PCa deaths in the UK of were around 11,600 in 2016 [2]. The global projections of PCa incidence and mortality for 2030 are 1.7 and 0.5 million, respectively [3]. The highest incidence of PCa is seen in western societies [4]. The significant increase of PCa incidence and diagnosis over the last three decades can be attributed mainly to the widespread implementation of the PSA serum test after it had been introduced in the late 1980s [5 6].

The strong association of PSA with PCa [7 8], along with it being a relatively inexpensive test [9], has made PSA a key biomarker in the diagnostic processs of PCa and for the recommendation of a confirmatory prostate biopsy [7 9]. PSA is, however, not a cancer-specific marker [5 10]. Conditions such as benign prostate hypertrophy (BPH), prostatitis, and other non-malignant prostatic conditions can also elevate PSA level, thus introducing uncertainty to the application of the test [11-14]. This highlights limitations of the PSA test regarding its specificity and sensitivity and it being largely dependent on setting a ‘diagnostic’ cut-off point, which often leads to an unacceptable number of false-positive and false-negative results [5 10 15 16]. Such issues are likely to be the part of the explanation for the significant number of unnecessary biopsies currently performed each year. Such procedures are associated with adverse side effects for patients and also increases health care costs [17 18].

For peer review only

To address such PSA test limitations, researchers have incorporated other measurable factors into approaches for the early detection of PCa; these “risk assessment tools” are based on statistical models designed to improve the accuracy and performance of the PSA test [19-22]. Logistic regression and artificial neural network (ANNs) models are now considered to be the most common and effective statistical techniques in aiding development of new models to enhance early PCa diagnosis [23]. These PCa risk prediction models can be used to aid the testing of men for further investigations.

Currently in the UK, there is no population based screening programme for PCa. The ultimate goal of PCa screening is to find intermediate and high risk of PCa rather than low risk PCa that would not require treatment but will give emotional burden to the patient once detected and unnecessary treatment in some patients. An important potential advantage of the extended risk models is their ability to provide a more accurate estimation of PCa risk. This may ultimately lead to their use in patient counselling and decision-making [24-27]. Such models have already achieved better results in predicting probabilities of outcome compare to clinical judgment [28 29]. Furthermore, it has been reported that using such predictive models may minimize the rate of unnecessary biopsies [30].

Recently there has been a substantial increase in the development of predictive models to help clinicians assess risk and decide which man to send to clinical setting to further investigate for a possible diagnosis of PCa [22 26 30-35]. The majority of these models are designed for use in clinical settings, where costs are less of an issue and most include the need for a clinical examination such as digital rectal examination (DRE) or trans-rectal ultrasonography (TRUS). One of the main limitations of DRE is that it has poor performance, especially at low PSA levels, and it is highly subjective to inter-observer variability [36-38]. A meta-analysis study revealed that DRE has positive predictive value of only 18% [39]. Similarly, TRUS has been reported for having poor accuracy at low PSA levels [40 41] and small PCa might not be palpable on DRE or visualisation on TRUS [40]. Furthermore, less than 40% of PCa detected by DRE are potentially curable, making it less beneficial for early diagnosis [42]. Several studies showed that there are fear, anxiety and embarrassment among some men, in particular black men, regarding the DRE test [43-46]. Another disadvantage of the DRE is the fact it is a potentially uncomfortable test [47-

For peer review only

[52]. This may explain why the DRE is a barrier for some men to participate in PCa screening if it is including DRE test. Lee et al reported that 74%-84% of black men may not maintain annual DRE screening [53], while another study found that it may prevent 22% of men to participate [54]. Since TRUS need to be performed by a skilled urologist, this means men have to make an appointment with a clinic in different location, which makes the screening less convenience. As a result, men may feel reluctant to have such tests performed.

This systematic review of the literature was undertaken to identify risk prediction models that do not incorporate invasive or more costly clinical procedures or extensive biomarkers but have potential application for use in primary care and community settings. As low cost is a primary concern for community use, for this review we set an indicative threshold of approximately 3-5 times the cost of a PSA test for inclusion. This excluded a number of models that contain new and emerging biomarker or SNP panels. As the numbers of person referred to clinical setting costs are less of an issue. The performance of the models reviewed for detecting PCa at initial biopsy have been compared using 'reported area under the curve' (AUC) and/or sensitivity - specificity testing.

For peer review only

METHODS

The approach used to identify and select relevant articles was based on the application of the '*Preferred Reporting Items for Systematic Reviews and Meta-Analysis'* (PRISMA) [55].

Data sources and search strategy

A literature search was performed using Medline (via OVID) and Embase databases. The 'medical subject heading' (MeSH) terms, combined with Boolean logic operators 'AND' and 'OR', were applied to retrieve relevant articles. The terms used for the search were "Prostatic Neoplasms" AND ("Initial biopsy" OR "first biopsy" OR "early detection of cancer") AND ("nomograms" OR "artificial neural networks" OR "risk assessment" OR "statistical model"). The full search strategy is provided in a supplementary file (S1). All articles defined (published since the inception of the databases and up to the end of January 2019) were subsequently further filtered as being those only published in

English language and with an abstract. Further to using the above search databases, the research articles were selected manually from the reference lists of any relevant review articles. Google Scholar and Medline searches were also carried out to identify independent study for external validation for each model included in this review. The results are presented in supplementary table (S2).

Eligibility criteria

As this review focuses on PCa risk prediction based in community healthcare settings, all studies were selected on the following inclusion criteria (i) evaluating the risk for PCa at initial biopsy in subjects who had no prior history of PCa (ii) studies that reported "low cost" risk assessment tools (i.e. those not including more expensive genetic, biomarker test) or "invasive" clinical tests/examinations (such as DRE or TRUS) (iii) studies that included a minimum of two variables of which PSA had to be one of them [on the basis that an elevated PSA test in UK primary care is usually the first sign and rationale for suggesting a need for further investigation of PCa within NICE guidelines] and (iv) studies that reported AUC and/or sensitivity and specificity of the diagnostic/predictive tool. The exclusion criteria used were (i) articles with models that were built and based on repeat or mixed biopsies (ii) studies that only validate an existing model (iii) articles that were not published in English. There were no time boundaries regarding the publication year.

Screening of the titles, abstracts, and full-texts was carried out by two reviewers (MA, AL). Any concerns about the eligibility of a study were resolved by discussion with a third reviewer (KM).

Data extraction

A data extraction form was developed to collect all relevant information. For each study used in this review, the items extracted included; year of publication, source of data, type of model, number of subjects, country where it was performed, age, PSA range, mean/median PSA, number of biopsy cores, variables included in the model, study endpoint(s), cancer detection, model performance, and model validation.

For peer review only

Evaluating the performance of the risk models

Prediction models can be evaluated against various criteria. The most critical measurements of model performance are discrimination and calibration [27]. Discrimination refers to how well the prediction model can differentiate subjects in different outcome classes according to their predicted risks. It is often assessed by measuring the area under the receiver operating characteristic (ROC) curve [56]. It also requires setting a series of thresholds to separate low and high ranges of predicted outcomes. A value of 0.5 indicates no discrimination, while a value of 1 indicates perfect discrimination. However, even with perfect discrimination, observed risk can differ from expected risk. Therefore, calibration has an important role in model evaluation [57]. Calibration represents the agreement between expected and observed outcomes [58]. A well-calibrated model is achieved when the calibration slope is close to 1. When the calibration slope is less than 1 it indicates that the model under-estimates low risks and over-estimate high risks [59].

Due to the heterogeneity of the studies included, conducting a meta-analysis was not applicable.

Study quality assessment

The quality of the studies included in this review was assessed using the PROBAST tool [60]. This tool has been developed specifically to assess the risk of bias and applicability for prediction model studies. The tool consists of four domains and has 20 signalling questions that facilitate to reach overall judgment of risk of bias, as well as issues relating to applicability.

Patient and Public involvement

No patients were involved in setting the research question or the outcome measures, nor were they involved in the design and implementation of the study. There are no plans to involve patients in dissemination.

RESULTS

A total of 102 publications were identified using the search strategy as shown in Fig.1. An additional nine articles were identified through manual searches from a bibliography

For peer review only

of reviewed articles. At the first filter step, a total of 109 titles and abstracts were screened for eligibility after removing two duplicates. In the second filter step, 60 papers failed to meet the inclusion criteria and were excluded, resulting in 49 articles. The final step of filtering yielded only five studies that were considered to be eligible (i.e. passed all set criteria) and were thus included in this systematic review. There is no independent study identified for external validation for included models.

Study characteristics

Four of the five studies included were based on cohort studies and one was a case-control study. The characteristics of each of these studies and populations are summarised in Table 1. Details of PSA assays used in the models are presented in supplementary table (S3).

For peer review only

Table 1: Characteristics of the included studies.

Author & year	Type of model	Type of study	Sample No.	Location	Population type	Age	Median Age	PSA range	Median PSA	No. of biopsy cores	Cancer detection
Carlson, 1998 [61]	Logistic regression	Cohort	Model dev = 3773<br>Validation = 525	Baltimore, USA	Referral	≥ 45	—	4-20 ng/ml	—	Sextant biopsy	32%
Babaian, 2000 [62]	Neural network = 3 ANNs	Cohort	151	Texas, USA	Screening program	40-75	62	2.5-4 ng/ml	—	11 cores	24.5%
Jansen, 2010 [63]	Logistic regression	Cohort	Site 1 = 405<br>Site 2 = 351	Site 1 from the Rotterdam arm of the European Study of screening for Prostate cancer<br>Site 2 Innsbruck, Austria	Screening program	≥ 50	Site 1 (66)<br>Site 2 (60)	2-10 ng/ml	~ 4.4	≥6 cores	Site 1 = 55.8%<br>Site 2 = 49.6%
Hill, 2013 [64]	Logistic regression	Case-control	1378	Florida, USA	Hospital referral	40-90	—	≥ 4 ng/ml	—	N/A	20.6%
Lazzeri, 2013 [65]	Logistic regression	Cohort	646	European multi-centre; Italy, Germany, France, Spain, and the UK	Referral	>45	—	2-10 ng/ml	~ 5.8	≥ 12 cores	40.1%

Subjects used to build the risk models varied across these studies. Of the five studies, three studies included men from referral populations [61 64 65] and two studies from screening programs [62 63]. The sample sizes ranged from 151 to 3,773 with three studies derived from US cohorts [61 62 64] and two from Europe [63 65]. Four studies used logistic regression methodology to build their model, whereas one study used an artificial neural network-based approach (ANN) [62]. The minimum age of participants was 40 years old [62 64] and the minimum PSA level was 2 ng/ml [63 65].

Variables in the model

Table 2 presents details of the variables used in each model. PSA level was used in all models, followed by free PSA (fPSA), age, and free-to-total PSA ratio (%fPSA). Other variables also reported in the models included; precursor of prostate-specific antigen (p2PSA), percentage of p2PSA to free PSA (%p2PSA), prostate health index (PHI), levels of haemoglobin, albumin, creatinine and red blood cell count (RBC), haematuria, mean corpuscular volume (MCV), and prostatic acid phosphatase.

For peer review only

Table 2: Variables used in the prostate cancer risk prediction models*.

Author & year	Variables used in the model				
	Total PSA	Free PSA	Percent free PSA	Age	Other variables
Carlson, 1998 [61]	✓		✓	✓	
Babaian, 2000 [62]	✓	✓		✓	Creatinine Kinase, Prostatic acid phosphatase
Jansen, 2010 [63] *(Site 1)*	✓	✓			p2PSA
Jansen, 2010 [63] *(Site 2)*	✓	✓			p2PSA
Hill, 2013 [64] *(Method 1)*	✓			✓	HGB, RBC, Haematuria, Creatinine, MCV, and ethnicity "Black"
Hill, 2013 [64] *(Method 2)*	✓			✓	HGB, RBC, Creatinine, and MCV
Lazzeri, 2013 [65] *(Model 1)*	✓	✓	✓		
Lazzeri, 2013 [65] *(Model 2)*	✓	✓	✓		p2PSA
Lazzeri, 2013 [65] *(Model 3)*	✓	✓	✓		%p2PSA
Lazzeri, 2013 [65] *(Model 4)*	✓	✓	✓		PHI

*HGB= Haemoglobin. RBC= Red blood cells. MCV= Mean corpuscular volume. PHI= Prostate Health Index. p2PSA= Precursor of prostate specific antigen, %p2PSA= p2PSA/fPSA

For peer review only

Outcome

The study endpoint also varied among the studies selected. Two studies evaluated the accuracy of detecting any PCa [61 62] and three studies examined the pathologic Gleason score [63-65]. Although Jansen et al, did not build a model to predict the aggressiveness of prostate cancer, they assessed the relationship of each variable individually with a Gleason score ≥7. PCa was determined by taking a needle biopsy. All subjects in the five studies underwent prostate biopsy. The least number of biopsy cores used were 6 [61 63] and the highest were ≥ 12 [65]. One study did not report the number of biopsy cores taken [64]. PCa rates ranged from 20.6% to 55.8%.

Evaluating the performance of the risk models

For predicting any PCa, the Jansen et al, model used data from the Rotterdam arm of the European Study of Screening for PCa (ESPRC). Their model achieved the highest discrimination value when compared to PSA alone (AUC of 0.755 vs. 0.585, respectively) [63]. The AUC values in other studies ranged from 0.648 to 0.74.

One study did not provide the AUC but instead reported an increase of 11% in specificity over percent free PSA alone with 95% sensitivity [61]. Lazzeri et al, [65] presented results from four separated models discriminating PCa with a Gleason score of ≥7. Lazzeri's model 2 (which includes the base model tPSA, fPSA and %fPSA in addition to p2PSA) and model 3 (which includes base model plus PHI) showed the highest levels of discrimination out of the 4 models with an AUC of 0.67. In the study of Hill [64] the authors classified PCa stages differently and built their two models accordingly. In Hill's first model, the difference in the discrimination was analysed and based on all PCa versus non-cancerous prostate conditions where the AUC for this model was 0.68 compared to 0.59 for PSA alone. In Hill's second model, the discrimination analysis was based on PCa stages II, III, IV versus PCa stage I, prostatic interstitial neoplasm (PIN), BPH and prostatitis where stages I, II, III and IV are parallel to T1, T2, T3/T4, and metastatic PCa respectively. The AUC for the second model was 0.72 compared to 0.63 for PSA alone. In general, four studies examined the AUC with PSA alone and all reported a benefit from the use of logistic regression or the trained

For peer review only

ANN. Model performance and the differences between the AUC's for PSA alone and for the extended models are presented in Table 3.

Table 3: The difference of area under the curve (AUC) for PSA alone and extended model.

Study	AUC for PSA	AUC for model	ΔAUC (Model – PSA)
Carlson [61]	NA	NA	NA
Babaian [62]	0.64 %fPSA	0.74	0.1
Jansen [63] (*site 1)*	0.58	0.75	0.17
Jansen [63] *(site 2)*	0.53	0.7	0.16
Hill [64] *(method 1)*	0.59	0.68	0.09
Hill [64] *(method 2)*	0.63	0.72	0.09
Lazzeri [65]	0.50 for any PC<br><br>0.54 for Gleason score ≥7	Model 1 = 0.65<br>Model 1 (Gleason score≥7) = 0.60	0.15<br>0.06
		Model 2= 0.71<br>Model 2 (Gleason score≥7) = 0.67	0.21<br>0.13
		Model 3= 0.704<br>Model 3 (Gleason score≥7) = 0.67	0.2<br>0.13
		Model 4= 0.71<br>Model 4 (Gleason score≥7) = 0.672	0.21<br>0.13

Sensitivity and specificity data are presented in Table 4. At 95% sensitivity, the Babaian et al, model shows the highest specificity (51%) whereas the Jansen model for both sites had the lowest specificity (~23.5%). In the Hill study, with a sensitivity of ~90%, the specificity was lower than in other studies (~18% and 28%) for method 1 and 2 respectively. In the study reported by Lazzeri, the sensitivity and specificity were not reported for the overall model; instead their study reports sensitivity and specificity for predictive variables individually. The highest sensitivity (90.5%) of %p2PSA and %fPSA achieved the highest specificity in predicting PCa at 21.5% and 22.8% respectively. Percentage p2PSA and PHI were more associated with Gleason scores.

For peer review only

Table 4: Sensitivity and specificity profile at different levels for each model.

Study	Sensitivity	Specificity	Probability cut-off	Positive predictive value	Negative predictive value
Carlson [61]	99	18	>15	≤47	N/A
	95	34	18	51	NA
	89	43	20	42	NA
Babaian [62]	95	51	NA	39	97
	92	62	NA	44	96
	89	62	NA	43	95
Jansen [63] *(Site 1)*	95	23.9	NA	NA	NA
Jansen [63] *(Site 1)*	90	30.1	NA	NA	NA
Jansen [63] *(Site 2)*	95	23.2	NA	NA	NA
Jansen [63] *(Site 2)*	90	36.2	NA	NA	NA
Hill [64] *(Method 1)*	90.9	17.6	33	47.1	70.5
Hill [64] *(Method 2)*	89.8	28	13	20.6	91.3
Hill [64] *(Method 1)*	80.5	37.1	37	50.9	70.2
Hill [64] *(Method 2)*	78.2	45	15	28.7	88.8
Hill [64] *(Method 1)*	39.9	81.4	48	63.4	62.6
Hill [64] *(Method 2)*	45.8	79.5	23	36.7	85
* Lazzeri [65] model reported only sensitivity and specificity for predictive variables individually and at sensitivity of 90, %p2PSA and %fPSA achieved the highest specificity					

For peer review only

Table 5 summarises the validation and calibration results for the studies included. Model calibration was reported in two studies [61 65]. Carlson plotted the observed and expected risks using calibration plots, whereas Lazzeri used the Hosmer-Lemeshow goodness-of-fit test. In terms of validation, two studies did not report model validation [63 64]. Only one study reported an external validation using an additional data set consisting of 525 patients [61]. Cross-validation using multiple re-sampling schemes was used in the

Babaian Study, however, they did not report the number of time this was performed [62]. Lazzeri used 200 bootstrap re-samples to minimise overfitting bias [65].

Table 5: Validation and calibration for included models.

Author & Year	Validation	Calibration
Carlson,1998 [61]	External validation on additional data set consisting of 525 patients	Calibration plot
Babaian,2000 [62]	Cross-validation and separate data set of 151	N/A
Jansen, 2010 [63]	N/A	N/A
Hill, 2013 [64]	N/A	N/A
Lazzeri, 2013 [65]	Internal validation using 200 bootstrap resamples	Internal calibration using the Hosmer-Lemeshow goodness-of-fit test

Study quality assessment

Quality assessment was carried out by two reviewers (MA and AL) with any discordance resolved by a third reviewer (KM). The assessment of results suggested some issue of study quality according to the criteria as set in the PROBAST tool, particularly in the analysis domain. For instance, one study applied univariable analysis to select predictors [61]. Three studies did not measure calibration [62-64]. Furthermore, two studies did not account for optimism and overfitting by using internal validation methods [63 64]. Whereas one study did not use appropriate measures for model performance i.e. AUC, this study reported the calibration [61].

The event per variable (EPV) was lower than recommended (< 10) [59 66] in the Babaian [62] study indicating inadequate power. Four studies did not report missing data or how they handled it [61-63 65]. The remaining study used complete-case analysis and excluded subjects with missing data on laboratory biomarkers (n=75) [64]. The PROBAST

guidelines state that in a prediction model study where any risk of bias and applicability is low in all four domains, a regrading to high risk of bias should be considered when the study did not validate the model externally [60]. Thus, although the quality assessment for the Lazzeri study [65] was graded low risk in all the four domains, since the study did not report any external validation of the model, the assessment of the study has been regraded to high risk of bias according to the PROBAST criteria. A full quality assessment for all studies is presented in Table 6.

For peer review only

Table 6: Quality assessment for risk of bias and applicability concern for included studies.

Study	ROB*				Applicability			Overall	
	Participants	Predictors	Outcome	Analysis	Participants	Predictors	Outcome	ROB	Applicability
Carlson [61]	+	+	-	-	+	+	-	-	-
Babaian [62]	+	+	+	-	-	+	+	-	-
Jansen [63]	-	-	-	-	+	-	-	-	-
Hill [64]	-	+	+	-	-	+	+	-	-
Lazzeri [65]	+	+	+	+	+	+	+	-	+

* ROB- Risk of bias
+ indicates a low risk of bias or applicability; - indicates a high risk of bias or applicability.

DISCUSSION

Despite the large number of PCa risk prediction models, the majority still include clinical inputs and/or more costly biomarker or genetic panels; few low cost models exist that do not include specialist clinical input or more expensive further testing that limits there use for population wide assessments. To our knowledge, this is the first study to examine risk prediction models for PCa that are low cost and do not include clinical and genetic variables and are based on single time point assessment.

Our study identified five unique models that met the set criteria. The Carlson model [61] has the largest population (3,773 subjects) when compared to the other four studies. Although they reported an 11% increase in specificity, they did not report AUC predictive estimates. It has been acknowledged that sensitivity and specificity results are dependent on the prevalence of the disease. Hence the comparison between populations where the PCa prevalence may vary (especially in early detection) will be difficult [33]. More importantly, by not reporting the AUC estimate, the model raises some doubts regarding the reliability of the model and its implementation [33]. It will also make comparison to other models not applicable [67].

Babaian [62] developed an algorithm and compared the performance of the ANN to PSA density (total PSA divided by prostate volume) (PSAD), %fPSA, and transition zone density (PSAD-TZ). Their ANN demonstrated a significant increase of model specificity that reached 51% when sensitivity was held at 95%. This was better than the specificity value of each individual variable such as %fPSA (10%), PSAD (39%), and PSAD-TZ (22%). In terms of AUC, the ANN achieved a moderate accuracy (0.74) being the second highest among all studies included. However, the ANN model did not show significant improvement when compared with a model fitted with only individual variables (AUC for %fPSA= 0.64, PSAD= 0.74, PSAD-TZ= 0.75). They included a number of uncommon pre-biopsy inputs into their algorithm such as prostatic acid phosphatase and creatinine kinase [68]. Furthermore, they used a tight PSA range (2.5-4.0ng/ml) which meant that their model may be less suitable for patients with PSA level below or above that range, thus limiting its generalisability.

For peer review only

The study by Jansen and colleagues [63] demonstrated that adding p2PSA to the base model of PSA and fPSA significantly enhanced the PCa predictive value and specificity. The association and added value of p2PSA in the prediction and detection of PCa have been reported by several other studies [16 69-72]. Jansen [63] showed that p2PSA has no clear association with aggressive PCa. However, the base model that includes p2PSA had the highest clinical significance in correlation to pathologic Gleason score with a p-value of 0.008 compared to %fPSA and PHI (p-value 0.01 and 0.02) respectively. Although they used archived blood samples and retrospective analysis, the results were similar to a prospective study of 268 patients [16].

Hill [64] used a case-control study to evaluate several laboratory biomarkers. They found HGB, RBC, haematuria, creatinine, PSA, Age, MCV, and ethnicity ("being black") were statistically significantly associated in the first method ($p < 0.05$). In the second method, HGB, RBC, creatinine, PSA, age, and MCV were found to be statistically significantly correlated ($p < 0.001$) with PCa. However, since this study was designed as a case-control study, it would have been more prone to uncontrolled confounding and selection bias. Moreover, the type of screening protocols used in Veterans' Administrations may vary to those conducted in other healthcare systems; therefore, the results may not be applicable to other populations. Furthermore, subjects with a PSA level < 4.0 ng/ml have not been investigated, and thus, the performance of the models are unknown for individuals in this group.

Lazzeri et al, [65] in a European multi-centre study have evaluated similar biomarkers as in Jansen study with the same PSA range 2-10 ng/ml prospectively. They found no difference in both %p2PSA and PHI as individual PCa predictors with AUC of 0.67 (95% CI 0.64-0.71). However, the base model (consisting of PSA, fPSA, and %fPSA) that also included either p2PSA or PHI out-performed the base model alone and the base model including %p2PSA. In the analysis, the additive value of both p2PSA and PHI is 0.064 and 0.056 for %p2PSA for predicting the risk of PCa. These additive values increased to 0.076 for both p2PSA and PHI and 0.073 for %p2PSA in predicting Gleason scores ≥7 for the disease. The usefulness of PHI in improving the predictive accuracy of PCa over total and free PSA has been confirmed and reported by several studies [16 72-75].

For peer review only

In general, only one study has validated their model externally [61], whereas the remaining studies were either validated internally [62 65] or not reported any validation methods [63 64]. Prediction models may not be equally applicable to all data sets as patients' characteristics may vary [20 76]. As a result, the generalisability of a model might be poor when it used in populations other than that used in building the model. Therefore, external validation should be conducted before applying any new model into general practice [77 78].

Another key performance measure of any model that needs careful evaluation is calibration. A calibration plot with a calibration slope is more preferable than the Hosmer-Lemeshow test; it has been acknowledged that evaluating a good and well calibrated model based on a large dataset can still fail the Hosmer-Lemeshow test. In contrast, when evaluating a poorly calibrated model with a small dataset it can still pass the Hosmer-Lemeshow test [79]. In our analysis, three studies fail to report the calibration of the model [62-64] whilst the Carlson study [61] used a calibration plot and Lazzeri [65] used the Hosmer-Lemeshow test. Excluding calibration from the majority of models may explain why some models are not currently used in practice [79].

With regard to biopsy cores, only two studies used extended biopsy cores. Babaian [62] used an 11-core multi-site biopsy, whereas Lazzeri [65] used at least 12 biopsy cores. Moreover, two studies used six cores biopsy in their model [61 63]. The use of six-core biopsy has been criticized as not being adequate in detecting PCa [80] and that models developed using sextant biopsy are less accurate than when a 10-core biopsy is used [81]. As a result, the European guideline for clinical PCa recommended an extended biopsy as standard practice for PCa detection [82].

It is worth noting that all five reviewed models performed better than just PSA alone. However, none of them has both high specificity and sensitivity. The level of sensitivity has been increased, and despite enhancement in the specificity, it is still considered low. Specificity is crucial when it comes to being used in a population setting as men without PCa should be ruled out as much as possible from further invasive engagement with the health system.

For peer review only

Our review therefore suggests that none of the reviewed models provide an ideal performance in predicting PCa with high sensitivity and high specificity. It is particularly important when considering the application of PCa risk prediction at the population level that the tool used should be able to both detect the outcome and filter out people with no disease. As there is robust evidence suggesting that the clinical relevance of PSA range to the detection of PCa differs across age groups [83-85], any future model should consider PSA threshold in relation to a specific age range. Risk prediction models for PCa should therefore take account of age.

Comparison with other studies

To our knowledge, three systematic reviews of PCa prediction tools have been published [20 26 27]. In the Louie et al. review, risk models were included that were externally validated in at least five study populations for the purpose of meta-analysis and only six studies were included in their analysis. Furthermore, all the studies included incorporated clinical tests such as DRE and/or TRUS-PV [26]. Schroder and Kattan [20] reviewed models that were built to predict the likelihood of having a positive prostate biopsy for cancer. However, it appears that they also included models where subjects had a previous negative biopsy. As such, some of the models included variables related to biopsy results and cores. The review by Shariat and colleagues examined different types of predictive tools [27]. They explored tools that predict PCa on initial and repeat biopsy, pathologic stages, biochemical recurrence after radical proctectomy, metastasis, survival, and life expectancy. Similarly, virtually all of the prediction tools that were based on initial biopsy included variables based on invasive procedures.

Strengths and limitations of this study

This report is the first to review risk prediction tools for PCa that can be used in primary care and community settings. Any prediction model should therefore be simple to use, based on non-invasive tests, be feasible at a population level and at low cost. We carried out an extensive data extraction relating to important features and characteristics for each study included, such as modelling method, source of data, sample size, variables, discrimination, validation, and cancer detection rate. We have

For peer review only

also followed PRISMA guidelines for identifying eligible articles as well as for reporting this study. In addition, the PROBAST tool was adopted to assess the quality and risk of bias for each prediction model.

Our study has some limitations. Our aim was to identify prediction models that have the potential to be implemented in a primary care or community settings, and consequently our search strategy was to retrieve relevant studies for this specific purpose. Furthermore, we excluded articles that were not published in English or did not have an abstract. Moreover, only two databases were searched, besides manual search, to retrieve original studies.

A previous systematic review suggested that the majority of relevant studies could be identified through a manual search of articles reference lists instead of a database alone [20]. We identified four eligible studies using this approach. Given the small number of models identified by the approach we followed, that can be applied in primary care settings compared to the large number relating to wider existing models, it is unlikely that we have not included any study that would affect the results of our review.

Implications and future research

It is now accepted that the PSA test and its derivatives have some limitations for detecting PCa as defined by subsequent biopsy [86]. As a consequence, a considerable number of PCa prediction models have been built to improve prediction accuracy. This has resulted in a plethora of PCa risk prediction tools, with to date more than 100 models described [87 88]. There is evidence that some of these models show benefit and have better performance over just PSA measurement alone [20]. It also has been demonstrated that some of these models out-performed clinical experts in predicting PCa [28 29]. Although such models are not designed to replace specialist clinical judgment or patient preferences [76 86 89], they can help in patient counselling and aid clinicians to decide whether a prostate biopsy should be taken or not [77 89 90].

Given the small number of risk prediction models for PCa that do not incorporate clinical or genetic tests, the discrimination of these reviewed models ranged between poor to moderate (AUC range ~0.65 to ~0.75); in addition there were some issues relating to their study design and analysis raises the risk of bias. Consequently none of these

For peer review only

models could be currently recommended for use in a primary care and community health care setting. Several guidelines are against using PSA test based screening for PCa; the US Preventive Services task force, the Canadian task force on preventive health and the American College of Preventive Medicine do not currently recommend PSA-based testing due to insufficient evidence [91-93]. This has made it difficult, so far, to convince policymakers to adopt PCa screening programme.

The first guideline of PROSTATE CANCER UK states *“In the future, health professionals should look at a man’s PSA level alongside other known risk factors as part of a risk assessment tool, when one becomes available.”* [94] However, the vast majority of the current PCa risk prediction models are not suitable for routine use as they include clinical and genetic tests and are not validated externally in other cohorts. Therefore, the main challenge in the UK, remains to develop a risk prediction tool that is reliable, cheap, is applicable for as wide an ethnicity as possible, and most importantly is easy to use and can be implemented at a primary care level [95].

The value of such risk tools is that they will help to stratify men at high risk of developing PCa earlier so that they have appropriate management and/or surveillance programme as early as possible and therefore, may fit into the clinical pathway. Such tools should help physicians have a better understanding of the risk for this disease and simplify the procedures and discussions with patients when recommending further specialist-led investigations such as DRE and/or MRI where a decision on whether a biopsy should or not perform is concluded. Furthermore, using the appropriate risk prediction tool will avoid men from undergoing inappropriate further and frequent testing [95]. This will reduce any associated costs of inappropriate tests and decrease the burden on health care delivery systems.

It is crucial to address these issues by identify all possible risk factors for PCa that are non-clinical, non-genetic, and easy to use and interpret. There remains a pressing need to develop a risk prediction tool in the future using all appropriate factors (potentially also including genetics once there is infrastructure in place for genetic testing in the primary care and the cost comes down) into a robust multivariable analysis and validate the model externally to eliminate applicability and generalisability concerns. Only when

For peer review only

this is achieved will it be possible to introduce a prostate cancer screening programme fit for purpose.

CONCLUSION

There is a paucity of suitable low cost risk models that incorporate non-clinical, non-genetic inputs and which can be used at a primary care level and in other community health services. Existing models have limitations reflecting both study design and reporting performance measures. Future research should take into account these key issues and explore other risk factors for incorporation into further models.

ACKNOWLEDGEMENT

MA is supported by Kuwait's Ministry of Health. KM and AL are supported by the NIHR Manchester Biomedical Research Centre and by the ICEP ("This work was also supported by CRUK [grant number C18281/A19169]). KM is also supported by the Allan Turing Institute.

AUTHOR CONTRIBUTIONS

MA, AL and KM were involved in study conception, idea and design. Data acquisition and extraction was obtained by MA. Data synthesis and interpretation was carried out by MA, AL and WO. MA and AL drafted the manuscript. All authors approved the final version of manuscript. KM is the study guarantor.

FUNDING

No funding is required for conducting this study.

COMPETING INTERESTS

All authors have completed the ICMJE uniform disclosure form at www.icmje.org/coi_disclosure.pdf and declare: no support from any organisation for the submitted work; no financial relationships with any organisations that might have an interest in the submitted work in the previous three years; no other relationships or activities that could appear to have influenced the submitted work.

ETHICAL APPROVAL

Not applicable as this study did not involve any personal data.

For peer review only

DATA SHARING

Data extraction sheet and the PROBAST assessment form for risk of bias is available upon request.

Figure legend

Figure 1: Flow diagram of studies included using PRISMA method

REFERENCES

1. Bray F, Ferlay J, Soerjomataram I, et al. Global cancer statistics 2018: GLOBOCAN estimates of incidence and mortality worldwide for 36 cancers in 185 countries. *CA: a cancer journal for clinicians* 2018;68(6):394-424.
2. Cancer Research UK. Prostate cancer statistics 2017 [Available from: https://www.cancerresearchuk.org/health-professional/cancer-statistics/statistics-by-cancer-type/prostate-cancer#heading-One.
3. Center MM, Jemal A, Lortet-Tieulent J, et al. International variation in prostate cancer incidence and mortality rates. *European urology* 2012;61(6):1079-92.
4. Zhou CK, Check DP, Lortet-Tieulent J, et al. Prostate cancer incidence in 43 populations worldwide: An analysis of time trends overall and by age group. *Int J Cancer* 2016;138(6):1388-400.
5. Dijkstra S, Mulders P, Schalken J. Clinical use of novel urine and blood based prostate cancer biomarkers: a review. *Clin Biochem* 2014;47(10-11):889-96.
6. Fontanella P, Benecchi L, Grasso A, et al. Decision-making tools in prostate cancer: from risk grouping to nomograms. *Minerva Urol Nefrol* 2017;69(6):556-66.
7. Stephan C, Ralla B, Jung K. Prostate-specific antigen and other serum and urine markers in prostate cancer. *Biochim Biophys Acta* 2014;1846(1):99-112.
8. Thompson IM, Pauler DK, Goodman PJ, et al. Prevalence of prostate cancer among men with a prostate-specific antigen level≤ 4.0 ng per milliliter. *N Engl J Med* 2004;350(22):2239-46.
9. Pereira-Azevedo N, Verbeek JFM, Nieboer D, et al. Head-to-head comparison of prostate cancer risk calculators predicting biopsy outcome. *Transl Androl Urol* 2018;7(1):18-26.
10. Stephan C, Cammann H, Meyer H-A, et al. PSA and new biomarkers within multivariate models to improve early detection of prostate cancer. *Cancer lett* 2007;249(1):18-29.
11. Polascik TJ, Oesterling JE, Partin AW. Prostate specific antigen: a decade of discovery-what we have learned and where we are going. *J Urol* 1999;162(2):293-306.
12. Stamey TA, Caldwell M, McNEAL JE, et al. The prostate specific antigen era in the United States is over for prostate cancer: what happened in the last 20 years? *J Urol* 2004;172(4 Pt 1):1297-301.

For peer review only

13. Bunting PS. A guide to the interpretation of serum prostate specific antigen levels. *Clin Biochem* 1995;28(3):221-41.

14. Catalona WJ, Partin AW, Sanda MG, et al. A multi-center study of [− 2] pro-prostate-specific antigen (PSA) in combination with PSA and free PSA for prostate cancer detection in the 2.0 to 10.0 ng/mL PSA range. *J Urol* 2011;185(5):1650-55.

15. Mistry K, Cable G. Meta-analysis of prostate-specific antigen and digital rectal examination as screening tests for prostate carcinoma. *The Journal of the American Board of Family Practice* 2003;16(2):95-101. [published Online First: 2003/04/01]

16. Guazzoni G, Nava L, Lazzeri M, et al. Prostate-specific antigen (PSA) isoform p2PSA significantly improves the prediction of prostate cancer at initial extended prostate biopsies in patients with total PSA between 2.0 and 10 ng/ml: results of a prospective study in a clinical setting. *Eur Urol* 2011;60(2):214-22.

17. Heijnsdijk EA, der Kinderen A, Wever EM, et al. Overdetection, overtreatment and costs in prostate-specific antigen screening for prostate cancer. *British journal of cancer* 2009;101(11):1833-8. doi: 10.1038/sj.bjc.6605422 [published Online First: 2009/11/12]

18. Roobol MJ. Is prostate cancer screening bad or good? Summary of a debate at the innovation in urology meeting, September 17-19, 2010, Milan, Italy. *Eur Urol* 2011;59(3):359-62. doi: 10.1016/j.eururo.2010.10.036 [published Online First: 2010/11/03]

19. Mottet N, Bellmunt J, Bolla M, et al. EAU-ESTRO-SIOG Guidelines on Prostate Cancer. Part 1: Screening, Diagnosis, and Local Treatment with Curative Intent. *Eur Urol* 2017;71(4):618-29. doi: 10.1016/j.eururo.2016.08.003 [published Online First: 2016/08/30]

20. Schroder F, Kattan MW. The comparability of models for predicting the risk of a positive prostate biopsy with prostate-specific antigen alone: a systematic review. *Eur Urol* 2008;54(2):274-90.

21. Ross PL, Scardino PT, Kattan MW. A catalog of prostate cancer nomograms. *J Urol* 2001;165(5):1562-8. [published Online First: 2001/05/09]

22. Shariat SF, Karakiewicz PI, Suardi N, et al. Comparison of nomograms with other methods for predicting outcomes in prostate cancer: a critical analysis of the literature. *Clinical cancer research : an official journal of the American Association for Cancer Research* 2008;14(14):4400-7. doi: 10.1158/1078-0432.Ccr-07-4713 [published Online First: 2008/07/17]

23. Hu X, Cammann H, Meyer HA, et al. Artificial neural networks and prostate cancer--tools for diagnosis and management. *Nature reviews Urology* 2013;10(3):174-82. doi: 10.1038/nrurol.2013.9 [published Online First: 2013/02/13]

24. Caras RJ, Sterbis JR. Prostate cancer nomograms: a review of their use in cancer detection and treatment. *Current urology reports* 2014;15(3):391. doi: 10.1007/s11934-013-0391-0 [published Online First: 2014/01/24]

25. Iremashvili V, Soloway MS, Pelaez L, et al. Comparative validation of nomograms predicting clinically insignificant prostate cancer. *Urology* 2013;81(6):1202-8. doi: 10.1016/j.urology.2013.01.062 [published Online First: 2013/04/09]

For peer review only

26. Louie KS, Seigneurin A, Cathcart P, et al. Do prostate cancer risk models improve the predictive accuracy of PSA screening? A meta-analysis. *Annals of Oncology* 2015;26:848-64. doi: 10.1093/annonc/mdu525

27. Shariat SF, Kattan MW, Vickers AJ, et al. Critical review of prostate cancer predictive tools. *Future oncology (London, England)* 2009;5(10):1555-84. doi: 10.2217/fon.09.121 [published Online First: 2009/12/17]

28. Ross PL, Gerigk C, Gonen M, et al. Comparisons of nomograms and urologists' predictions in prostate cancer. *Seminars in urologic oncology* 2002;20(2):82-8. [published Online First: 2002/05/16]

29. Walz J, Gallina A, Perrotte P, et al. Clinicians are poor raters of life-expectancy before radical prostatectomy or definitive radiotherapy for localized prostate cancer. *BJU Int* 2007;100(6):1254-8.

30. Ouzaid I, Yates DR, Hupertan V, et al. A direct comparison of the diagnostic accuracy of three prostate cancer nomograms designed to predict the likelihood of a positive initial transrectal biopsy. *The Prostate* 2012;72(11):1200-6. doi: 10.1002/pros.22470 [published Online First: 2012/01/04]

31. Eastham JA, May R, Robertson JL, et al. Development of a nomogram that predicts the probability of a positive prostate biopsy in men with an abnormal digital rectal examination and a prostate-specific antigen between 0 and 4 ng/mL. *Urology* 1999;54(4):709-13.

32. Garzotto M, Hudson RG, Peters L, et al. Predictive modeling for the presence of prostate carcinoma using clinical, laboratory, and ultrasound parameters in patients with prostate specific antigen levels < or = 10 ng/mL. *Cancer* 2003;98(7):1417-22. doi: 10.1002/cncr.11668 [published Online First: 2003/09/26]

33. Karakiewicz PI, Benayoun S, Kattan MW, et al. Development and validation of a nomogram predicting the outcome of prostate biopsy based on patient age, digital rectal examination and serum prostate specific antigen. *J Urol* 2005;173(6):1930-4.

34. Kranse R, Roobol M, Schroder FH. A graphical device to represent the outcomes of a logistic regression analysis. *The Prostate* 2008;68(15):1674-80. doi: 10.1002/pros.20840 [published Online First: 2008/08/21]

35. Thompson IM, Ankerst DP, Chi C, et al. Assessing prostate cancer risk: results from the Prostate Cancer Prevention Trial. *J Natl Cancer Inst* 2006;98(8):529-34.

36. Schröder FH, Kruger AB, Rietbergen J, et al. Evaluation of the digital rectal examination as a screening test for prostate cancer. *Journal of the National Cancer Institute* 1998;90(23):1817-23.

37. Vis AN, Hoedemaeker RF, Roobol M, et al. Tumor characteristics in screening for prostate cancer with and without rectal examination as an initial screening test at low PSA (0.0–3.9 ng/ml). *The Prostate* 2001;47(4):252-61.

38. Smith DS, Catalona WJ. Interexaminer variability of digital rectal examination in detecting prostate cancer. *Urology* 1995;45(1):70-74.

For peer review only

39. Barocas DA, Cowan JE, Smith JA, Jr., et al. What percentage of patients with newly diagnosed carcinoma of the prostate are candidates for surveillance? An analysis of the CaPSURE database. 2008

40. Vis A, Kranse R, Roobol M, et al. Serendipity in detecting disease in low prostate-specific antigen ranges. *BJU international* 2002;89(4):384-89.

41. Littrup PJ, Bailey SE. Prostate cancer: the role of transrectal ultrasound and its impact on cancer detection and management. *Radiologic Clinics of North America* 2000;38(1):87-113.

42. Gerber GS, Chodak GW. Routine screening for cancer of the prostate. *JNCI: Journal of the National Cancer Institute* 1991;83(5):329-35.

43. Gelfand DE, Parzuchowski J, Cort M, et al. Digital rectal examinations and prostate cancer screening: attitudes of African American men. *Oncology Nursing Forum-Oncology Nursing Society* 1995;22(8):1253-58.

44. Myers RE, Wolf TA, McKee L, et al. Factors associated with intention to undergo annual prostate cancer screening among African American men in Philadelphia. *Cancer: Interdisciplinary International Journal of the American Cancer Society* 1996;78(3):471-79.

45. Consedine NS, Horton D, Ungar T, et al. Fear, knowledge, and efficacy beliefs differentially predict the frequency of digital rectal examination versus prostate specific antigen screening in ethnically diverse samples of older men. *American journal of men's health* 2007;1(1):29-43.

46. Consedine NS, Morgenstern AH, Kudadjie-Gyamfi E, et al. Prostate cancer screening behavior in men from seven ethnic groups: the fear factor. *Cancer Epidemiology and Prevention Biomarkers* 2006;15(2):228-37.

47. Ukoli FA, Patel K, Hargreaves M, et al. A tailored prostate cancer education intervention for low-income African Americans: Impact on knowledge and screening. *Journal of health care for the poor and underserved* 2013;24(1):311-31.

48. Ogunsanya ME, Brown CM, Odedina FT, et al. Beliefs regarding prostate cancer screening among black males aged 18 to 40 Years. *American journal of men's health* 2017;11(1):41-53.

49. Clarke-Tasker VA. What we thought we knew: African American males' perceptions of prostate cancer and screening methods. *ABNF Journal* 2002;13(3):56.

50. Romero FR, Romero AW, Brenny Filho T, et al. Patients' perceptions of pain and discomfort during digital rectal exam for prostate cancer screening. *Archivos espanoles de urologia* 2008;61(7):850-54.

51. Essink-Bot M-L, de Koning HJ, Nijs HG, et al. Short-term effects of population-based screening for prostate cancer on health-related quality of life. *Journal of National Cancer Institute* 1998;90(12):925-31.

52. Appleton L, Wyatt D, Perkins E, et al. The impact of prostate cancer on men's everyday life. *European journal of cancer care* 2015;24(1):71-84.

For peer review only

53. Lee DJ, Consedine NS, Spencer BA. Barriers and facilitators to digital rectal examination screening among African-American and African-Caribbean men. *Urology* 2011;77(4):891-98.

54. Nagler HM, Gerber EW, Homel P, et al. Digital rectal examination is barrier to population-based prostate cancer screening. *Urology* 2005;65(6):1137-40.

55. Moher D, Liberati A, Tetzlaff J, et al. Preferred reporting items for systematic reviews and meta-analyses: the PRISMA statement. *PLoS medicine* 2009;6(7):e1000097.

56. Park SH, Goo JM, Jo CH. Receiver operating characteristic (ROC) curve: practical review for radiologists. *Korean journal of radiology* 2004;5(1):11-8. doi: 10.3348/kjr.2004.5.1.11 [published Online First: 2004/04/06]

57. Han K, Song K, Choi BW. How to Develop, Validate, and Compare Clinical Prediction Models Involving Radiological Parameters: Study Design and Statistical Methods. *Korean journal of radiology* 2016;17(3):339-50. doi: 10.3348/kjr.2016.17.3.339 [published Online First: 2016/05/03]

58. Steyerberg EW. Clinical prediction models: a practical approach to development, validation, and updating: Springer Science & Business Media 2008.

59. Pavlou M, Ambler G, Seaman SR, et al. How to develop a more accurate risk prediction model when there are few events. *Bmj* 2015;351:h3868.

60. Wolff RF, Moons KGM, Riley RD, et al. PROBAST: A Tool to Assess the Risk of Bias and Applicability of Prediction Model Studies. *Ann Intern Med* 2019;170(1):51-58.

61. Carlson GD, Calvanese CB, Partin AW. An algorithm combining age, total prostate-specific antigen (PSA), and percent free PSA to predict prostate cancer: results on 4298 cases. *Urology* 1998;52(3):455-61.

62. Babaian RJ, Fritsche H, Ayala A, et al. Performance of a neural network in detecting prostate cancer in the prostate-specific antigen reflex range of 2.5 to 4.0 ng/mL. *Urology* 2000;56(6):1000-6.

63. Jansen FH, van Schaik RH, Kurstjens J, et al. Prostate-specific antigen (PSA) isoform p2PSA in combination with total PSA and free PSA improves diagnostic accuracy in prostate cancer detection. *European urology* 2010;57(6):921-27.

64. Hill OT, Mason TJ, Schwartz SW, et al. Improving prostate cancer detection in veterans through the development of a clinical decision rule for prostate biopsy. *BMC Urol* 2013;13:6.

65. Lazzeri M, Haese A, de la Taille A, et al. Serum isoform [-2]proPSA derivatives significantly improve prediction of prostate cancer at initial biopsy in a total PSA range of 2-10 ng/ml: a multicentric European study. *Eur Urol* 2013;63(6):986-94.

66. Moons KG, de Groot JA, Bouwmeester W, et al. Critical appraisal and data extraction for systematic reviews of prediction modelling studies: the CHARMS checklist. *PLoS medicine* 2014;11(10)

67. Porter CR, O'Donnell C, Crawford ED, et al. Predicting the outcome of prostate biopsy in a racially diverse population: a prospective study. *Urology* 2002;60(5):831-35.

For peer review only

68. Porter CR, Gamito EJ, Crawford ED, et al. Model to predict prostate biopsy outcome in large screening population with independent validation in referral setting. *Urology* 2005;65(5):937-41.

69. Catalona WJ, Bartsch G, Rittenhouse HG, et al. Serum pro-prostate specific antigen preferentially detects aggressive prostate cancers in men with 2 to 4 ng/ml prostate specific antigen. *J Urol* 2004;171(6 Pt 1):2239-44.

70. Sokoll LJ, Sanda MG, Feng Z, et al. A prospective, multicenter, National Cancer Institute Early Detection Research Network study of [-2]proPSA: improving prostate cancer detection and correlating with cancer aggressiveness. *Cancer Epidemiol Biomarkers Prev* 2010;19(5):1193-200.

71. Sokoll LJ, Wang Y, Feng Z, et al. [-2]proenzyme prostate specific antigen for prostate cancer detection: a national cancer institute early detection research network validation study. *J Urol* 2008;180(2):539-43; discussion 43.

72. Stephan C, Vincendeau S, Houlgatte A, et al. Multicenter evaluation of [-2]proprostate-specific antigen and the prostate health index for detecting prostate cancer. *Clin Chem* 2013;59(1):306-14.

73. Le BV, Griffin CR, Loeb S, et al. [-2]Proenzyme prostate specific antigen is more accurate than total and free prostate specific antigen in differentiating prostate cancer from benign disease in a prospective prostate cancer screening study. *J Urol* 2010;183(4):1355-9.

74. Loeb S, Sokoll LJ, Broyles DL, et al. Prospective multicenter evaluation of the Beckman Coulter Prostate Health Index using WHO calibration. *J Urol* 2013;189(5):1702-06.

75. Lughezzani G, Lazzeri M, Haese A, et al. Multicenter European external validation of a Prostate Health Index–based nomogram for predicting prostate cancer at extended biopsy. *Eur Urol* 2014;66(5):906-12.

76. Chun FK-H, Briganti A, Graefen M, et al. Development and external validation of an extended 10-core biopsy nomogram. *European urology* 2007;52:436-44.

77. Herman MP, Dorsey P, John M, et al. Techniques and predictive models to improve prostate cancer detection. *Cancer* 2009;115(S13):3085-99.

78. Trottier G, Roobol MJ, Lawrentschuk N, et al. Comparison of risk calculators from the Prostate Cancer Prevention Trial and the European Randomized Study of Screening for Prostate Cancer in a contemporary Canadian cohort. *BJU Int* 2011;108(8 Pt 2):E237-44.

79. Collins GS, de Groot JA, Dutton S, et al. External validation of multivariable prediction models: a systematic review of methodological conduct and reporting. *BMC Med Res Methodol* 2014;14(1):40.

80. Miyake H, Kurahashi T, Takenaka A, et al. Improved accuracy for predicting the Gleason score of prostate cancer by increasing the number of transrectal biopsy cores. *Urol Int* 2007;79(4):302-6.

81. Chun FK, Briganti A, Graefen M, et al. Development and external validation of an extended 10-core biopsy nomogram. *Eur Urol* 2007;52(2):436-44.

For peer review only

82. Aus G, Abbou CC, Bolla M, et al. EAU guidelines on prostate cancer. *Eur Urol* 2005;48(4):546-51.

83. Vickers AJ, Ulmert D, Serio AM, et al. The predictive value of prostate cancer biomarkers depends on age and time to diagnosis: towards a biologically-based screening strategy. *Int J Cancer* 2007;121(10):2212-7.

84. Steuber T, O'Brien MF, Lilja H. Serum markers for prostate cancer: a rational approach to the literature. *Eur Urol* 2008;54(1):31-40.

85. Carlsson S, Assel M, Sjoberg D, et al. Influence of blood prostate specific antigen levels at age 60 on benefits and harms of prostate cancer screening: population based cohort study. *Bmj* 2014;348:g2296.

86. Cavadas V, Osorio L, Sabell F, et al. Prostate cancer prevention trial and European randomized study of screening for prostate cancer risk calculators: a performance comparison in a contemporary screened cohort. *Eur Urol* 2010;58(4):551-8.

87. Vickers AJ. Prediction models: revolutionary in principle, but do they do more good than harm. *J Clin Oncol* 2011;29(22):2951-52.

88. Shariat SF, Karakiewicz PI, Margulis V, et al. Inventory of prostate cancer predictive tools. *Curr Opin Urol* 2008;18(3):279-96.

89. Kawakami S, Numao N, Okubo Y, et al. Development, validation, and head-to-head comparison of logistic regression-based nomograms and artificial neural network models predicting prostate cancer on initial extended biopsy. *Eur Urol* 2008;54(3):601-11.

90. Hernandez DJ, Han M, Humphreys EB, et al. Predicting the outcome of prostate biopsy: comparison of a novel logistic regression-based model, the prostate cancer risk calculator, and prostate-specific antigen level alone. *BJU Int* 2009;103(5):609-14.

91. Moyer VA. Screening for prostate cancer: US Preventive Services Task Force recommendation statement. *Ann Intern Med* 2012;157(2):120-34.

92. Bell N, Connor Gorber S, Shane A, et al. Recommendations on screening for prostate cancer with the prostate-specific antigen test. *CMAJ* 2014;186(16):1225-34.

93. Lim LS, Sherin K, Committee APP. Screening for prostate cancer in US men: ACPM position statement on preventive practice. *Am J Prev Med* 2008;34(2):164-70.

94. Prostate Cancer UK. Consensus statements on PSA testing in asymptomatic men in the UK - information for health professionals 2016 [Available from: https://prostatecanceruk.org/about-us/projects-and-policies/consensus-on-psa-testing accessed 20-8 2019.

95. Frame I, Cant S. Current challenges in prostate cancer: an interview with Prostate Cancer UK. *BMC Med* 2015;13(1):166.

For peer review only

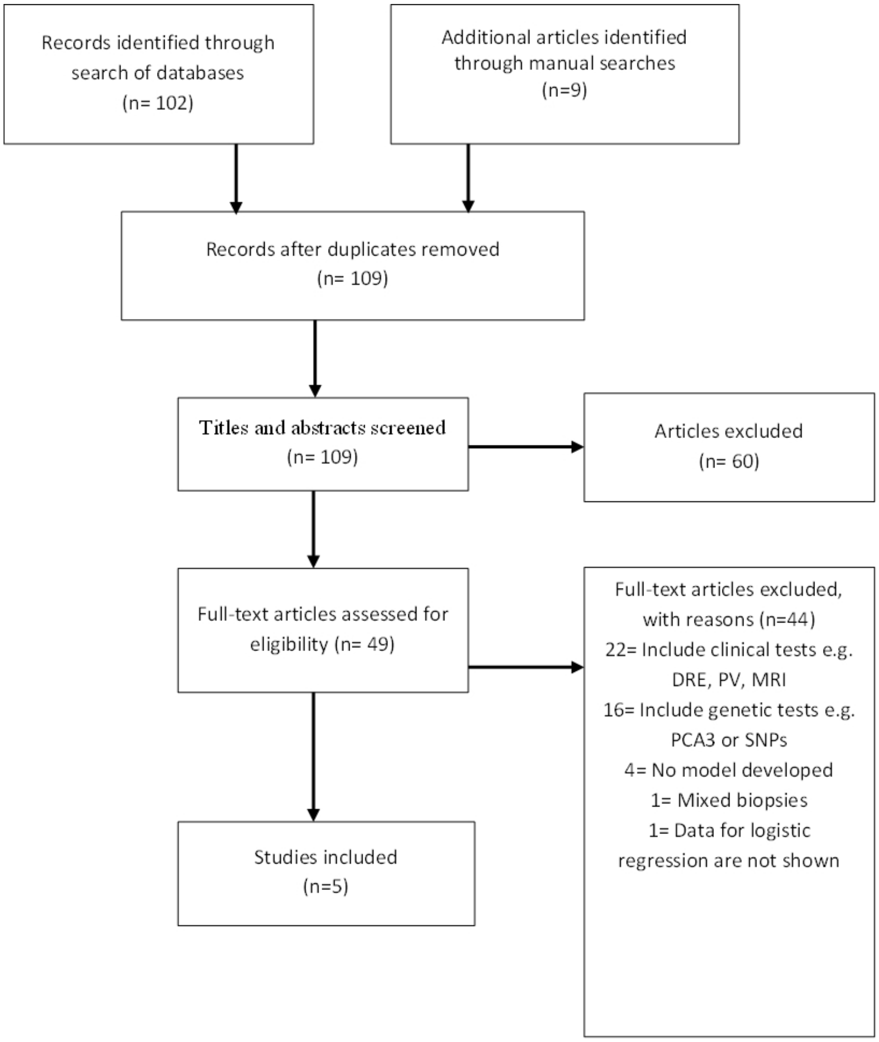

Flow diagram of studies included using PRISMA method

78x91mm (300 x 300 DPI)

Search Strategy

1) * Prostatic Neoplasms/
2) Initial biopsy.mp
3) First biopsy.mp
4) * "Early Detection of Cancer"/mt [Methods]
5) 2 or 3 or 4
6) 1 and 5
7) Nomograms/
8) "Neural Networks (Computer)"/
9) Risk Assessment/
10) Models, Statistical/
11) 7 or 8 or 9 or 10
12) 6 and 11
13) Limit 12 to English language
14) Limit 13 to abstracts

For peer review only

Table S2: Search results for independent studies that did external validation for included models.

Study	No. of citations in Google Scholar	No. of citations in Medline	No. of external validation found in Google Scholar	No. of external validation found in Medline
Carlson, 1998	117	20	Null	Null
Babaian, 2000	128	13	Null	Null
Jansen, 2010	231	44	Null	Null
Hill, 2010	5	0	Null	Null
Lazzeri, 2013	195	0	Null	Null

For peer review only

Supp Table 1: Type of assays used to measure PSA and free PSA.

Study	PSA Assay	Free PSA Assay
Carlson, 1998	PA immunoassay (Tosoh)	Investigational double-antibody radioimmunometric assay (PSA II, DIANON Systems)
Babaian, 2000	PSA immunometric assay (Tosoh)	Tandem R assay (Beckman Coulter, San Diego, Calif)
Jansen, 2010	Access 2 Immunoassay System (Beckman Coulter, Brea, CA, USA)	Access 2 Immunoassay System (Beckman Coulter, Brea, CA, USA)
Hill, 2013	N/A	N/A
Lazzeri, 2013	Access 2 Immunoassay System (Beckman Coulter, Brea, CA, USA)	Access 2 Immunoassay System (Beckman Coulter, Brea, CA, USA)

For peer review only

Reporting checklist for systematic review and meta-analysis.

Based on the PRISMA guidelines.

Instructions to authors

Complete this checklist by entering the page numbers from your manuscript where readers will find each of the items listed below.

Your article may not currently address all the items on the checklist. Please modify your text to include the missing information. If you are certain that an item does not apply, please write "n/a" and provide a short explanation.

Upload your completed checklist as an extra file when you submit to a journal.

In your methods section, say that you used the PRISMAreporting guidelines, and cite them as:

Moher D, Liberati A, Tetzlaff J, Altman DG, The PRISMA Group. Preferred Reporting Items for Systematic Reviews and Meta-Analyses: The PRISMA Statement

		Reporting Item	Page Number
**Title**			
	#1	Identify the report as a systematic review, meta-analysis, or both.	3
**Abstract**			
Structured summary	#2	Provide a structured summary including, as applicable: background; objectives; data sources; study eligibility criteria, participants, and interventions; study appraisal and synthesis methods; results; limitations; conclusions and implications of key findings; systematic review registration number	3-4
**Introduction**			
Rationale	#3	Describe the rationale for the review in the context of what is already known.	5-6
Objectives	#4	Provide an explicit statement of questions being addressed with	6

For peer review only

		reference to participants, interventions, comparisons, outcomes, and study design (PICOS).	
**Methods**			
Protocol and registration	#5	Indicate if a review protocol exists, if and where it can be accessed (e.g., Web address) and, if available, provide registration information including the registration number.	n/a
Eligibility criteria	#6	Specify study characteristics (e.g., PICOS, length of follow-up) and report characteristics (e.g., years considered, language, publication status) used as criteria for eligibility, giving rational	6
Information sources	#7	Describe all information sources in the search (e.g., databases with dates of coverage, contact with study authors to identify additional studies) and date last searched.	6
Search	#8	Present full electronic search strategy for at least one database, including any limits used, such that it could be repeated.	6
Study selection	#9	State the process for selecting studies (i.e., for screening, for determining eligibility, for inclusion in the systematic review, and, if applicable, for inclusion in the meta-analysis).	7
Data collection process	#10	Describe the method of data extraction from reports (e.g., piloted forms, independently by two reviewers) and any processes for obtaining and confirming data from investigators.	7
Data items	#11	List and define all variables for which data were sought (e.g., PICOS, funding sources), and any assumptions and simplifications made.	8
Risk of bias in individual studies	#12	Describe methods used for assessing risk of bias in individual studies (including specification of whether this was done at the study or outcome level, or both), and how this information is to be used in any data synthesis.	8
Summary measures	#13	State the principal summary measures (e.g., risk ratio, difference in means).	7
Planned methods of analyis	#14	Describe the methods of handling data and combining results of studies, if done, including measures of consistency (e.g., I2) for each meta-analysis.	6-8
Risk of bias	#15	Specify any assessment of risk of bias that may affect the	15-16

For peer review only

across studies		cumulative evidence (e.g., publication bias, selective reporting within studies).	
Additional analyses	#16	Describe methods of additional analyses (e.g., sensitivity or subgroup analyses, meta-regression), if done, indicating which were pre-specified.	n/a
**Results**			
Study selection	#17	Give numbers of studies screened, assessed for eligibility, and included in the review, with reasons for exclusions at each stage, ideally with a flow diagram.	8-9
Study characteristics	#18	For each study, present characteristics for which data were extracted (e.g., study size, PICOS, follow-up period) and provide the citation.	8-9
Risk of bias within studies	#19	Present data on risk of bias of each study and, if available, any outcome-level assessment (see Item 12).	15-17
Results of individual studies	#20	For all outcomes considered (benefits and harms), present, for each study: (a) simple summary data for each intervention group and (b) effect estimates and confidence intervals, ideally with a forest plot.	13-15
Synthesis of results	#21	Present the main results of the review. If meta-analyses are done, include for each, confidence intervals and measures of consistency.	12-15
Risk of bias across studies	#22	Present results of any assessment of risk of bias across studies (see Item 15).	15-17
Additional analysis	#23	Give results of additional analyses, if done (e.g., sensitivity or subgroup analyses, meta-regression [see Item 16]).	n/a
**Discussion**			
Summary of Evidence	#24	Summarize the main findings, including the strength of evidence for each main outcome; consider their relevance to key groups (e.g., health care providers, users, and policy makers	18-21
Limitations	#25	Discuss limitations at study and outcome level (e.g., risk of bias), and at review level (e.g., incomplete retrieval of identified research, reporting bias).	21-22

For peer review only

Conclusions	#26	Provide a general interpretation of the results in the context of other evidence, and implications for future research.	23

Funding

Funding	#27	Describe sources of funding or other support (e.g., supply of data) for the systematic review; role of funders for the systematic review.	24

The PRISMA checklist is distributed under the terms of the Creative Commons Attribution License CC-BY. This checklist was completed on 30. September 2019 using https://www.goodreports.org/, a tool made by the EQUATOR Network in collaboration with Penelope.ai

For peer review only

BMJ Open

Prediction models for prostate cancer to be used in the primary care setting: a systematic review.

Journal:	*BMJ Open*
Manuscript ID	bmjopen-2019-034661.R2
Article Type:	Original research
Date Submitted by the Author:	21-May-2020
Complete List of Authors:	Aladwani , Mohammad; The University of Manchester, Division of Population Health, Health Services Research and Primary Care School of Health Sciences Faculty of Biology, Medicine and Health<br>Lophatananon, Artitaya ; The University of Manchester, Division of Population Health, Health Services Research and Primary Care School of Health Sciences Faculty of Biology, Medicine and Health<br>Ollier, William; The University of Manchester, Division of Population Health, Health Services Research and Primary Care School of Health Sciences Faculty of Biology, Medicine and Health; Manchester Metropolitan University Faculty of Science and Engineering, School of Healthcare Science<br>Muir, Kenneth; The University of Manchester, Division of Population Health, Health Services Research and Primary Care School of Health Sciences Faculty of Biology, Medicine and Health
**Primary Subject Heading**:	Public health
Secondary Subject Heading:	Urology, Epidemiology
Keywords:	EPIDEMIOLOGY, UROLOGY, Prostate disease < UROLOGY

SCHOLARONE™
Manuscripts

I, the Submitting Author has the right to grant and does grant on behalf of all authors of the Work (as defined in the below author licence), an exclusive licence and/or a non-exclusive licence for contributions from authors who are: i) UK Crown employees; ii) where BMJ has agreed a CC-BY licence shall apply, and/or iii) in accordance with the terms applicable for US Federal Government officers or employees acting as part of their official duties; on a worldwide, perpetual, irrevocable, royalty-free basis to BMJ Publishing Group Ltd ("BMJ") its licensees and where the relevant Journal is co-owned by BMJ to the co-owners of the Journal, to publish the Work in this journal and any other BMJ products and to exploit all rights, as set out in our licence.

The Submitting Author accepts and understands that any supply made under these terms is made by BMJ to the Submitting Author unless you are acting as an employee on behalf of your employer or a postgraduate student of an affiliated institution which is paying any applicable article publishing charge ("APC") for Open Access articles. Where the Submitting Author wishes to make the Work available on an Open Access basis (and intends to pay the relevant APC), the terms of reuse of such Open Access shall be governed by a Creative Commons licence – details of these licences and which Creative Commons licence will apply to this Work are set out in our licence referred to above.

Other than as permitted in any relevant BMJ Author's Self Archiving Policies, I confirm this Work has not been accepted for publication elsewhere, is not being considered for publication elsewhere and does not duplicate material already published. I confirm all authors consent to publication of this Work and authorise the granting of this licence.

For peer review only

Full title: Prediction models for prostate cancer to be used in the primary care setting: a systematic review.

Institutional addresses and e-mail addresses of the contributing authors:

Dr Mohammad Aladwani

Division of Population Health, Health Services Research and Primary Care, School of Health Sciences Faculty of Biology, Medicine and Health, The University of Manchester, Manchester, M13 9PT, UK

E-mail: mohammad.al-adwani@postgrad.manchester.ac.uk

Dr Artitaya Lophatananon

Division of Population Health, Health Services Research and Primary Care, School of Health Sciences Faculty of Biology, Medicine and Health, The University of Manchester, Manchester, M13 9PT,UK

E-mail: artitaya.lophatananon@.manchester.ac.uk

Professor William Ollier,

Division of Population Health, Health Services Research and Primary Care, School of Health Sciences Faculty of Biology, Medicine and Health, The University of Manchester, Manchester, M13 9PT, UK

School of Healthcare Science, Faculty of Science and Engineering, Manchester Metropolitan University, John Dalton Building, Manchester, M1 5GD, UK

E-mail: Bill.Oliier@manchester.ac.uk

Address and contact details of the corresponding author:

Professor Kenneth R. Muir

Division of Population Health, Health Services Research and Primary Care,

School of Health Sciences, Faculty of Biology, Medicine and Health,

The University of Manchester, Manchester, M13 9PT, UK

E-mail: kenneth.muir@manchester.ac.uk

For peer review only

Telephone: +44-161-275-5518

Key words: Prostate cancer, prostate specific antigen, PSA, prostate cancer screening, risk Prediction, risk tools, prediction models, primary care, community.

For peer review only

Abstract

Objective

To identify risk prediction models for prostate cancer (PCa) that can be used in the primary care and community health settings.

Design

Systematic review.

Data sources

MEDLINE and EMBASE databases combined from inception and up to the end of January 2019.

Eligibility

Studies were included based on satisfying all the following criteria; (i) presenting an evaluation of PCa risk at initial biopsy in patients with no history of PCa, (ii) studies not incorporating an invasive clinical assessment or expensive biomarker/ genetic tests, (iii) inclusion of at least two variables with PSA being one of them, and (iv) studies reporting a measure of predictive performance. The quality of the studies and risk of bias was assessed by using the PROBAST tool.

Data extraction and synthesis

Relevant information extracted for each model included; the year of publication, source of data, type of model, number of subjects, country, age, PSA range, mean/median PSA, other variables included in the model, number of biopsy cores to assess outcomes, study endpoint (s), cancer detection, model validation, and model performance.

Results

An initial search yielded 109 potential studies, of which five met the set criteria. Four studies were cohort-based and one was a case-control study. PCa detection rate was between 20.6% and 55.8%. AUC was reported in four studies and ranged from 0.65 to 0.75. All models showed significant improvement in predicting PCa compared with being based on PSA alone. The difference in AUC between extended models and PSA alone was between 0.06 and 0.21.

For peer review only

Conclusions

Only a few PCa risk prediction models have the potential to be readily used in the primary health care or community health setting. Further studies are needed to investigate other potential variables that could be integrated into models to improve their clinical utility for PCa testing in a community setting.

For peer review only

Strengths and limitations

- The review focussed on risk prediction models for PCa for use in primary care.
- The PRISMA approach was followed in identifying relevant articles and reporting this study.
- We used the PROBAST tool to assess the quality and risk of bias in the included models.
- The search strategy was restricted to two databases and a manual search, to retrieve original studies.

INTRODUCTION

Prostate cancer (PCa) is the second most common cancer and the fifth leading cause of cancer-attributed death in men worldwide with an estimated incidence of 1,276,106 and 358,989 deaths in 2018 [1]. In the UK, around 47,200 new cases of PCa were reported in 2015, accounting for 26% of all new cancer cases in males. PCa deaths in the UK of were around 11,600 in 2016 [2]. The global projections of PCa incidence and mortality for 2030 are 1.7 and 0.5 million, respectively [3]. The highest incidence of PCa is seen in western societies [4]. The significant increase of PCa incidence and diagnosis over the last three decades can be attributed mainly to the widespread implementation of the PSA serum test after it had been introduced in the late 1980s [5 6].

The strong association of PSA with PCa [7 8], along with it being a relatively inexpensive test [9], has made PSA a key biomarker in the diagnostic processs of PCa and for the recommendation of a confirmatory prostate biopsy [7 9]. PSA is, however, not a cancer-specific marker [5 10]. Conditions such as benign prostate hypertrophy (BPH), prostatitis, and other non-malignant prostatic conditions can also elevate PSA level, thus introducing uncertainty to the application of the test [11-14]. This highlights limitations of the PSA test regarding its specificity and sensitivity and it being largely dependent on setting a ‘diagnostic’ cut-off point, which often leads to an unacceptable number of false-positive and false-negative results [5 10 15 16]. Such issues are likely to be the part of the explanation for the significant number of unnecessary biopsies currently performed each year. Such procedures are associated with adverse side effects for patients and also increases health care costs [17 18].

For peer review only

To address such PSA test limitations, researchers have incorporated other measurable factors into approaches for the early detection of PCa; these “risk assessment tools” are based on statistical models designed to improve the accuracy and performance of the PSA test [19-22]. Logistic regression and artificial neural network (ANNs) models are now considered to be the most common and effective statistical techniques in aiding development of new models to enhance early PCa diagnosis [23]. These PCa risk prediction models can be used to aid the testing of men for further investigations.

Currently in the UK, there is no population based screening programme for PCa. The ultimate goal of PCa screening is to find intermediate and high risk of PCa rather than low risk PCa that would not require treatment but will give emotional burden to the patient once detected and unnecessary treatment in some patients. An important potential advantage of the extended risk models is their ability to provide a more accurate estimation of PCa risk. This may ultimately lead to their use in patient counselling and decision-making [24-27]. Such models have already achieved better results in predicting probabilities of outcome compare to clinical judgment [28 29]. Furthermore, it has been reported that using such predictive models may minimize the rate of unnecessary biopsies [30].

Recently there has been a substantial increase in the development of predictive models to help clinicians assess risk and decide which man to send to clinical setting to further investigate for a possible diagnosis of PCa [22 26 30-35]. The majority of these models are designed for use in clinical settings, where costs are less of an issue and most include the need for a clinical examination such as digital rectal examination (DRE) or trans-rectal ultrasonography (TRUS). One of the main limitations of DRE is that it has poor performance, especially at low PSA levels, and it is highly subjective to inter-observer variability [36-38]. A meta-analysis study revealed that DRE has positive predictive value of only 18% [39]. Similarly, TRUS has been reported for having poor accuracy at low PSA levels [40 41] and small PCa might not be palpable on DRE or visualisation on TRUS [40]. Furthermore, less than 40% of PCa detected by DRE are potentially curable, making it less beneficial for early diagnosis [42]. Several studies showed that there are fear, anxiety and embarrassment among some men, in particular black men, regarding the DRE test [43-46]. Another disadvantage of the DRE is the fact it is a potentially uncomfortable test [47-

For peer review only

[52]. This may explain why the DRE is a barrier for some men to participate in PCa screening if it is including DRE test. Lee et al reported that 74%-84% of black men may not maintain annual DRE screening [53], while another study found that it may prevent 22% of men to participate [54]. Since TRUS need to be performed by a skilled urologist, this means men have to make an appointment with a clinic in different location, which makes the screening less convenience. As a result, men may feel reluctant to have such tests performed.

This systematic review of the literature was undertaken to identify risk prediction models that do not incorporate invasive or more costly clinical procedures or extensive biomarkers but have potential application for use in primary care and community settings. As low cost is a primary concern for community use, for this review we set an indicative threshold of approximately 3-5 times the cost of a PSA test for inclusion. This excluded a number of models that contain new and emerging biomarker or SNP panels. As the numbers of person referred to clinical setting costs are less of an issue. The performance of the models reviewed for detecting PCa at initial biopsy have been compared using 'reported area under the curve' (AUC) and/or sensitivity - specificity testing.

METHODS

The approach used to identify and select relevant articles was based on the application of the '*Preferred Reporting Items for Systematic Reviews and Meta-Analysis'* (PRISMA) [55].

Data sources and search strategy

A literature search was performed using Medline (via OVID) and Embase databases. The 'medical subject heading' (MeSH) terms, combined with Boolean logic operators 'AND' and 'OR', were applied to retrieve relevant articles. The terms used for the search were "Prostatic Neoplasms" AND ("Initial biopsy" OR "first biopsy" OR "early detection of cancer") AND ("nomograms" OR "artificial neural networks" OR "risk assessment" OR "statistical model"). The full search strategy is provided in a Supplementary File 1. All articles defined (published since the inception of the databases and up to the end of January 2019) were subsequently further filtered as being those only published in English language and with an abstract. Further to using the above search databases, the research

For peer review only

articles were selected manually from the reference lists of any relevant review articles. Google Scholar and Medline searches were also carried out to identify independent study for external validation for each model included in this review. The results are presented in Supplementary File 2.

Eligibility criteria

As this review focuses on PCa risk prediction based in community healthcare settings, all studies were selected on the following inclusion criteria (i) evaluating the risk for PCa at initial biopsy in subjects who had no prior history of PCa (ii) studies that reported "low cost" risk assessment tools (i.e. those not including more expensive genetic, biomarker test) or "invasive" clinical tests/examinations (such as DRE or TRUS) (iii) studies that included a minimum of two variables of which PSA had to be one of them [on the basis that an elevated PSA test in UK primary care is usually the first sign and rationale for suggesting a need for further investigation of PCa within NICE guidelines] and (iv) studies that reported AUC and/or sensitivity and specificity of the diagnostic/predictive tool. The exclusion criteria used were (i) articles with models that were built and based on repeat or mixed biopsies (ii) studies that only validate an existing model (iii) articles that were not published in English. There were no time boundaries regarding the publication year.

Screening of the titles, abstracts, and full-texts was carried out by two reviewers (MA, AL). Any concerns about the eligibility of a study were resolved by discussion with a third reviewer (KM).

Data extraction

A data extraction form was developed to collect all relevant information. For each study used in this review, the items extracted included; year of publication, source of data, type of model, number of subjects, country where it was performed, age, PSA range, mean/median PSA, number of biopsy cores, variables included in the model, study endpoint(s), cancer detection, model performance, and model validation.

Evaluating the performance of the risk models

Prediction models can be evaluated against various criteria. The most critical measurements of model performance are discrimination and calibration [27]. Discrimination refers to how well the prediction model can differentiate subjects in different outcome

For peer review only - http://bmjopen.bmj.com/site/about/guidelines.xhtml

classes according to their predicted risks. It is often assessed by measuring the area under the receiver operating characteristic (ROC) curve [56]. It also requires setting a series of thresholds to separate low and high ranges of predicted outcomes. A value of 0.5 indicates no discrimination, while a value of 1 indicates perfect discrimination. However, even with perfect discrimination, observed risk can differ from expected risk. Therefore, calibration has an important role in model evaluation [57]. Calibration represents the agreement between expected and observed outcomes [58]. A well-calibrated model is achieved when the calibration slope is close to 1. When the calibration slope is less than 1 it indicates that the model under-estimates low risks and over-estimate high risks [59].

Due to the heterogeneity of the studies included, conducting a meta-analysis was not applicable.

Study quality assessment

The quality of the studies included in this review was assessed using the PROBAST tool [60]. This tool has been developed specifically to assess the risk of bias and applicability for prediction model studies. The tool consists of four domains and has 20 signalling questions that facilitate to reach overall judgment of risk of bias, as well as issues relating to applicability.

Patient and Public involvement

No patients were involved in setting the research question or the outcome measures, nor were they involved in the design and implementation of the study. There are no plans to involve patients in dissemination.

RESULTS

A total of 102 publications were identified using the search strategy as shown in Fig.1. An additional nine articles were identified through manual searches from a bibliography of reviewed articles. At the first filter step, a total of 109 titles and abstracts were screened for eligibility after removing two duplicates. In the second filter step, 60 papers failed to meet the inclusion criteria and were excluded, resulting in 49 articles. The final step of filtering yielded only five studies that were considered to be eligible (i.e. passed all set

For peer review only

criteria) and were thus included in this systematic review. There is no independent study identified for external validation for included models.

Study characteristics

Four of the five studies included were based on cohort studies and one was a case-control study. The characteristics of each of these studies and populations are summarised in Table 1. Details of PSA assays used in the models are presented in Supplementary File 3.

For peer review only

Table 1: Characteristics of the included studies.

Author & year	Type of model	Type of study	Sample No.	Location	Population type	Age	Median Age	PSA range	Median PSA	No. of biopsy cores	Cancer detection
Carlson, 1998 [61]	Logistic regression	Cohort	Model dev = 3773<br>Validation = 525	Baltimore, USA	Referral	≥ 45	—	4-20 ng/ml	—	Sextant biopsy	32%
Babaian, 2000 [62]	Neural network = 3 ANNs	Cohort	151	Texas, USA	Screening program	40-75	62	2.5-4 ng/ml	—	11 cores	24.5%
Jansen, 2010 [63]	Logistic regression	Cohort	Site 1 = 405<br>Site 2 = 351	Site 1 from the Rotterdam arm of the European Study of screening for Prostate cancer<br>Site 2 Innsbruck, Austria	Screening program	≥ 50	Site 1 (66)<br>Site 2 (60)	2-10 ng/ml	~ 4.4	≥6 cores	Site 1 = 55.8%<br>Site 2 = 49.6%
Hill, 2013 [64]	Logistic regression	Case-control	1378	Florida, USA	Hospital referral	40-90	—	≥ 4 ng/ml	—	N/A	20.6%
Lazzeri, 2013 [65]	Logistic regression	Cohort	646	European multi-centre; Italy, Germany, France, Spain, and the UK	Referral	>45	—	2-10 ng/ml	~ 5.8	≥ 12 cores	40.1%

Subjects used to build the risk models varied across these studies. Of the five studies, three studies included men from referral populations [61 64 65] and two studies from screening programs [62 63]. The sample sizes ranged from 151 to 3,773 with three studies derived from US cohorts [61 62 64] and two from Europe [63 65]. Four studies used logistic regression methodology to build their model, whereas one study used an artificial neural network-based approach (ANN) [62]. The minimum age of participants was 40 years old [62 64] and the minimum PSA level was 2 ng/ml [63 65].

Variables in the model

Table 2 presents details of the variables used in each model. PSA level was used in all models, followed by free PSA (fPSA), age, and free-to-total PSA ratio (%fPSA). Other variables also reported in the models included; precursor of prostate-specific antigen (p2PSA), percentage of p2PSA to free PSA (%p2PSA), prostate health index (PHI), levels of haemoglobin, albumin, creatinine and red blood cell count (RBC), haematuria, mean corpuscular volume (MCV), and prostatic acid phosphatase.

For peer review only

Table 2: Variables used in the prostate cancer risk prediction models*.

Author & year	Variables used in the model				
	Total PSA	Free PSA	Percent free PSA	Age	Other variables
Carlson, 1998 [61]	✓		✓	✓	
Babaian, 2000 [62]	✓	✓		✓	Creatinine Kinase, Prostatic acid phosphatase
Jansen, 2010 [63] *(Site 1)*	✓	✓			p2PSA
Jansen, 2010 [63] *(Site 2)*	✓	✓			p2PSA
Hill, 2013 [64] *(Method 1)*	✓			✓	HGB, RBC, Haematuria, Creatinine, MCV, and ethnicity "Black"
Hill, 2013 [64] *(Method 2)*	✓			✓	HGB, RBC, Creatinine, and MCV
Lazzeri, 2013 [65] *(Model 1)*	✓	✓	✓		
Lazzeri, 2013 [65] *(Model 2)*	✓	✓	✓		p2PSA
Lazzeri, 2013 [65] *(Model 3)*	✓	✓	✓		%p2PSA
Lazzeri, 2013 [65] *(Model 4)*	✓	✓	✓		PHI

*HGB= Haemoglobin. RBC= Red blood cells. MCV= Mean corpuscular volume. PHI= Prostate Health Index. p2PSA= Precursor of prostate specific antigen, %p2PSA= p2PSA/fPSA

For peer review only

Outcome

The study endpoint also varied among the studies selected. Two studies evaluated the accuracy of detecting any PCa [61 62] and three studies examined the pathologic Gleason score [63-65]. Although Jansen et al, did not build a model to predict the aggressiveness of prostate cancer, they assessed the relationship of each variable individually with a Gleason score ≥7. PCa was determined by taking a needle biopsy. All subjects in the five studies underwent prostate biopsy. The least number of biopsy cores used were 6 [61 63] and the highest were ≥ 12 [65]. One study did not report the number of biopsy cores taken [64]. PCa rates ranged from 20.6% to 55.8%.

Evaluating the performance of the risk models

For predicting any PCa, the Jansen et al, model used data from the Rotterdam arm of the European Study of Screening for PCa (ESPRC). Their model achieved the highest discrimination value when compared to PSA alone (AUC of 0.755 vs. 0.585, respectively) [63]. The AUC values in other studies ranged from 0.648 to 0.74.

One study did not provide the AUC but instead reported an increase of 11% in specificity over percent free PSA alone with 95% sensitivity [61]. Lazzeri et al, [65] presented results from four separated models discriminating PCa with a Gleason score of ≥7. Lazzeri's model 2 (which includes the base model tPSA, fPSA and %fPSA in addition to p2PSA) and model 3 (which includes base model plus PHI) showed the highest levels of discrimination out of the 4 models with an AUC of 0.67. In the study of Hill [64] the authors classified PCa stages differently and built their two models accordingly. In Hill's first model, the difference in the discrimination was analysed and based on all PCa versus non-cancerous prostate conditions where the AUC for this model was 0.68 compared to 0.59 for PSA alone. In Hill's second model, the discrimination analysis was based on PCa stages II, III, IV versus PCa stage I, prostatic interstitial neoplasm (PIN), BPH and prostatitis where stages I, II, III and IV are parallel to T1, T2, T3/T4, and metastatic PCa respectively. The AUC for the second model was 0.72 compared to 0.63 for PSA alone. In general, four studies examined the AUC with PSA alone and all reported a benefit from the use of logistic regression or the trained ANN. Model performance and the differences between the AUC's for PSA alone and for the extended models are presented in Table 3.

For peer review only

Table 3: The difference of area under the curve (AUC) for PSA alone and extended model.

Study	AUC for PSA	AUC for model	ΔAUC (Model – PSA)
Carlson [61]	NA	NA	NA
Babaian [62]	0.64 %fPSA	0.74	0.1
Jansen [63] *(site 1)*	0.58	0.75	0.17
Jansen [63] *(site 2)*	0.53	0.7	0.16
Hill [64] *(method 1)*	0.59	0.68	0.09
Hill [64] *(method 2)*	0.63	0.72	0.09
Lazzeri [65]	0.50 for any PC<br>0.54 for Gleason score ≥7	Model 1 = 0.65<br>Model 1 (Gleason score≥7) = 0.60	0.15<br>0.06
		Model 2= 0.71<br>Model 2 (Gleason score≥7) = 0.67	0.21<br>0.13
		Model 3= 0.704<br>Model 3 (Gleason score≥7) = 0.67	0.2<br>0.13
		Model 4= 0.71<br>Model 4 (Gleason score≥7) = 0.672	0.21<br>0.13

Sensitivity and specificity data are presented in Table 4. At 95% sensitivity, the Babaian et al, model shows the highest specificity (51%) whereas the Jansen model for both sites had the lowest specificity (~23.5%). In the Hill study, with a sensitivity of ~90%, the specificity was lower than in other studies (~18% and 28%) for method 1 and 2 respectively. In the study reported by Lazzeri, the sensitivity and specificity were not reported for the overall model; instead their study reports sensitivity and specificity for predictive variables individually. The highest sensitivity (90.5%) of %p2PSA and %fPSA achieved the highest specificity in predicting PCa at 21.5% and 22.8% respectively. Percentage p2PSA and PHI were more associated with Gleason scores.

Table 4: Sensitivity and specificity profile at different levels for each model.

Study	Sensitivity	Specificity	Probability	Positive	Negative

For peer review only

			cut-off	predictive value	predictive value
Carlson [61]	99	18	>15	≤47	N/A
	95	34	18	51	NA
	89	43	20	42	NA
Babaian [62]	95	51	NA	39	97
	92	62	NA	44	96
	89	62	NA	43	95
Jansen [63] *(Site 1)*	95	23.9	NA	NA	NA
Jansen [63] *(Site 1)*	90	30.1	NA	NA	NA
Jansen [63] *(Site 2)*	95	23.2	NA	NA	NA
Jansen [63] *(Site 2)*	90	36.2	NA	NA	NA
Hill [64] *(Method 1)*	90.9	17.6	33	47.1	70.5
Hill [64] *(Method 2)*	89.8	28	13	20.6	91.3
Hill [64] *(Method 1)*	80.5	37.1	37	50.9	70.2
Hill [64] *(Method 2)*	78.2	45	15	28.7	88.8
Hill [64] *(Method 1)*	39.9	81.4	48	63.4	62.6
Hill [64] *(Method 2)*	45.8	79.5	23	36.7	85

* Lazzeri [65] model reported only sensitivity and specificity for predictive variables individually and at sensitivity of 90, %p2PSA and %fPSA achieved the highest specificity

For peer review only

Table 5 summarises the validation and calibration results for the studies included. Model calibration was reported in two studies [61 65]. Carlson plotted the observed and expected risks using calibration plots, whereas Lazzeri used the Hosmer-Lemeshow goodness-of-fit test. In terms of validation, two studies did not report model validation [63 64]. Only one study reported an external validation using an additional data set consisting of 525 patients [61]. Cross-validation using multiple re-sampling schemes was used in the Babaian Study, however, they did not report the number of time this was performed [62]. Lazzeri used 200 bootstrap re-samples to minimise overfitting bias [65].

Table 5: Validation and calibration for included models.

Author & Year	Validation	Calibration
Carlson,1998 [61]	External validation on additional data set consisting of 525 patients	Calibration plot
Babaian,2000 [62]	Cross-validation and separate data set of 151	N/A
Jansen, 2010 [63]	N/A	N/A
Hill, 2013 [64]	N/A	N/A
Lazzeri, 2013 [65]	Internal validation using 200 bootstrap resamples	Internal calibration using the Hosmer-Lemeshow goodness-of-fit test

Study quality assessment

Quality assessment was carried out by two reviewers (MA and AL) with any discordance resolved by a third reviewer (KM). The assessment of results suggested some issue of study quality according to the criteria as set in the PROBAST tool, particularly in the analysis domain. For instance, one study applied univariable analysis to select predictors [61]. Three studies did not measure calibration [62-64]. Furthermore, two studies did not account for optimism and overfitting by using internal validation methods [63 64]. Whereas one study did not use appropriate measures for model performance i.e. AUC, this study reported the calibration [61].

The event per variable (EPV) was lower than recommended (< 10) [59 66] in the Babaian [62] study indicating inadequate power. Four studies did not report missing data or how they handled it [61-63 65]. The remaining study used complete-case analysis and excluded subjects with missing data on laboratory biomarkers (n=75) [64]. The PROBAST guidelines state that in a prediction model study where any risk of bias and applicability is low in all four domains, a regrading to high risk of bias should be considered when the study did not validate the model externally [60]. Thus, although the quality assessment for the Lazzeri

For peer review only

study [65] was graded low risk in all the four domains, since the study did not report any external validation of the model, the assessment of the study has been regraded to high risk of bias according to the PROBAST criteria. A full quality assessment for all studies is presented in Table 6.

For peer review only

Table 6: Quality assessment for risk of bias and applicability concern for included studies.

Study	ROB*				Applicability			Overall	
	Participants	Predictors	Outcome	Analysis	Participants	Predictors	Outcome	ROB	Applicability
Carlson [61]	+	+	-	-	+	+	-	-	-
Babaian [62]	+	+	+	-	-	+	+	-	-
Jansen [63]	-	-	-	-	+	-	-	-	-
Hill [64]	-	+	+	-	-	+	+	-	-
Lazzeri [65]	+	+	+	+	+	+	+	-	+

* ROB- Risk of bias
+ indicates a low risk of bias or applicability; - indicates a high risk of bias or applicability.

For peer review only

DISCUSSION

Despite the large number of PCa risk prediction models, the majority still include clinical inputs and/or more costly biomarker or genetic panels; few low cost models exist that do not include specialist clinical input or more expensive further testing that limits there use for population wide assessments. To our knowledge, this is the first study to examine risk prediction models for PCa that are low cost and do not include clinical and genetic variables and are based on single time point assessment.

Our study identified five unique models that met the set criteria. The Carlson model [61] has the largest population (3,773 subjects) when compared to the other four studies. Although they reported an 11% increase in specificity, they did not report AUC predictive estimates. It has been acknowledged that sensitivity and specificity results are dependent on the prevalence of the disease. Hence the comparison between populations where the PCa prevalence may vary (especially in early detection) will be difficult [33]. More importantly, by not reporting the AUC estimate, the model raises some doubts regarding the reliability of the model and its implementation [33]. It will also make comparison to other models not applicable [67].

Babaian [62] developed an algorithm and compared the performance of the ANN to PSA density (total PSA divided by prostate volume) (PSAD), %fPSA, and transition zone density (PSAD-TZ). Their ANN demonstrated a significant increase of model specificity that reached 51% when sensitivity was held at 95%. This was better than the specificity value of each individual variable such as %fPSA (10%), PSAD (39%), and PSAD-TZ (22%). In terms of AUC, the ANN achieved a moderate accuracy (0.74) being the second highest among all studies included. However, the ANN model did not show significant improvement when compared with a model fitted with only individual variables (AUC for %fPSA= 0.64, PSAD= 0.74, PSAD-TZ= 0.75). They included a number of uncommon pre-biopsy inputs into their algorithm such as prostatic acid phosphatase and creatinine kinase [68]. Furthermore, they used a tight PSA range (2.5-4.0ng/ml) which meant that their model may be less suitable for patients with PSA level below or above that range, thus limiting its generalisability.

For peer review only

The study by Jansen and colleagues [63] demonstrated that adding p2PSA to the base model of PSA and fPSA significantly enhanced the PCa predictive value and specificity. The association and added value of p2PSA in the prediction and detection of PCa have been reported by several other studies [16 69-72]. Jansen [63] showed that p2PSA has no clear association with aggressive PCa. However, the base model that includes p2PSA had the highest clinical significance in correlation to pathologic Gleason score with a p-value of 0.008 compared to %fPSA and PHI (p-value 0.01 and 0.02) respectively. Although they used archived blood samples and retrospective analysis, the results were similar to a prospective study of 268 patients [16].

Hill [64] used a case-control study to evaluate several laboratory biomarkers. They found HGB, RBC, haematuria, creatinine, PSA, Age, MCV, and ethnicity ("being black") were statistically significantly associated in the first method ($p < 0.05$). In the second method, HGB, RBC, creatinine, PSA, age, and MCV were found to be statistically significantly correlated ($p <0.001$) with PCa. However, since this study was designed as a case-control study, it would have been more prone to uncontrolled confounding and selection bias. Moreover, the type of screening protocols used in Veterans' Administrations may vary to those conducted in other healthcare systems; therefore, the results may not be applicable to other populations. Furthermore, subjects with a PSA level < 4.0 ng/ml have not been investigated, and thus, the performance of the models are unknown for individuals in this group.

Lazzeri et al, [65] in a European multi-centre study have evaluated similar biomarkers as in Jansen study with the same PSA range 2-10 ng/ml prospectively. They found no difference in both %p2PSA and PHI as individual PCa predictors with AUC of 0.67 (95% CI 0.64-0.71). However, the base model (consisting of PSA, fPSA, and %fPSA) that also included either p2PSA or PHI out-performed the base model alone and the base model including %p2PSA. In the analysis, the additive value of both p2PSA and PHI is 0.064 and 0.056 for %p2PSA for predicting the risk of PCa. These additive values increased to 0.076 for both p2PSA and PHI and 0.073 for %p2PSA in predicting Gleason scores ≥7 for the disease. The usefulness of PHI in improving the predictive accuracy of PCa over total and free PSA has been confirmed and reported by several studies [16 72-75].

For peer review only

In general, only one study has validated their model externally [61], whereas the remaining studies were either validated internally [62 65] or not reported any validation methods [63 64]. Prediction models may not be equally applicable to all data sets as patients' characteristics may vary [20 76]. As a result, the generalisability of a model might be poor when it used in populations other than that used in building the model. Therefore, external validation should be conducted before applying any new model into general practice [77 78].

Another key performance measure of any model that needs careful evaluation is calibration. A calibration plot with a calibration slope is more preferable than the Hosmer-Lemeshow test; it has been acknowledged that evaluating a good and well calibrated model based on a large dataset can still fail the Hosmer-Lemeshow test. In contrast, when evaluating a poorly calibrated model with a small dataset it can still pass the Hosmer-Lemeshow test [79]. In our analysis, three studies fail to report the calibration of the model [62-64] whilst the Carlson study [61] used a calibration plot and Lazzeri [65] used the Hosmer-Lemeshow test. Excluding calibration from the majority of models may explain why some models are not currently used in practice [79].

With regard to biopsy cores, only two studies used extended biopsy cores. Babaian [62] used an 11-core multi-site biopsy, whereas Lazzeri [65] used at least 12 biopsy cores. Moreover, two studies used six cores biopsy in their model [61 63]. The use of six-core biopsy has been criticized as not being adequate in detecting PCa [80] and that models developed using sextant biopsy are less accurate than when a 10-core biopsy is used [81]. As a result, the European guideline for clinical PCa recommended an extended biopsy as standard practice for PCa detection [82].

It is worth noting that all five reviewed models performed better than just PSA alone. However, none of them has both high specificity and sensitivity. The level of sensitivity has been increased, and despite enhancement in the specificity, it is still considered low. Specificity is crucial when it comes to being used in a population setting as men without PCa should be ruled out as much as possible from further invasive engagement with the health system.

Our review therefore suggests that none of the reviewed models provide an ideal performance in predicting PCa with high sensitivity and high specificity. It is particularly

For peer review only - http://bmjopen.bmj.com/site/about/guidelines.xhtml

important when considering the application of PCa risk prediction at the population level that the tool used should be able to both detect the outcome and filter out people with no disease. As there is robust evidence suggesting that the clinical relevance of PSA range to the detection of PCa differs across age groups [83-85], any future model should consider PSA threshold in relation to a specific age range. Risk prediction models for PCa should therefore take account of age.

Out of the five reviewed models, the Lazzeri model 2, has the greatest potential to be implemented in primary care. It achieved the least risk of bias and had fair discrimination for both any and aggressive PCa. It also had the largest improvement in discrimination performance compared to PSA alone. Moreover, except for the p2PSA that requires a specific assay, the included variables are common and easy to measure. However, before it could be used, the model requires to be validated externally.

Comparison with other studies

To our knowledge, three systematic reviews of PCa prediction tools have been published [20 26 27]. In the Louie et al. review, risk models were included that were externally validated in at least five study populations for the purpose of meta-analysis and only six studies were included in their analysis. Furthermore, all the studies included incorporated clinical tests such as DRE and/or TRUS-PV [26]. Schroder and Kattan [20] reviewed models that were built to predict the likelihood of having a positive prostate biopsy for cancer. However, it appears that they also included models where subjects had a previous negative biopsy. As such, some of the models included variables related to biopsy results and cores. The review by Shariat and colleagues examined different types of predictive tools [27]. They explored tools that predict PCa on initial and repeat biopsy, pathologic stages, biochemical recurrence after radical proctectomy, metastasis, survival, and life expectancy. Similarly, virtually all of the prediction tools that were based on initial biopsy included variables based on invasive procedures.

Strengths and limitations of this study

This report is the first to review risk prediction tools for PCa that can be used in primary care and community settings. Any prediction model should therefore be simple to use, based on non-invasive tests, be feasible at a population level and at low cost. We carried

For peer review only

out an extensive data extraction relating to important features and characteristics for each study included, such as modelling method, source of data, sample size, variables, discrimination, validation, and cancer detection rate. We have also followed PRISMA guidelines for identifying eligible articles as well as for reporting this study. In addition, the PROBAST tool was adopted to assess the quality and risk of bias for each prediction model.

Our study has some limitations. Our aim was to identify prediction models that have the potential to be implemented in a primary care or community settings, and consequently our search strategy was to retrieve relevant studies for this specific purpose. Furthermore, we excluded articles that were not published in English or did not have an abstract. Moreover, only two databases were searched, besides manual search, to retrieve original studies.

A previous systematic review suggested that the majority of relevant studies could be identified through a manual search of articles reference lists instead of a database alone [20]. We identified four eligible studies using this approach. Given the small number of models identified by the approach we followed, that can be applied in primary care settings compared to the large number relating to wider existing models, it is unlikely that we have not included any study that would affect the results of our review.

Implications and future research

It is now accepted that the PSA test and its derivatives have some limitations for detecting PCa as defined by subsequent biopsy [86]. As a consequence, a considerable number of PCa prediction models have been built to improve prediction accuracy. This has resulted in a plethora of PCa risk prediction tools, with to date more than 100 models described [87 88]. There is evidence that some of these models show benefit and have better performance over just PSA measurement alone [20]. It also has been demonstrated that some of these models out-performed clinical experts in predicting PCa [28 29]. Although such models are not designed to replace specialist clinical judgment or patient preferences [76 86 89], they can help in patient counselling and aid clinicians to decide whether a prostate biopsy should be taken or not [77 89 90].

For peer review only

Given the small number of risk prediction models for PCa that do not incorporate clinical or genetic tests, the discrimination of these reviewed models ranged between poor to moderate (AUC range ~0.65 to ~0.75); in addition there were some issues relating to their study design and analysis raises the risk of bias. Consequently none of these models could be currently recommended for use in a primary care and community health care setting. Several guidelines are against using PSA test based screening for PCa; the US Preventive Services task force, the Canadian task force on preventive health and the American College of Preventive Medicine do not currently recommend PSA-based testing due to insufficient evidence [91-93]. This has made it difficult, so far, to convince policymakers to adopt PCa screening programme.

The first guideline of PROSTATE CANCER UK states *"In the future, health professionals should look at a man's PSA level alongside other known risk factors as part of a risk assessment tool, when one becomes available."* [94] However, the vast majority of the current PCa risk prediction models are not suitable for routine use as they include clinical and genetic tests and are not validated externally in other cohorts. Therefore, the main challenge in the UK, remains to develop a risk prediction tool that is reliable, cheap, is applicable for as wide an ethnicity as possible, and most importantly is easy to use and can be implemented at a primary care level [95].

The value of such risk tools is that they will help to stratify men at high risk of developing PCa earlier so that they have appropriate management and/or surveillance programme as early as possible and therefore, may fit into the clinical pathway. Such tools should help physicians have a better understanding of the risk for this disease and simplify the procedures and discussions with patients when recommending further specialist-led investigations such as DRE and/or MRI where a decision on whether a biopsy should or not perform is concluded. Furthermore, using the appropriate risk prediction tool will avoid men from undergoing inappropriate further and frequent testing [95]. This will reduce any associated costs of inappropriate tests and decrease the burden on health care delivery systems.

It is crucial to address these issues by identify all possible risk factors for PCa that are non-clinical, non-genetic, and easy to use and interpret. There remains a pressing need

For peer review only

to develop a risk prediction tool in the future using all appropriate factors (potentially also including genetics once there is infrastructure in place for genetic testing in the primary care and the cost comes down) into a robust multivariable analysis and validate the model externally to eliminate applicability and generalisability concerns. Only when this is achieved will it be possible to introduce a prostate cancer screening programme fit for purpose.

CONCLUSION

There is a paucity of suitable low cost risk models that incorporate non-clinical, non-genetic inputs and which can be used at a primary care level and in other community health services. Existing models have limitations reflecting both study design and reporting performance measures. Future research should take into account these key issues and explore other risk factors for incorporation into further models.

ACKNOWLEDGEMENT

MA is supported by Kuwait's Ministry of Health.

AUTHOR CONTRIBUTIONS

MA, AL and KM were involved in study conception, idea and design. Data acquisition and extraction was obtained by MA. Data synthesis and interpretation was carried out by MA, AL and WO. MA and AL drafted the manuscript. All authors approved the final version of manuscript. KM is the study guarantor.

FUNDING

KM and AL are supported by the NIHR Manchester Biomedical Research Centre and by the ICEP ("This work was also supported by CRUK [grant number C18281/A19169]). KM is also supported by the Allan Turing Institute.

COMPETING INTERESTS

All authors have completed the ICMJE uniform disclosure form at www.icmje.org/coi_disclosure.pdf and declare: no support from any organisation for the submitted work; no financial relationships with any organisations that might have an interest in the submitted work in the previous three years; no other relationships or activities that could appear to have influenced the submitted work.

For peer review only

ETHICAL APPROVAL

Not applicable as this study did not involve any personal data.

For peer review only

DATA SHARING

Data extraction sheet and the PROBAST assessment form for risk of bias is available upon request.

Figure legend

Figure 1: Flow diagram of studies included using PRISMA method

REFERENCES

1. Bray F, Ferlay J, Soerjomataram I, et al. Global cancer statistics 2018: GLOBOCAN estimates of incidence and mortality worldwide for 36 cancers in 185 countries. *CA: a cancer journal for clinicians* 2018;68(6):394-424.
2. Cancer Research UK. Prostate cancer statistics 2017 [Available from: https://www.cancerresearchuk.org/health-professional/cancer-statistics/statistics-by-cancer-type/prostate-cancer#heading-One.
3. Center MM, Jemal A, Lortet-Tieulent J, et al. International variation in prostate cancer incidence and mortality rates. *European urology* 2012;61(6):1079-92.
4. Zhou CK, Check DP, Lortet-Tieulent J, et al. Prostate cancer incidence in 43 populations worldwide: An analysis of time trends overall and by age group. *Int J Cancer* 2016;138(6):1388-400.
5. Dijkstra S, Mulders P, Schalken J. Clinical use of novel urine and blood based prostate cancer biomarkers: a review. *Clin Biochem* 2014;47(10-11):889-96.
6. Fontanella P, Benecchi L, Grasso A, et al. Decision-making tools in prostate cancer: from risk grouping to nomograms. *Minerva Urol Nefrol* 2017;69(6):556-66.
7. Stephan C, Ralla B, Jung K. Prostate-specific antigen and other serum and urine markers in prostate cancer. *Biochim Biophys Acta* 2014;1846(1):99-112.
8. Thompson IM, Pauler DK, Goodman PJ, et al. Prevalence of prostate cancer among men with a prostate-specific antigen level≤ 4.0 ng per milliliter. *N Engl J Med* 2004;350(22):2239-46.
9. Pereira-Azevedo N, Verbeek JFM, Nieboer D, et al. Head-to-head comparison of prostate cancer risk calculators predicting biopsy outcome. *Transl Androl Urol* 2018;7(1):18-26.
10. Stephan C, Cammann H, Meyer H-A, et al. PSA and new biomarkers within multivariate models to improve early detection of prostate cancer. *Cancer lett* 2007;249(1):18-29.
11. Polascik TJ, Oesterling JE, Partin AW. Prostate specific antigen: a decade of discovery-what we have learned and where we are going. *J Urol* 1999;162(2):293-306.
12. Stamey TA, Caldwell M, McNEAL JE, et al. The prostate specific antigen era in the United States is over for prostate cancer: what happened in the last 20 years? *J Urol* 2004;172(4 Pt 1):1297-301.

For peer review only

13. Bunting PS. A guide to the interpretation of serum prostate specific antigen levels. *Clin Biochem* 1995;28(3):221-41.

14. Catalona WJ, Partin AW, Sanda MG, et al. A multi-center study of [− 2] pro-prostate-specific antigen (PSA) in combination with PSA and free PSA for prostate cancer detection in the 2.0 to 10.0 ng/mL PSA range. *J Urol* 2011;185(5):1650-55.

15. Mistry K, Cable G. Meta-analysis of prostate-specific antigen and digital rectal examination as screening tests for prostate carcinoma. *The Journal of the American Board of Family Practice* 2003;16(2):95-101. [published Online First: 2003/04/01]

16. Guazzoni G, Nava L, Lazzeri M, et al. Prostate-specific antigen (PSA) isoform p2PSA significantly improves the prediction of prostate cancer at initial extended prostate biopsies in patients with total PSA between 2.0 and 10 ng/ml: results of a prospective study in a clinical setting. *Eur Urol* 2011;60(2):214-22.

17. Heijnsdijk EA, der Kinderen A, Wever EM, et al. Overdetection, overtreatment and costs in prostate-specific antigen screening for prostate cancer. *British journal of cancer* 2009;101(11):1833-8. doi: 10.1038/sj.bjc.6605422 [published Online First: 2009/11/12]

18. Roobol MJ. Is prostate cancer screening bad or good? Summary of a debate at the innovation in urology meeting, September 17-19, 2010, Milan, Italy. *Eur Urol* 2011;59(3):359-62. doi: 10.1016/j.eururo.2010.10.036 [published Online First: 2010/11/03]

19. Mottet N, Bellmunt J, Bolla M, et al. EAU-ESTRO-SIOG Guidelines on Prostate Cancer. Part 1: Screening, Diagnosis, and Local Treatment with Curative Intent. *Eur Urol* 2017;71(4):618-29. doi: 10.1016/j.eururo.2016.08.003 [published Online First: 2016/08/30]

20. Schroder F, Kattan MW. The comparability of models for predicting the risk of a positive prostate biopsy with prostate-specific antigen alone: a systematic review. *Eur Urol* 2008;54(2):274-90.

21. Ross PL, Scardino PT, Kattan MW. A catalog of prostate cancer nomograms. *J Urol* 2001;165(5):1562-8. [published Online First: 2001/05/09]

22. Shariat SF, Karakiewicz PI, Suardi N, et al. Comparison of nomograms with other methods for predicting outcomes in prostate cancer: a critical analysis of the literature. *Clinical cancer research : an official journal of the American Association for Cancer Research* 2008;14(14):4400-7. doi: 10.1158/1078-0432.Ccr-07-4713 [published Online First: 2008/07/17]

23. Hu X, Cammann H, Meyer HA, et al. Artificial neural networks and prostate cancer--tools for diagnosis and management. *Nature reviews Urology* 2013;10(3):174-82. doi: 10.1038/nrurol.2013.9 [published Online First: 2013/02/13]

24. Caras RJ, Sterbis JR. Prostate cancer nomograms: a review of their use in cancer detection and treatment. *Current urology reports* 2014;15(3):391. doi: 10.1007/s11934-013-0391-0 [published Online First: 2014/01/24]

25. Iremashvili V, Soloway MS, Pelaez L, et al. Comparative validation of nomograms predicting clinically insignificant prostate cancer. *Urology* 2013;81(6):1202-8. doi: 10.1016/j.urology.2013.01.062 [published Online First: 2013/04/09]

For peer review only

26. Louie KS, Seigneurin A, Cathcart P, et al. Do prostate cancer risk models improve the predictive accuracy of PSA screening? A meta-analysis. *Annals of Oncology* 2015;26:848-64. doi: 10.1093/annonc/mdu525

27. Shariat SF, Kattan MW, Vickers AJ, et al. Critical review of prostate cancer predictive tools. *Future oncology (London, England)* 2009;5(10):1555-84. doi: 10.2217/fon.09.121 [published Online First: 2009/12/17]

28. Ross PL, Gerigk C, Gonen M, et al. Comparisons of nomograms and urologists' predictions in prostate cancer. *Seminars in urologic oncology* 2002;20(2):82-8. [published Online First: 2002/05/16]

29. Walz J, Gallina A, Perrotte P, et al. Clinicians are poor raters of life-expectancy before radical prostatectomy or definitive radiotherapy for localized prostate cancer. *BJU Int* 2007;100(6):1254-8.

30. Ouzaid I, Yates DR, Hupertan V, et al. A direct comparison of the diagnostic accuracy of three prostate cancer nomograms designed to predict the likelihood of a positive initial transrectal biopsy. *The Prostate* 2012;72(11):1200-6. doi: 10.1002/pros.22470 [published Online First: 2012/01/04]

31. Eastham JA, May R, Robertson JL, et al. Development of a nomogram that predicts the probability of a positive prostate biopsy in men with an abnormal digital rectal examination and a prostate-specific antigen between 0 and 4 ng/mL. *Urology* 1999;54(4):709-13.

32. Garzotto M, Hudson RG, Peters L, et al. Predictive modeling for the presence of prostate carcinoma using clinical, laboratory, and ultrasound parameters in patients with prostate specific antigen levels < or = 10 ng/mL. *Cancer* 2003;98(7):1417-22. doi: 10.1002/cncr.11668 [published Online First: 2003/09/26]

33. Karakiewicz PI, Benayoun S, Kattan MW, et al. Development and validation of a nomogram predicting the outcome of prostate biopsy based on patient age, digital rectal examination and serum prostate specific antigen. *J Urol* 2005;173(6):1930-4.

34. Kranse R, Roobol M, Schroder FH. A graphical device to represent the outcomes of a logistic regression analysis. *The Prostate* 2008;68(15):1674-80. doi: 10.1002/pros.20840 [published Online First: 2008/08/21]

35. Thompson IM, Ankerst DP, Chi C, et al. Assessing prostate cancer risk: results from the Prostate Cancer Prevention Trial. *J Natl Cancer Inst* 2006;98(8):529-34.

36. Schröder FH, Kruger AB, Rietbergen J, et al. Evaluation of the digital rectal examination as a screening test for prostate cancer. *Journal of the National Cancer Institute* 1998;90(23):1817-23.

37. Vis AN, Hoedemaeker RF, Roobol M, et al. Tumor characteristics in screening for prostate cancer with and without rectal examination as an initial screening test at low PSA (0.0–3.9 ng/ml). *The Prostate* 2001;47(4):252-61.

38. Smith DS, Catalona WJ. Interexaminer variability of digital rectal examination in detecting prostate cancer. *Urology* 1995;45(1):70-74.

For peer review only

39. Barocas DA, Cowan JE, Smith JA, Jr., et al. What percentage of patients with newly diagnosed carcinoma of the prostate are candidates for surveillance? An analysis of the CaPSURE database. 2008

40. Vis A, Kranse R, Roobol M, et al. Serendipity in detecting disease in low prostate-specific antigen ranges. *BJU international* 2002;89(4):384-89.

41. Littrup PJ, Bailey SE. Prostate cancer: the role of transrectal ultrasound and its impact on cancer detection and management. *Radiologic Clinics of North America* 2000;38(1):87-113.

42. Gerber GS, Chodak GW. Routine screening for cancer of the prostate. *JNCI: Journal of the National Cancer Institute* 1991;83(5):329-35.

43. Gelfand DE, Parzuchowski J, Cort M, et al. Digital rectal examinations and prostate cancer screening: attitudes of African American men. *Oncology Nursing Forum-Oncology Nursing Society* 1995;22(8):1253-58.

44. Myers RE, Wolf TA, McKee L, et al. Factors associated with intention to undergo annual prostate cancer screening among African American men in Philadelphia. *Cancer: Interdisciplinary International Journal of the American Cancer Society* 1996;78(3):471-79.

45. Consedine NS, Horton D, Ungar T, et al. Fear, knowledge, and efficacy beliefs differentially predict the frequency of digital rectal examination versus prostate specific antigen screening in ethnically diverse samples of older men. *American journal of men's health* 2007;1(1):29-43.

46. Consedine NS, Morgenstern AH, Kudadjie-Gyamfi E, et al. Prostate cancer screening behavior in men from seven ethnic groups: the fear factor. *Cancer Epidemiology and Prevention Biomarkers* 2006;15(2):228-37.

47. Ukoli FA, Patel K, Hargreaves M, et al. A tailored prostate cancer education intervention for low-income African Americans: Impact on knowledge and screening. *Journal of health care for the poor and underserved* 2013;24(1):311-31.

48. Ogunsanya ME, Brown CM, Odedina FT, et al. Beliefs regarding prostate cancer screening among black males aged 18 to 40 Years. *American journal of men's health* 2017;11(1):41-53.

49. Clarke-Tasker VA. What we thought we knew: African American males' perceptions of prostate cancer and screening methods. *ABNF Journal* 2002;13(3):56.

50. Romero FR, Romero AW, Brenny Filho T, et al. Patients' perceptions of pain and discomfort during digital rectal exam for prostate cancer screening. *Archivos espanoles de urologia* 2008;61(7):850-54.

51. Essink-Bot M-L, de Koning HJ, Nijs HG, et al. Short-term effects of population-based screening for prostate cancer on health-related quality of life. *Journal of National Cancer Institute* 1998;90(12):925-31.

52. Appleton L, Wyatt D, Perkins E, et al. The impact of prostate cancer on men's everyday life. *European journal of cancer care* 2015;24(1):71-84.

For peer review only

53. Lee DJ, Consedine NS, Spencer BA. Barriers and facilitators to digital rectal examination screening among African-American and African-Caribbean men. *Urology* 2011;77(4):891-98.

54. Nagler HM, Gerber EW, Homel P, et al. Digital rectal examination is barrier to population-based prostate cancer screening. *Urology* 2005;65(6):1137-40.

55. Moher D, Liberati A, Tetzlaff J, et al. Preferred reporting items for systematic reviews and meta-analyses: the PRISMA statement. *PLoS medicine* 2009;6(7):e1000097.

56. Park SH, Goo JM, Jo CH. Receiver operating characteristic (ROC) curve: practical review for radiologists. *Korean journal of radiology* 2004;5(1):11-8. doi: 10.3348/kjr.2004.5.1.11 [published Online First: 2004/04/06]

57. Han K, Song K, Choi BW. How to Develop, Validate, and Compare Clinical Prediction Models Involving Radiological Parameters: Study Design and Statistical Methods. *Korean journal of radiology* 2016;17(3):339-50. doi: 10.3348/kjr.2016.17.3.339 [published Online First: 2016/05/03]

58. Steyerberg EW. Clinical prediction models: a practical approach to development, validation, and updating: Springer Science & Business Media 2008.

59. Pavlou M, Ambler G, Seaman SR, et al. How to develop a more accurate risk prediction model when there are few events. *Bmj* 2015;351:h3868.

60. Wolff RF, Moons KGM, Riley RD, et al. PROBAST: A Tool to Assess the Risk of Bias and Applicability of Prediction Model Studies. *Ann Intern Med* 2019;170(1):51-58.

61. Carlson GD, Calvanese CB, Partin AW. An algorithm combining age, total prostate-specific antigen (PSA), and percent free PSA to predict prostate cancer: results on 4298 cases. *Urology* 1998;52(3):455-61.

62. Babaian RJ, Fritsche H, Ayala A, et al. Performance of a neural network in detecting prostate cancer in the prostate-specific antigen reflex range of 2.5 to 4.0 ng/mL. *Urology* 2000;56(6):1000-6.

63. Jansen FH, van Schaik RH, Kurstjens J, et al. Prostate-specific antigen (PSA) isoform p2PSA in combination with total PSA and free PSA improves diagnostic accuracy in prostate cancer detection. *European urology* 2010;57(6):921-27.

64. Hill OT, Mason TJ, Schwartz SW, et al. Improving prostate cancer detection in veterans through the development of a clinical decision rule for prostate biopsy. *BMC Urol* 2013;13:6.

65. Lazzeri M, Haese A, de la Taille A, et al. Serum isoform [-2]proPSA derivatives significantly improve prediction of prostate cancer at initial biopsy in a total PSA range of 2-10 ng/ml: a multicentric European study. *Eur Urol* 2013;63(6):986-94.

66. Moons KG, de Groot JA, Bouwmeester W, et al. Critical appraisal and data extraction for systematic reviews of prediction modelling studies: the CHARMS checklist. *PLoS medicine* 2014;11(10)

67. Porter CR, O'Donnell C, Crawford ED, et al. Predicting the outcome of prostate biopsy in a racially diverse population: a prospective study. *Urology* 2002;60(5):831-35.

For peer review only

68. Porter CR, Gamito EJ, Crawford ED, et al. Model to predict prostate biopsy outcome in large screening population with independent validation in referral setting. *Urology* 2005;65(5):937-41.

69. Catalona WJ, Bartsch G, Rittenhouse HG, et al. Serum pro-prostate specific antigen preferentially detects aggressive prostate cancers in men with 2 to 4 ng/ml prostate specific antigen. *J Urol* 2004;171(6 Pt 1):2239-44.

70. Sokoll LJ, Sanda MG, Feng Z, et al. A prospective, multicenter, National Cancer Institute Early Detection Research Network study of [-2]proPSA: improving prostate cancer detection and correlating with cancer aggressiveness. *Cancer Epidemiol Biomarkers Prev* 2010;19(5):1193-200.

71. Sokoll LJ, Wang Y, Feng Z, et al. [-2]proenzyme prostate specific antigen for prostate cancer detection: a national cancer institute early detection research network validation study. *J Urol* 2008;180(2):539-43; discussion 43.

72. Stephan C, Vincendeau S, Houlgatte A, et al. Multicenter evaluation of [-2]proprostate-specific antigen and the prostate health index for detecting prostate cancer. *Clin Chem* 2013;59(1):306-14.

73. Le BV, Griffin CR, Loeb S, et al. [-2]Proenzyme prostate specific antigen is more accurate than total and free prostate specific antigen in differentiating prostate cancer from benign disease in a prospective prostate cancer screening study. *J Urol* 2010;183(4):1355-9.

74. Loeb S, Sokoll LJ, Broyles DL, et al. Prospective multicenter evaluation of the Beckman Coulter Prostate Health Index using WHO calibration. *J Urol* 2013;189(5):1702-06.

75. Lughezzani G, Lazzeri M, Haese A, et al. Multicenter European external validation of a Prostate Health Index–based nomogram for predicting prostate cancer at extended biopsy. *Eur Urol* 2014;66(5):906-12.

76. Chun FK-H, Briganti A, Graefen M, et al. Development and external validation of an extended 10-core biopsy nomogram. *European urology* 2007;52:436-44.

77. Herman MP, Dorsey P, John M, et al. Techniques and predictive models to improve prostate cancer detection. *Cancer* 2009;115(S13):3085-99.

78. Trottier G, Roobol MJ, Lawrentschuk N, et al. Comparison of risk calculators from the Prostate Cancer Prevention Trial and the European Randomized Study of Screening for Prostate Cancer in a contemporary Canadian cohort. *BJU Int* 2011;108(8 Pt 2):E237-44.

79. Collins GS, de Groot JA, Dutton S, et al. External validation of multivariable prediction models: a systematic review of methodological conduct and reporting. *BMC Med Res Methodol* 2014;14(1):40.

80. Miyake H, Kurahashi T, Takenaka A, et al. Improved accuracy for predicting the Gleason score of prostate cancer by increasing the number of transrectal biopsy cores. *Urol Int* 2007;79(4):302-6.

81. Chun FK, Briganti A, Graefen M, et al. Development and external validation of an extended 10-core biopsy nomogram. *Eur Urol* 2007;52(2):436-44.

For peer review only

82. Aus G, Abbou CC, Bolla M, et al. EAU guidelines on prostate cancer. *Eur Urol* 2005;48(4):546-51.

83. Vickers AJ, Ulmert D, Serio AM, et al. The predictive value of prostate cancer biomarkers depends on age and time to diagnosis: towards a biologically-based screening strategy. *Int J Cancer* 2007;121(10):2212-7.

84. Steuber T, O'Brien MF, Lilja H. Serum markers for prostate cancer: a rational approach to the literature. *Eur Urol* 2008;54(1):31-40.

85. Carlsson S, Assel M, Sjoberg D, et al. Influence of blood prostate specific antigen levels at age 60 on benefits and harms of prostate cancer screening: population based cohort study. *Bmj* 2014;348:g2296.

86. Cavadas V, Osorio L, Sabell F, et al. Prostate cancer prevention trial and European randomized study of screening for prostate cancer risk calculators: a performance comparison in a contemporary screened cohort. *Eur Urol* 2010;58(4):551-8.

87. Vickers AJ. Prediction models: revolutionary in principle, but do they do more good than harm. *J Clin Oncol* 2011;29(22):2951-52.

88. Shariat SF, Karakiewicz PI, Margulis V, et al. Inventory of prostate cancer predictive tools. *Curr Opin Urol* 2008;18(3):279-96.

89. Kawakami S, Numao N, Okubo Y, et al. Development, validation, and head-to-head comparison of logistic regression-based nomograms and artificial neural network models predicting prostate cancer on initial extended biopsy. *Eur Urol* 2008;54(3):601-11.

90. Hernandez DJ, Han M, Humphreys EB, et al. Predicting the outcome of prostate biopsy: comparison of a novel logistic regression-based model, the prostate cancer risk calculator, and prostate-specific antigen level alone. *BJU Int* 2009;103(5):609-14.

91. Moyer VA. Screening for prostate cancer: US Preventive Services Task Force recommendation statement. *Ann Intern Med* 2012;157(2):120-34.

92. Bell N, Connor Gorber S, Shane A, et al. Recommendations on screening for prostate cancer with the prostate-specific antigen test. *CMAJ* 2014;186(16):1225-34.

93. Lim LS, Sherin K, Committee APP. Screening for prostate cancer in US men: ACPM position statement on preventive practice. *Am J Prev Med* 2008;34(2):164-70.

94. Prostate Cancer UK. Consensus statements on PSA testing in asymptomatic men in the UK - information for health professionals 2016 [Available from: https://prostatecanceruk.org/about-us/projects-and-policies/consensus-on-psa-testing accessed 20-8 2019.

95. Frame I, Cant S. Current challenges in prostate cancer: an interview with Prostate Cancer UK. *BMC Med* 2015;13(1):166.

For peer review only

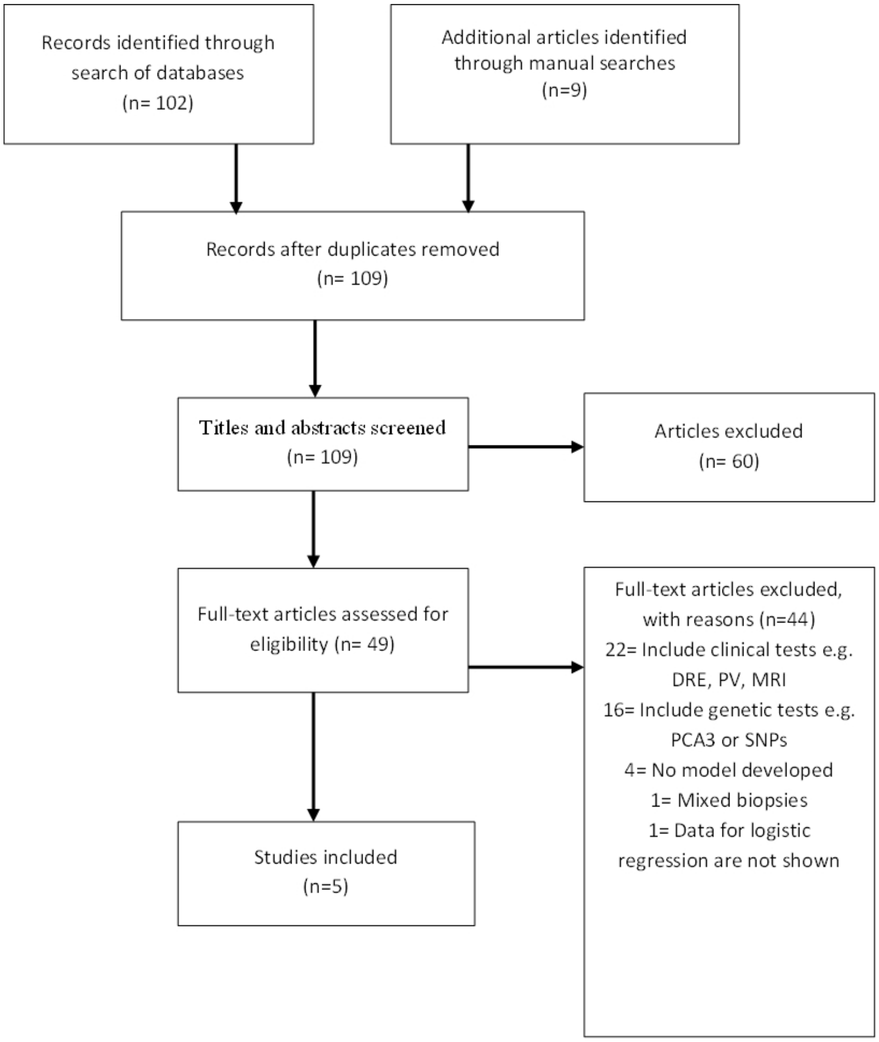

Flow diagram of studies included using PRISMA method

78x91mm (300 x 300 DPI)

Search Strategy

1) * Prostatic Neoplasms/
2) Initial biopsy.mp
3) First biopsy.mp
4) * “Early Detection of Cancer”/mt [Methods]
5) 2 or 3 or 4
6) 1 and 5
7) Nomograms/
8) “Neural Networks (Computer)”/
9) Risk Assessment/
10) Models, Statistical/
11) 7 or 8 or 9 or 10
12) 6 and 11
13) Limit 12 to English language
14) Limit 13 to abstracts

For peer review only

Supplementary File 2: Search results for independent studies that did external validation for included models.

Study	No. of citations in Google Scholar	No. of citations in Medline	No. of external validation found in Google Scholar	No. of external validation found in Medline
Carlson, 1998	117	20	Null	Null
Babaian, 2000	128	13	Null	Null
Jansen, 2010	231	44	Null	Null
Hill, 2010	5	0	Null	Null
Lazzeri, 2013	195	0	Null	Null

For peer review only

Supplementary File 3: Type of assays used to measure PSA and free PSA.

Study	PSA Assay	Free PSA Assay
Carlson, 1998 [1]	PA immunoassay (Tosoh)	Investigational doubleantibody radioimmunometric assay (PSA II, DIANON Systems)
Babaian, 2000 [2]	PSA immunometric assay (Tosoh)	Tandem R assay (Beckman Coulter, San Diego, Calif)
Jansen, 2010 [3]	Access 2 Immunoassay System (Beckman Coulter, Brea, CA, USA)	Access 2 Immunoassay System (Beckman Coulter, Brea, CA, USA)
Hill, 2013 [4]	N/A	N/A
Lazzeri, 2013 [5]	Access 2 Immunoassay System (Beckman Coulter, Brea, CA, USA)	Access 2 Immunoassay System (Beckman Coulter, Brea, CA, USA)

References:

1. Carlson GD, Calvanese CB, Partin AW. An algorithm combining age, total prostate-specific antigen (PSA), and percent free PSA to predict prostate cancer: Results on 4298 cases. *Urology* 1998;52:455-61. doi: 10.1016/S0090-4295(98)00205-2
2. Babaian RJ, Fritsche H, Ayala A, et al. Performance of a neural network in detecting prostate cancer in the prostate-specific antigen reflex range of 2.5 to 4.0 ng/mL. *Urology* 2000;56:1000-6.
3. Jansen FH, van Schaik RHN, Kurstjens J, et al. Prostate-Specific Antigen (PSA) Isoform p2PSA in Combination with Total PSA and Free PSA Improves Diagnostic Accuracy in Prostate Cancer Detection. *European Urology* 2010;57:921-27. doi: 10.1016/j.eururo.2010.02.003
4. Hill OT, Mason TJ, Schwartz SW, et al. Improving prostate cancer detection in veterans through the development of a clinical decision rule for prostate biopsy. *BMC urology* 2013;13 doi: 10.1186/1471-2490-13-6
5. Lazzeri M, Haese A, de la Taille A, et al. Serum Isoform [−2]proPSA Derivatives Significantly Improve Prediction of Prostate Cancer at Initial Biopsy in a Total PSA Range of 2–10 ng/ml: A Multicentric European Study. *European Urology* 2013;63:986-94. doi: 10.1016/j.eururo.2013.01.011

For peer review only

Reporting checklist for systematic review and meta-analysis.

Based on the PRISMA guidelines.

Instructions to authors

Complete this checklist by entering the page numbers from your manuscript where readers will find each of the items listed below.

Your article may not currently address all the items on the checklist. Please modify your text to include the missing information. If you are certain that an item does not apply, please write "n/a" and provide a short explanation.

Upload your completed checklist as an extra file when you submit to a journal.

In your methods section, say that you used the PRISMAreporting guidelines, and cite them as:

Moher D, Liberati A, Tetzlaff J, Altman DG, The PRISMA Group. Preferred Reporting Items for Systematic Reviews and Meta-Analyses: The PRISMA Statement

		Reporting Item	Page Number
**Title**			
	#1	Identify the report as a systematic review, meta-analysis, or both.	3
**Abstract**			
Structured summary	#2	Provide a structured summary including, as applicable: background; objectives; data sources; study eligibility criteria, participants, and interventions; study appraisal and synthesis methods; results; limitations; conclusions and implications of key findings; systematic review registration number	3-4
**Introduction**			
Rationale	#3	Describe the rationale for the review in the context of what is already known.	5-6
Objectives	#4	Provide an explicit statement of questions being addressed with	6

For peer review only

		reference to participants, interventions, comparisons, outcomes, and study design (PICOS).	
**Methods**			
Protocol and registration	#5	Indicate if a review protocol exists, if and where it can be accessed (e.g., Web address) and, if available, provide registration information including the registration number.	n/a
Eligibility criteria	#6	Specify study characteristics (e.g., PICOS, length of follow-up) and report characteristics (e.g., years considered, language, publication status) used as criteria for eligibility, giving rational	6
Information sources	#7	Describe all information sources in the search (e.g., databases with dates of coverage, contact with study authors to identify additional studies) and date last searched.	6
Search	#8	Present full electronic search strategy for at least one database, including any limits used, such that it could be repeated.	6
Study selection	#9	State the process for selecting studies (i.e., for screening, for determining eligibility, for inclusion in the systematic review, and, if applicable, for inclusion in the meta-analysis).	7
Data collection process	#10	Describe the method of data extraction from reports (e.g., piloted forms, independently by two reviewers) and any processes for obtaining and confirming data from investigators.	7
Data items	#11	List and define all variables for which data were sought (e.g., PICOS, funding sources), and any assumptions and simplifications made.	8
Risk of bias in individual studies	#12	Describe methods used for assessing risk of bias in individual studies (including specification of whether this was done at the study or outcome level, or both), and how this information is to be used in any data synthesis.	8
Summary measures	#13	State the principal summary measures (e.g., risk ratio, difference in means).	7
Planned methods of analyis	#14	Describe the methods of handling data and combining results of studies, if done, including measures of consistency (e.g., I2) for each meta-analysis.	6-8
Risk of bias	#15	Specify any assessment of risk of bias that may affect the	15-16

For peer review only

across studies		cumulative evidence (e.g., publication bias, selective reporting within studies).	
Additional analyses	#16	Describe methods of additional analyses (e.g., sensitivity or subgroup analyses, meta-regression), if done, indicating which were pre-specified.	n/a
**Results**			
Study selection	#17	Give numbers of studies screened, assessed for eligibility, and included in the review, with reasons for exclusions at each stage, ideally with a flow diagram.	8-9
Study characteristics	#18	For each study, present characteristics for which data were extracted (e.g., study size, PICOS, follow-up period) and provide the citation.	8-9
Risk of bias within studies	#19	Present data on risk of bias of each study and, if available, any outcome-level assessment (see Item 12).	15-17
Results of individual studies	#20	For all outcomes considered (benefits and harms), present, for each study: (a) simple summary data for each intervention group and (b) effect estimates and confidence intervals, ideally with a forest plot.	13-15
Synthesis of results	#21	Present the main results of the review. If meta-analyses are done, include for each, confidence intervals and measures of consistency.	12-15
Risk of bias across studies	#22	Present results of any assessment of risk of bias across studies (see Item 15).	15-17
Additional analysis	#23	Give results of additional analyses, if done (e.g., sensitivity or subgroup analyses, meta-regression [see Item 16]).	n/a
**Discussion**			
Summary of Evidence	#24	Summarize the main findings, including the strength of evidence for each main outcome; consider their relevance to key groups (e.g., health care providers, users, and policy makers	18-21
Limitations	#25	Discuss limitations at study and outcome level (e.g., risk of bias), and at review level (e.g., incomplete retrieval of identified research, reporting bias).	21-22

For peer review only

Conclusions	#26	Provide a general interpretation of the results in the context of other evidence, and implications for future research.	23

Funding

Funding	#27	Describe sources of funding or other support (e.g., supply of data) for the systematic review; role of funders for the systematic review.	24

The PRISMA checklist is distributed under the terms of the Creative Commons Attribution License CC-BY. This checklist was completed on 30. September 2019 using https://www.goodreports.org/, a tool made by the EQUATOR Network in collaboration with Penelope.ai

For peer review only
